# Bonding and Reactivity of Germanium Enolates toward Group 14 Halides

**DOI:** 10.1021/acs.inorgchem.5c04482

**Published:** 2026-01-27

**Authors:** Nilanjana Sen, Manfred Drusgala, Roland C. Fischer, Dmytro Neshchadin, Thomas Lainer, Michael Haas

**Affiliations:** † Institute of Inorganic Chemistry, Graz University of Technology, Stremayrgasse 9/IV, 8010 Graz, Austria; ‡ Institute of Physical and Theoretical Chemistry, Graz University of Technology, Stremayrgasse 9/II, 8010 Graz, Austria

## Abstract

We report the reactivity of triacylgermenolate (**1**) and geminal bisgermenolate (**13**) toward group 14 halides under mechanochemical conditions, enabling efficient access to novel acylgermanes. Reactions with halosilanes afforded a series of silyl-substituted acylgermanes (**3**–**7**), while treatment with chlorogermanes and chlorostannanes yielded Ge–Ge and Ge–Sn bonded acylgermanes (**8**–**11**). In contrast, the bisgermenolate displayed divergent reactivity, forming unprecedented four-membered Ge–Ge and Ge–Sn ring systems (**15**, **16**). UV–Vis spectroscopy revealed pronounced bathochromic shifts and enhanced absorption for the heavier congeners, with photo-CIDNP experiments confirming α-cleavage and radical generation from the cyclic Ge–Ge compound. Overall, these findings establish triacyl- and bisgermenolates as versatile building blocks for accessing novel acylgermanes with tunable bonding motifs and photochemical properties.

## Graphical abstract

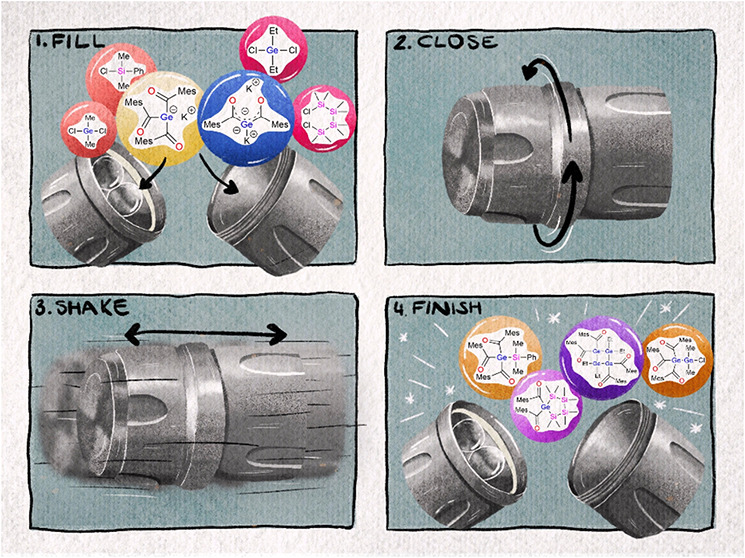

## Introduction

In recent years, the development of heavier group 14 (HG 14) enolates has garnered significant attention. In particular, germanium (Ge) and tin (Sn) derivatives are emerging as promising building blocks for high-performance free-radical photoinitiators.^1–4^ Historically, the synthesis and characterization of HG 14 enolates were primarily motivated by fundamental investigations in main group chemistry.^5^ Although a broad array of organic transformations is well-known involving carbon-based enolates,^6,7^ the higher congeners like silenolates, germenolates and stannenolates remained less explored for an extended period of time.

A significant breakthrough was achieved in 1989, when Bravo-Zhivotovskii and co-workers reported the first isolation of HG 14 enolates.^8^ This marked the very beginning for expanding their chemistry. Two principal resonance structures can be considered: the enol form, in which the negative charge resides on the oxygen atom, and the keto form, where the negative charge is localized on the central atom. Most enolates prefer the enol form in both solid state and in solution.^9^ In contrast, silenolates,^5,10^ germenolates^11^ and stannenolates^4,12^ exhibit markedly different resonance behavior, favoring the keto form as the more stable resonance structure (see Scheme 1). The only exception was reported by Apeloig and co-workers, who obtained a silenolate that preferentially adopts the enol form in the solid state.^13^

**Scheme 1:**
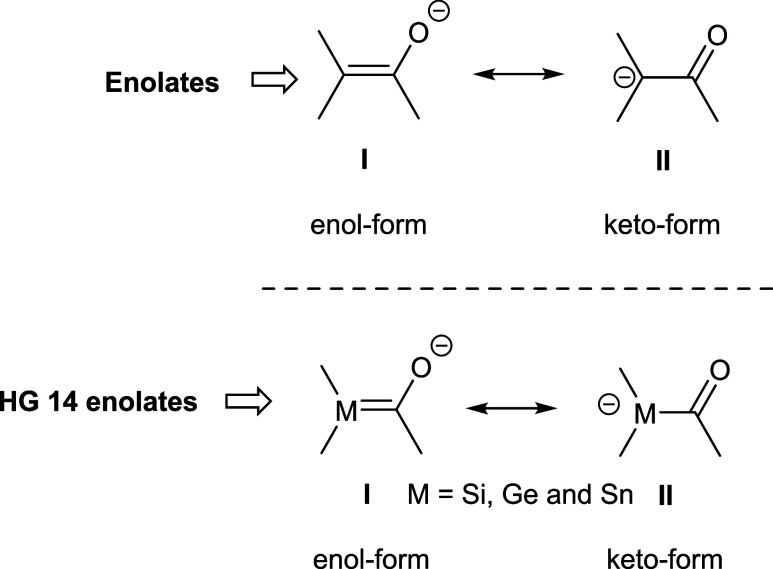
Resonance Structures of Enolates and HG 14 Enolates

The development of triacylgermenolates provided a versatile platform for the synthesis of novel high performance HG 14 photoinitiators.^14^ Along with this, our group also documented the synthetic protocol of the first geminal bisgermenolate, which will also serve as a new highly reactive building block in the field of main group chemistry (see Figure 1).^15^

**Figure 1:**
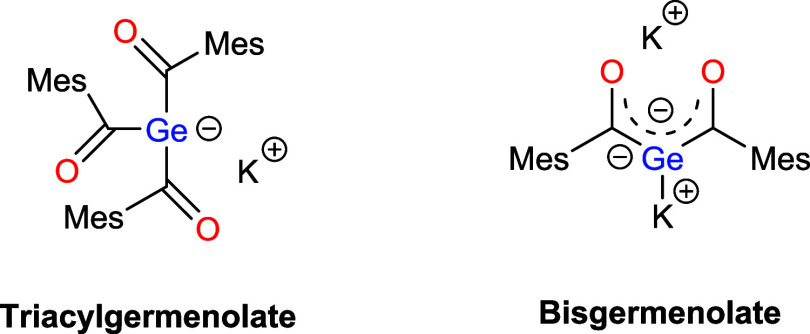
Triacylgermenolate and geminal bisgermenolate as nucleophiles for this work.

Based on these developments, we explored the reactivity of these two compounds with various heavier group 14 element electrophiles to access novel acyl metalloids.

## Results and Discussion

We started with triacylgermenolate **1** and reacted it with a series of halosilanes, evaluating a representative set of mono- and dichlorosilanes of varying steric and electronic demand. Having established the outcome for silicon electrophiles, we then turned to the heavier congeners and investigated chlorogermanes under analogous conditions. Finally, to complete the series, we extended our reactivity survey to include a representative halostannane.

### Reaction of 1 with Halosilanes under Homogeneous Conditions

The reaction of triacylgermenolate **1** with various halosilanes (i.e., TMSCl and Me_4_Si_2_Cl_2_) in THF at different temperatures revealed unexpectedly unselective reactivity. To address this, we varied the solvent polarity (e.g., employing toluene), which led to only a moderate improvement in selectivity. In all cases, besides the wanted product, we observed a competing metal–halide exchange, yielding the corresponding triacylhalogermane together with uncharacterized polysilanes (Scheme 2).

**Scheme 2:**
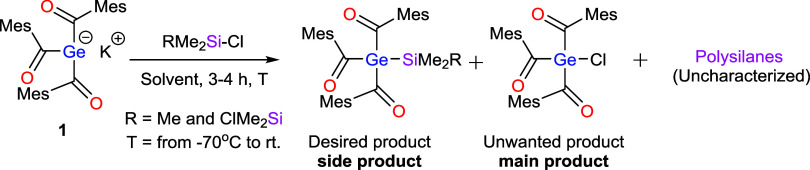
Reactivity of **1** with Chloro-Silanes in the Conventional Method

However, when **1** was reacted with TMSI in benzene at room temperature, no salt metathesis reaction was observed; instead, an exclusive metal–halide exchange occurred, affording compound **2** as the sole product in 51% yield (Scheme 3). Formation of **2** was confirmed by NMR spectroscopy as well as single-crystal X-ray diffraction. In the ^13^C NMR spectrum, the presence of the resonance at δ 227 ppm clearly indicates the presence of the acyl functionality in **2**. The molecular structure reveals that **2** crystallizes in the monoclinic space group *P*2_1_/2 (Figure 2). The Ge–I bond length of 2.504(5) Å is comparable to that observed in Me_3_GeI 2.544(1) Å.^16^ All other bond lengths and angles correspond well to the values typically found in organogermanes (Figure 2).

**Scheme 3:**
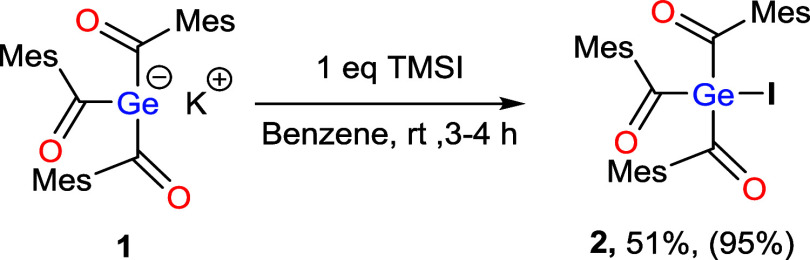
Reactivity of Compound **1** with TMSI under Conventional Conditions^a^

a Yields are given as crude (in parentheses) and isolated (outside parentheses).

**Figure 2:**
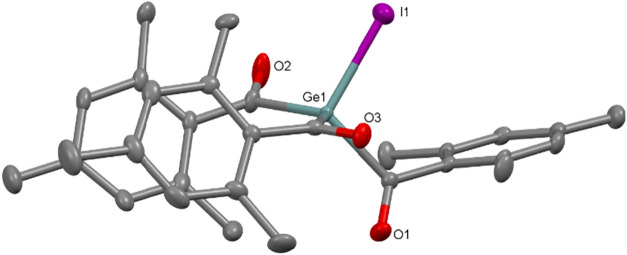
ORTEP representation for compound **2**. Thermal ellipsoids are depicted at the 50% probability level. Hydrogen atoms are omitted, and mesityl groups are displayed as wireframes for clarity. Selected bond lengths (Å) of **3** with estimated standard deviations: Ge(1)–I(1) 2.5035(5), Ge(1)–C(1) 2.043(4), Ge(1)–C(11) 2.052(3), Ge(1)–C(21) 2.040(3), C(1)–O(1) 1.217(4), C(11)–O(2) 1.202(4), C(21)–O(3) 1.205(4).

As the solution-phase attempts suffered from competing redox pathways and modest chemoselectivity, we implemented solvent-free mechanochemical conditions (ball milling). Under these conditions, the reaction proceeds predominantly via salt metathesis with high effective reactant concentration and localized, impact-driven activation, delivering the targeted acylgermanes in good to excellent isolated yields (SI, Figure S1). These effects are consistent with established features of mechanochemistry–enhanced chemoselectivity in the absence of solvent, phase-controlled reactivity, and pressure-dominated driving forces in ion-exchange reactions.^17–19^

### Reaction of 1 with Halosilanes in the Ball Mill

We initiated our investigation with various halosilanes, including PhMe_2_SiCl, TMSCl, TMSI, Me_4_Si_2_Cl_2_, Me_6_Si_3_Cl_2_ and Me_8_Si_4_Cl_2_. Compound **1** was treated with monochlorosilanes or with dichlorosilanes in the ball mill for 1 h, affording the desired silyl-substituted acylgermanes. After completion of the reaction, benzene was added to the yellow reaction slurries and the mixtures were filtered through a syringe filter to remove the formed salts. The solvents were then removed under vacuum to afford yellow oils. These crude products were dissolved in dry acetonitrile and stored at −30 °C, leading to crystallization of the silyl-substituted acylgermanes (Scheme 4).

**Scheme 4:**
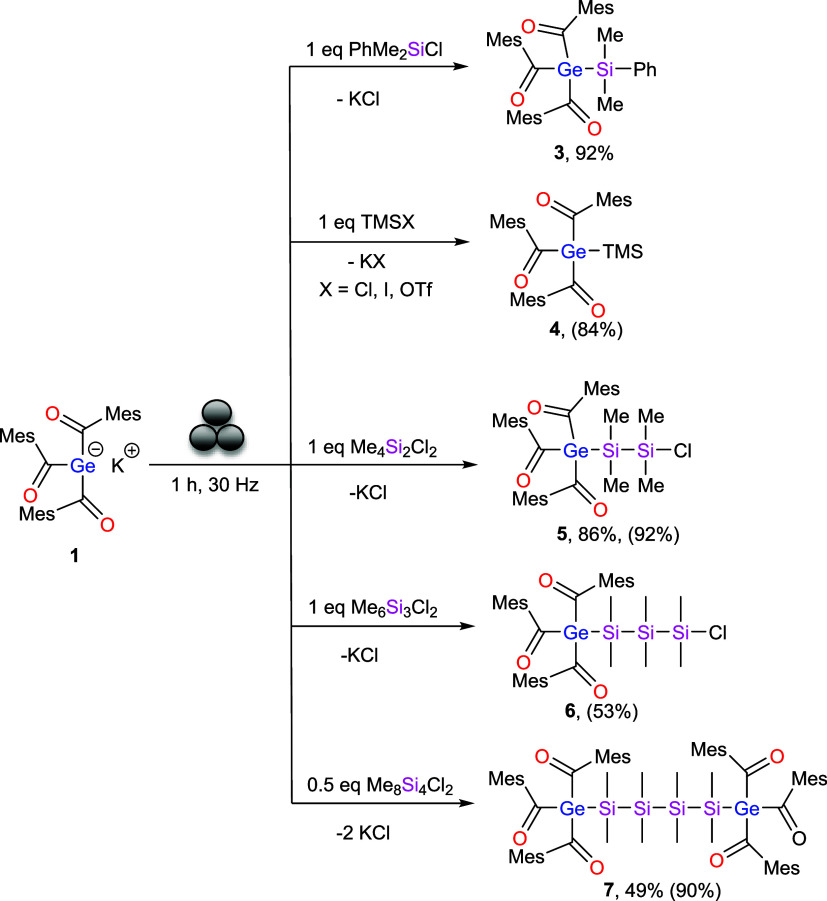
Reactivity of **1** with Halosilanes^a^

a Yields are given as crude (in parentheses) and isolated (outside parentheses).

To our surprise, we found that all silyl substituted acylgermanes are hydrolytically unstable and form the hydride substituted acylgermane (**Ge–H**) and the corresponding siloxanes (Scheme 5). Consequently, all manipulations were performed under inert conditions. We assume that a silyl migration might be responsible for this instability.

**Scheme 5:**
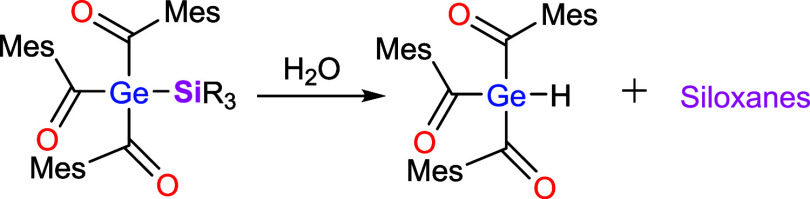
Reactivity of Silyl Substituted Acylgermanes with H_2_O

The formation of all compounds was confirmed by ^1^H, ^13^C and ^29^Si NMR spectroscopy. After precipitation with acetonitrile compound **3** was isolated as a yellow crystalline solid in good yield. In the ^1^H NMR of **3**, we observe a sharp singlet at δ 0.58 ppm, which corresponds to 6H for the methyl groups attached to the silicon atom. Two additional singlets appear at δ 2.00 ppm (9H) and 2.10 ppm (18H) attributable to the methyl groups of the mesityl substituents. A singlet at δ 6.41 ppm integrates for six protons and is assigned to the aromatic protons of the mesityl groups. In the ^13^C NMR spectrum, the carbonyl carbons resonate significantly downfield at δ 238 ppm (in comparison to tetraacylgermanes δ 233 ppm). In the ^29^Si NMR, a sharp singlet is observed at δ −8.5 ppm.

Interestingly, the reaction of **1** with TMSX (X = Cl, I and OTf) afforded the silyl-substituted acylgermane **4**, which, however, proved to be highly unstable even under inert conditions. At room temperature, rapid degradation occurred within 8 h, and noticeable decomposition was also observed at −30 °C within 24 h. The only characterizable degradation product was identified by ^1^H and ^13^C NMR as the corresponding hydridogermane, accompanied by a substantial amount of polymeric material. In addition, ^29^Si NMR revealed signals attributable to uncharacterized siloxanes. This instability was unexpected, prompting a closer investigation of the degradation process. Consequently, the reaction was performed as outlined above, but this time, to ensure minimal degradation, no workup or reagent removal was carried out. Under these conditions, the NMR spectra showed a clean and selective conversion to the expected product **4**. A series of ^1^H, ^13^C and ^29^Si NMR measurements was then carried out to monitor the degradation pathway over time. After approximately 15 min, an additional set of resonances emerged, reaching maximum intensity after 120–180 min. Based on these NMR data, we assigned the new species to a Brook-type germene **4b** (see Scheme 6). A ^13^C signal characteristic of a Ge=C double bond was observed at δ = 208 ppm.^1,11^ In the carbonyl region, two closely spaced resonances appeared, consistent with restricted rotation around the germanium–carbon double bond. In addition, the ^1^H and ^13^C spectra display sharp and distinct resonances for each of the three mesityl groups, reflecting their magnetic nonequivalence in the presence of the double bond. The ^29^Si NMR spectra showed a corresponding trend: besides the signal for compound **4** at δ = −3.4 ppm a second SiMe_3_ signal at δ = 21.0 ppm appeared, indicating the formation of an OSiMe_3_ group (see Figure 3).

**Scheme 6:**
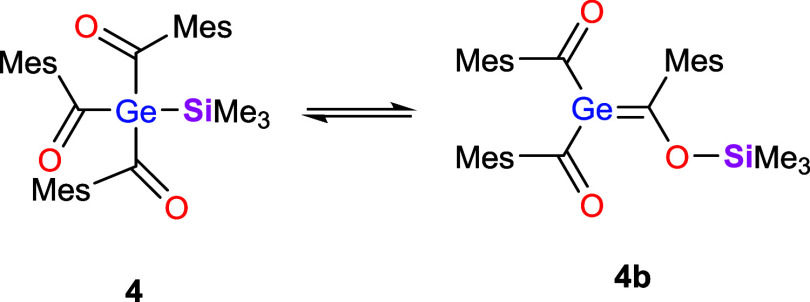
Keto-Enol Equilibrium of **4**

**Figure 3:**
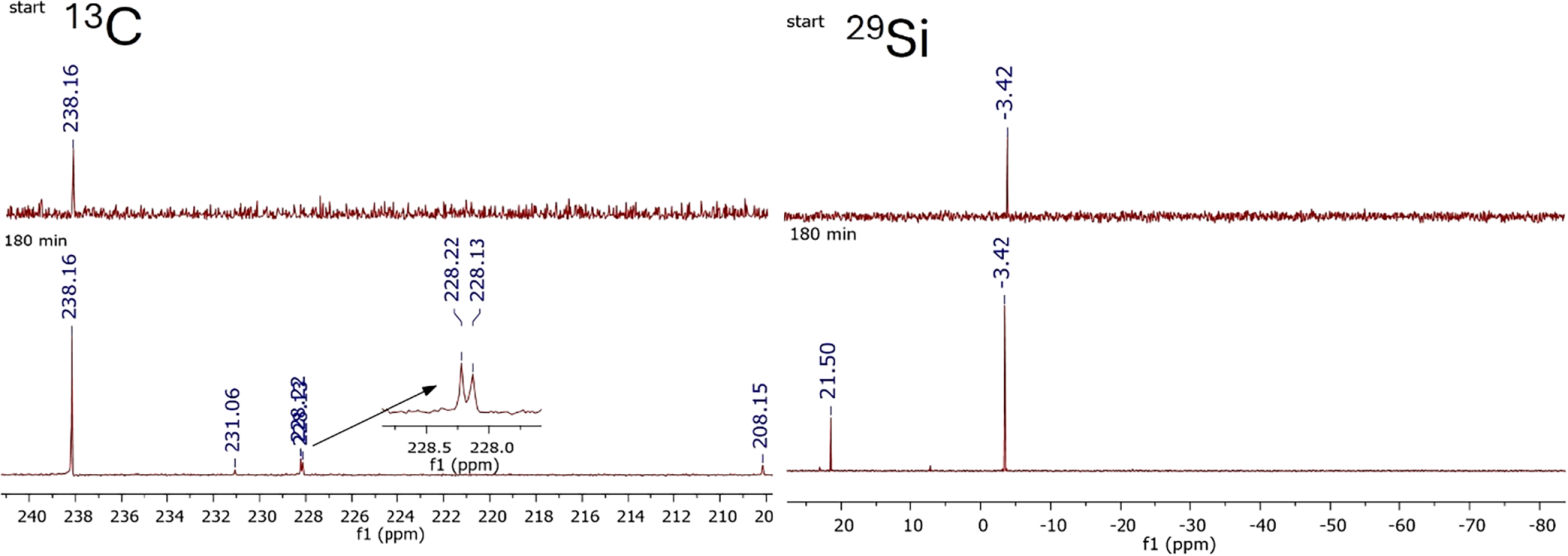
Stacked ^13^C and ^29^Si NMR spectra recorded at the start of the experiment (top traces) and after 180 min (bottom traces), allowing direct comparison of chemical changes over time. For clarity, only the relevant spectral regions are shown.

Together, these observations indicate that compounds **4** and **4b** are connected through a reversible, keto–enol-type equilibrium involving migration of the silyl group and formation of the Ge=C double bond (see Scheme 6).

Upon prolonged measurement time in all regions of the ^1^H spectrum broad polymeric signals appeared, which grew on intensity every hour. After approximately 14 h almost all of compound **4** and **4b** are converted to uncharacterizable polymer (see Figure S40 for the complete measurement). We assume that the compound **4b** is unstable and simply undergoes an unknown degradation pathway.

In order to intercept the Brook-type germene **4b** we performed a trapping with MeOH at different reaction times of the reaction. However, in all case the hydride substituted acylgermane (**Ge–H**) was obtained as sole product.

Furthermore, we explored the reactivity of **1** with three dichlorosilanes. Treatment of **1** with 0.5 equiv of Me_4_Si_2_Cl_2_ in the ball-mill for 1 h afforded exclusively the monosubstituted product, with no indication of double substitution. Even after prolonged milling (8 h), no change was observed. When the reaction was carried out with equimolar amounts of Me_4_Si_2_Cl_2_ under the same conditions (1 h), the monosubstituted product **5** was obtained in 86% yield. The ^1^H NMR spectrum of **5** displays two sharp singlets at δ 0.52 and δ 0.76 ppm, integrating for 12 protons of the methyl groups attached to the silicon atoms. The sharp resonances at δ 2.00 and δ 2.10 ppm are attributable to the 9H and 18H of the mesityl substituents, while a singlet at δ 6.45 ppm corresponds to the aromatic protons. In the ^13^C NMR, a resonance at δ 237 ppm is observed for the carbonyl carbons. The ^29^Si NMR spectrum of **5** displays two signals: one at δ −29.16 ppm for the central silicon atom attached to the germanium center and one at δ 27.90 ppm for the SiMe_2_Cl moiety.

We also investigated whether an enol tautomer analogous to **4b** is present in compound **5**. However, no characteristic resonances attributable to a Ge=C enol form were detected in any NMR experiment. Similar results were obtained for all other silyl-substituted acylgermanes studied.

Single-crystal X-ray diffraction confirmed the structure of **5**, which crystallizes in the monoclinic space group *P*2_1_/*c* (Figure 4). The bond lengths between Ge–Si is 2.4067(6) Å, which is within the range of previously reported Ge–Si single bond, which is 2.474(2) Å in [(CH_3_)_3_Si_3_SiGeCl]_2_ ^20^ and Si–Si and Si–Cl bond lengths are 2.338(7) is 2.097(6) Å respectively.

**Figure 4:**
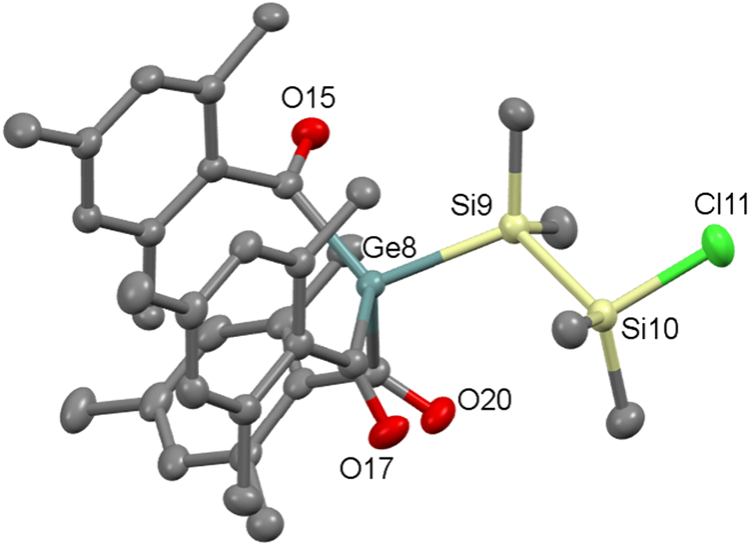
ORTEP representation for compound **5**. Thermal ellipsoids are depicted at the 50% probability level. Hydrogen atoms are omitted for clarity. Selected bond lengths (Å) of **5** with estimated standard deviations: Ge1–Si1 2.4067(6), Si1–Si2 2.338(7), Ge1–C11 2.041(2) Ge1–C21 2.046(2), Ge1–C1 2.040(2).

Encouraged by these results, we next investigated the reactivity of **1** with a longer silicon chain by treatment with Me_6_Si_3_Cl_2_ (Scheme 4). Once again, only the monosubstituted acylgermane **6** was obtained. We confirmed the formation of this compound only by NMR spectroscopy. The resonance of the carbonyl group appeared at δ 238 ppm, providing strong evidence for the formation of **6**. However, the ^1^H NMR and ^29^Si NMR spectra revealed numerous side products, which are mainly different siloxanes (See SI, Figures S12 and S14).

Subsequently, **1** was treated with Me_8_Si_4_Cl_2_, which resulted in the abstraction of both chlorides and the formation of the disubstituted hexaacyldigermane **7**. The ^13^C NMR spectrum displayed a resonance at δ 238 ppm, confirming the presence of the carbonyl carbons of **7**. The ^29^Si NMR exhibited two singlets: one at δ – 22.23 ppm, assigned to the silicon atoms bound to germanium, and another at δ −32.56 ppm, corresponding to the two central silicon atoms.

In addition, **1** was also treated with Cl_2_SiPh_2_, Cl_2_SiMes_2_, HSiCl_3_, SiCl_4_ or SiBr_4_, which all led to formation of [MesC­(O)]_3_GeCl and [MesC­(O)]_3_GeBr, respectively.

### UV–vis Spectroscopy of 3, 5 and 7

UV–vis spectroscopy was employed to gain insights into the electronic properties and light absorption behavior of the compounds, which are directly relevant to their potential as photoinitiators. THF was chosen as the solvent for the UV–vis study. The three isolable silyl-substituted acylgermanes were compared with the hydride-substituted acylgermane (**Ge–H**). The spectra reveal distinct differences between the silyl derivatives and the **Ge–H** reference compound. **Ge–H** exhibits a strong absorption band in the visible region, which serves as the baseline for comparison. Upon silyl substitution, pronounced bathochromic shifts were observed. The intensity of the absorptions is also enhanced, indicating stronger electronic transitions associated with the extended conjugation and the electron-withdrawing effect of the acyl substituents (see Figure 5). These spectral changes underline the significant role of substitution in tuning the light absorption properties, underscoring the potential of these derivatives (once solved the issues with the instability in water) as visible-light photoinitiators.

**Figure 5:**
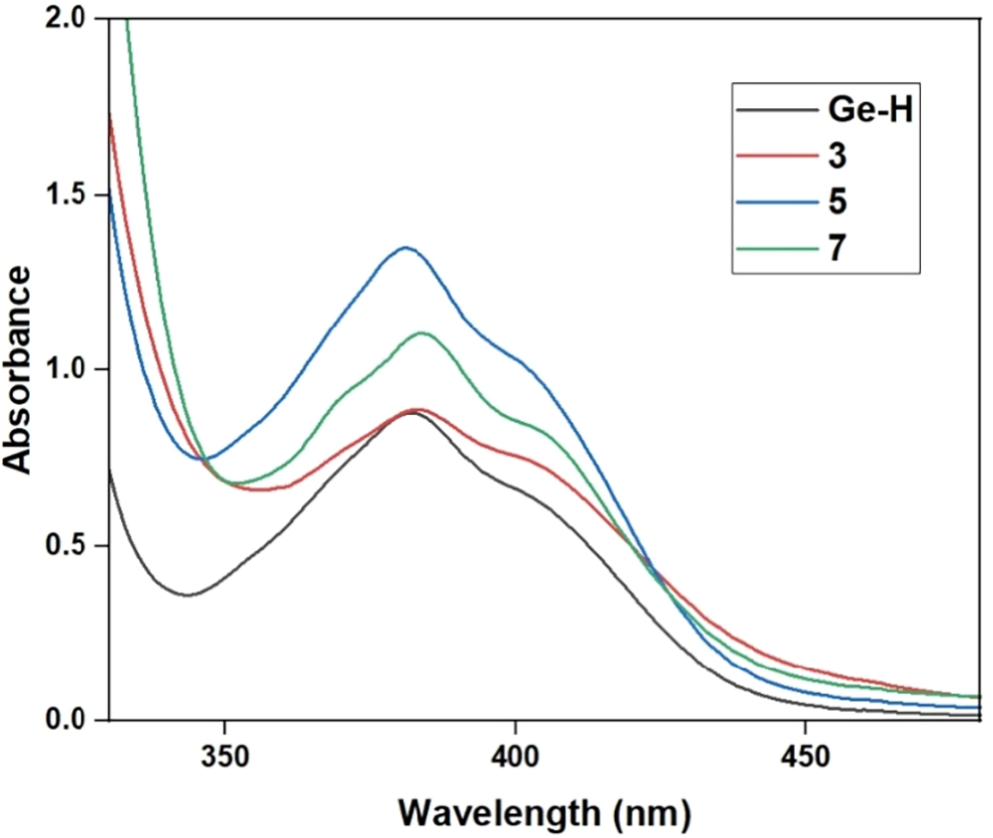
UV–vis absorption spectra of compounds **3**, **5** and **7** in THF in comparison with the hydride-substituted acylgermane (**Ge–H**) (c = 1 × 10^–3^ mol/L).

### Reaction of 1 with Chlorogermanes in the Ball Mill

After the successful transformations with halosilanes, we next examined the reactivity of **1** with various chlorogermanes. Treatment of **1** with Me_2_GeCl_2_, Et_2_GeCl_2_, Ph_2_GeCl_2_ and (*n*-Bu)_3_GeCl in the ball-mill for 1 h consistently resulted in the formation of new Ge–Ge bonds, accompanied by the elimination of KCl (Scheme 7). We also investigated the possibility of a keto–enol equilibrium for these compounds, but no enol formation was detectable.

**Scheme 7:**
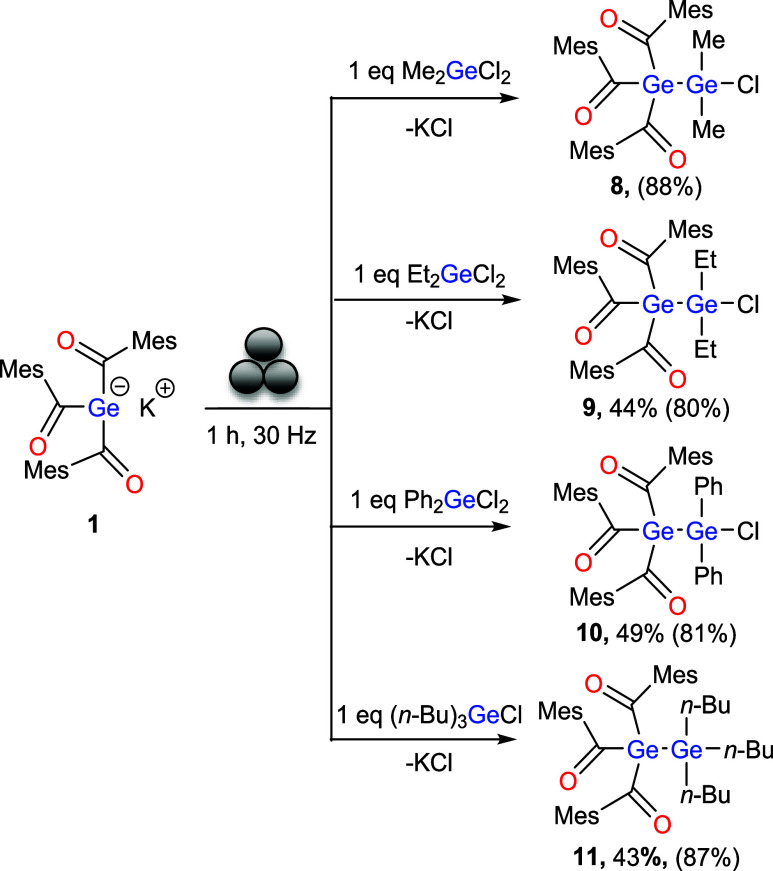
Reactivity of **1** with Chlorogermanes^a^

a Yields are given as crude (in parentheses) and isolated (outside parentheses).

Treatment of **1** with equimolar amounts of Me_2_GeCl_2_ resulted in the straightforward formation of compound **8**, as confirmed by the characteristic ^13^C carbonyl resonance at δ 233.47 ppm and a singlet at δ 0.80 ppm (6H) in the ^1^H NMR spectrum, corresponding to the methyl groups. In line with the results obtained for the silanes, no double substitution was observed. Compound **8** was obtained cleanly; however, no further characterization was carried out beyond the NMR assignment. The use of Me_2_GeCl_2_ was not pursued further due to its restricted commercial availability, whereas Et_2_GeCl_2_ was readily accessible. Moreover, given their close chemical similarity, further studies were carried out with the ethyl derivative. Compounds **9**–**11** were isolated in moderate yields (44, 49, and 43%, respectively). Their formation was confirmed by NMR spectroscopy and single-crystal X-ray diffraction studies.

The ^1^H NMR spectrum of **9** shows resonances at δ 0.91–1.45 ppm, consistent with the ethyl substituents, while **10** exhibits characteristic aromatic signals at δ 7.06–7.10 ppm, attributable to the phenyl groups. For compound **11**, resonances at δ 0.93–0.97 ppm and δ 1.18–1.40 ppm confirm the presence of the *n*-butyl groups. In the ^13^C NMR spectra of all compounds, resonances between δ 233 and 238 ppm are observed, characteristic of the carbonyl carbon atoms adjacent to the germanium center.

The single-crystal X-ray diffraction analysis confirms that in compounds **9** and **10** only one of the chloride atoms is abstracted. **9** crystallizes in monoclinic space group *P*2_1_/*c* (Figure 6), whereas **10** is found to crystallize in orthorhombic space group *P*2_1_2_1_2_1_ (Figure 7). The molecular structures reveal that the Ge–Ge bond length and Ge–Cl of both **9** and **10** are similar, which is 2.419(6) and 2.182(1) Å, 2.449(7) and 2.193(1) Å, respectively. **11** crystallizes in triclinic space group *P*-1 with Ge–Ge bond length of 2.443(3) Å (see Figure 8). The Ge–Ge bond lengths in our compounds are found to be shorter than the previously reported Ge–Ge single bond 2.509(1) Å in [(CH_3_)_3_Si_3_SiGeCl]_2_.^20^

**Figure 6:**
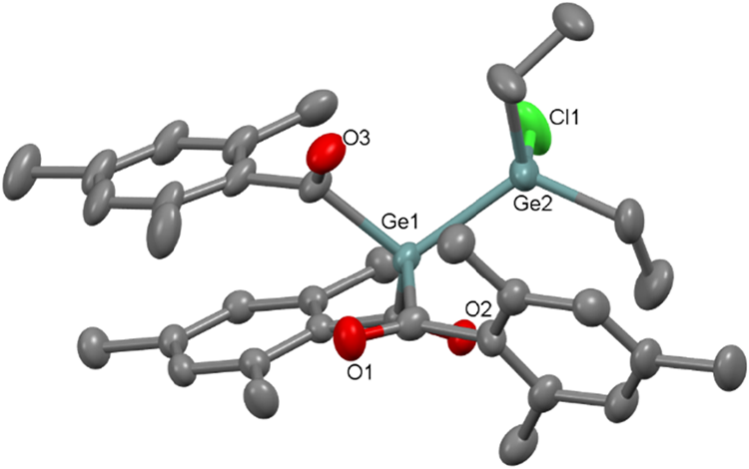
ORTEP representation for compound **9**. Thermal ellipsoids are depicted at the 50% probability level. Hydrogen atoms are omitted for clarity. Selected bond lengths (Å) of **9** with estimated standard deviations: Ge1–Ge2 2.4193(6), Ge1–C11 2.042(4), Ge1–C21 2.035(3), Ge1–C1 2.065(3).

**Figure 7:**
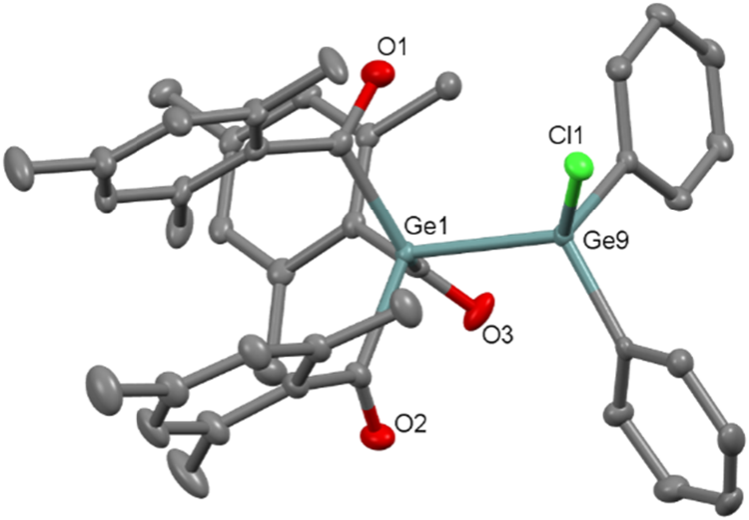
ORTEP representation for compound **10**. Thermal ellipsoids are depicted at the 50% probability level. Hydrogen atoms are omitted for clarity. Selected bond lengths (Å) of **10** with estimated standard deviations: Ge1–Ge9 2.449 (7), Ge9–Cl1 2.193(1), Ge1–C1 2.039(5), Ge1–C11 2.046(5), Ge1–C21 2.035(4).

**Figure 8:**
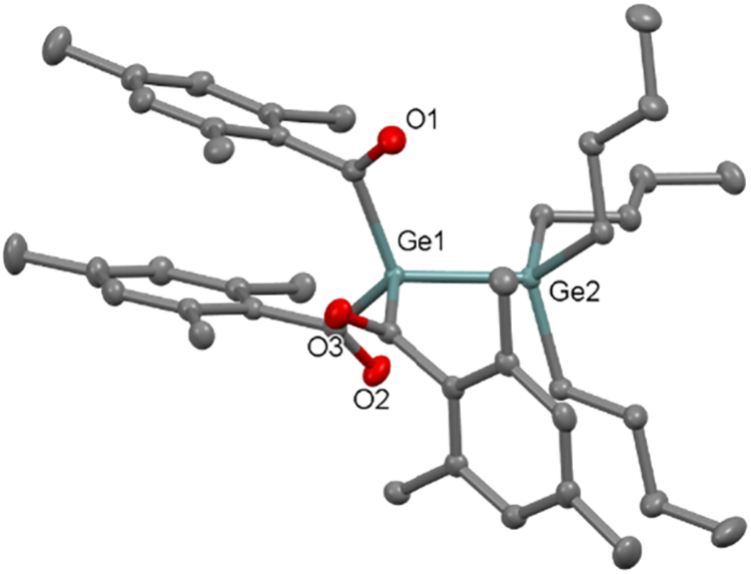
ORTEP representation for compound **11**. Thermal ellipsoids are depicted at the 50% probability level. Hydrogen atoms are omitted for clarity. Selected bond lengths (Å) of **11** with estimated standard deviations: Ge1–Ge2 2.443(3), Ge1–C1 2.040(1), Ge1–C11 2.051(2), Ge1–C21 2.050(2).

Surprisingly, all the germanium-substituted acylgermanes proved to be hydrolytically stable, with no evidence for the formation of hydride substituted acylgermane or other unstable side products.

Treatment of **1** with GeCl_2_·dioxane resulted in the formation of the (MesCO)_3_GeCl and the hydride-substituted acylgermane, along with unidentified products. In comparison, the reaction of **1** with GeCl_4_ afforded only (MesCO)_3_GeCl as the main product.

### Reaction of 1 with Chlorostannane in the Ball Mill

Treatment of **1** with (*n*-Bu)_3_SnCl exhibited a similar reactivity pattern, affording compound **12** in 39% yield. Structurally, **12** closely resembles compound **11** (Scheme 8). The ^13^C NMR spectrum shows a characteristic resonance at δ 238 ppm, corresponding to the carbonyl carbons directly attached to germanium center. In the ^119^Sn NMR a sharp singlet at δ – 65 ppm was observed.

**Scheme 8:**
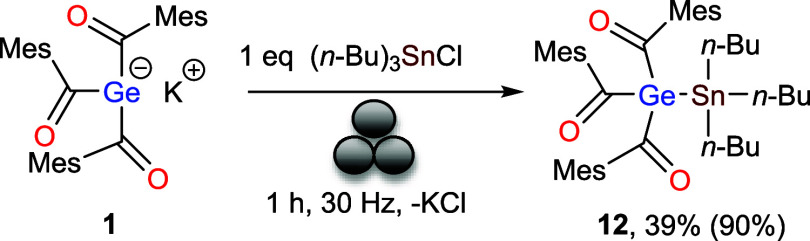
Reactivity of **1** with Chlorostannane^a^

a Yields are given as crude (in parentheses) and isolated (outside parentheses).

Single crystals of **12** suitable for X-ray diffraction study were obtained from acetonitrile at −30 °C. The molecular structure confirms that **12** also crystallizes in triclinic space group *P-*1 with Ge–Sn bond length of 2.608(3) Å, which is found to be very much similar to the previously reported compounds like Me_3_GeSnPh_3_ (2.599(3) Å) and Ph_3_GeSnMe_3_ (2.652(2) Å)^21^ (see Figure 9).

**Figure 9:**
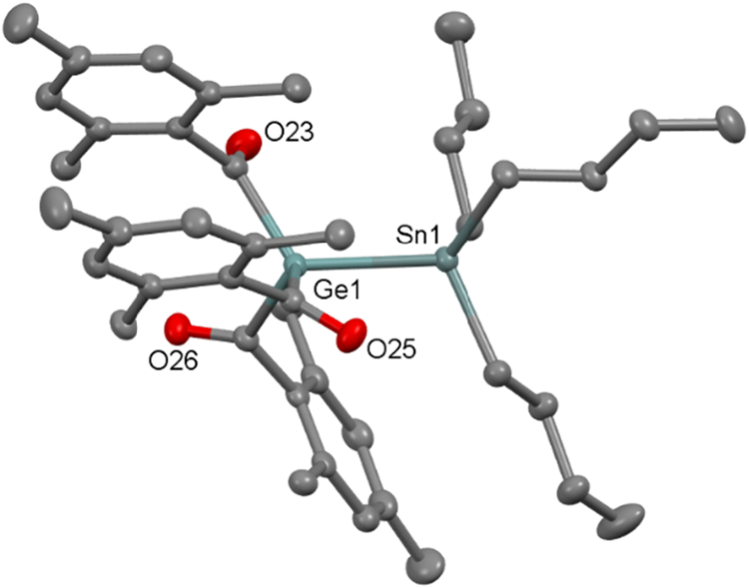
ORTEP representation for compound **12**. Thermal ellipsoids are depicted at the 50% probability level. Hydrogen atoms are omitted for clarity. Selected bond lengths (Å) of **12** with estimated standard deviations: Ge1–Sn1 2.608(3), Ge1–C1 2.050(2), Ge1–C11 2.038(2), Ge1–C21 2.052(2), Sn1–C39 2.160(2), Sn1–C35 2.169(2), Sn1–C31 2.159(2).

### UV–vis Spectroscopy of 9, 10, 11 and 12

To evaluate the influence of these novel substituents on the absorption behavior of the newly synthesized acylgermanes, we recorded the UV–Vis spectra of compound **9**–**12** (Figure 10). Introduction of the GeEt_2_Cl substituent in **9** leads to a clear increase in absorption intensity relative to **Ge–H**. In contrast, the phenyl-substituted derivative **10** shows a decrease in intensity, while the (*n*-Bu)_3_Ge derivative **11** closely resembles the **Ge–H** reference spectrum. The most pronounced effect is observed for the Sn-substituted compound **12**, which exhibits both significantly enhanced absorption intensity and a marked bathochromic shift compared to the Ge analogues. This behavior is attributed to the heavier atom effect of tin, which enhances spin–orbit coupling and facilitates stronger absorption in the visible region.

**Figure 10:**
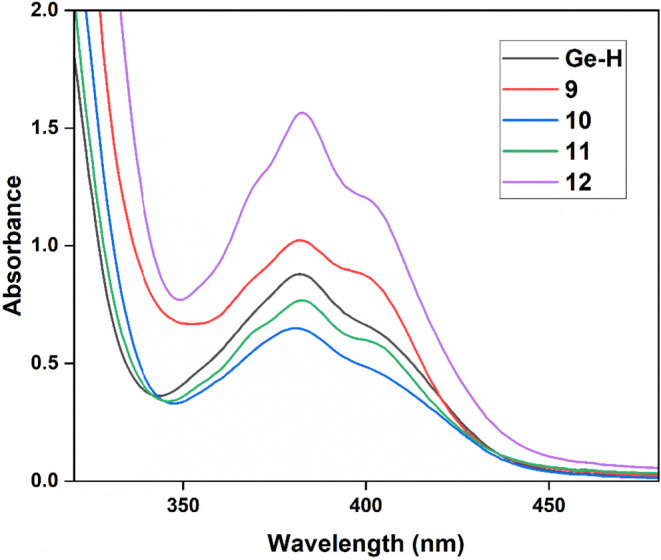
UV–vis absorption spectra of compounds **9**, **10**, **11** and **12** in THF in comparison with the hydrido substituted acylgermane (c = 1 × 10^–3^ mol/L).

After studying the reactivity of compound **1** with various group 14 halides, we next sought to explore the reactivity pattern of the geminal bisgermenolate **13** toward these electrophiles. The same experimental setup was applied for **13**. As before, we first examined its reactivity with halosilanes, followed by the heavier congeners, namely chlorogermanes and halostannanes, under analogous conditions.

### Reaction of 13 with Halosilanes in the Ball Mill

We first evaluated halosilanes as potential electrophiles. Surprisingly, however, no selective reaction was observed with any of the tested chlorosilanes (TMSCl, PhMe_2_SiCl, Me_2_SiCl_2_, Ph_2_SiCl_2_, *t*-BuMe_2_SiCl, Me_4_Si_2_Cl_2_). In all mentioned cases the reaction resulted in the formation of uncharacterized compounds along with the formation of siloxanes and polysilanes. This was quite surprising as carbon-based electrophiles react straightforwardly with the dianion.^15^

At this stage, we had almost abandoned the prospect of productive reactivity of the dianion with halosilanes. However, recalling one of our previous studies in which the geminal bisgermenolate reacted with 1,4-dibromobutane to afford a five-membered ring featuring germanium at the central position,^15^ we decided to test a structurally related 1,4-dichlorotetrasilane (Me_8_Si_4_Cl_2_). Milling **13** with Me_8_Si_4_Cl_2_ in a 1:1 ratio indeed resulted in the formation of a similar five-membered ring compound **14** with germanium at the center (Scheme 9).

**Scheme 9:**
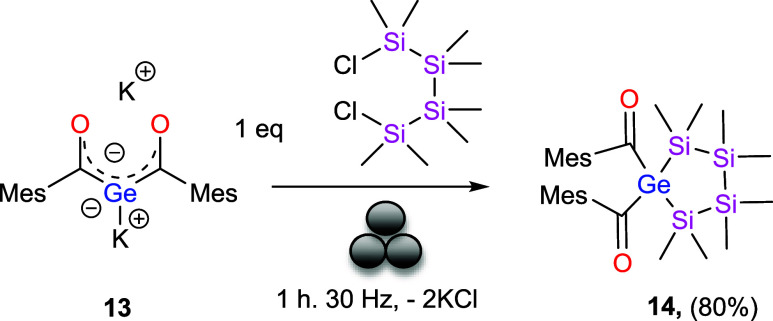
Reactivity of **13** with Dichlorosilane^a^

a Crude yield in parentheses.

The formation of **14** was confirmed by NMR spectroscopy. In the ^29^Si NMR spectrum, two resonances appeared at −22.6 and −39.3 ppm, while the ^13^C NMR spectrum showed a characteristic carbonyl signal at 243 ppm, consistent with the expected structure. Compound **14** could not be isolated in pure form due to its limited stability. Even under inert conditions, the compound gradually decomposed at room temperature and was no longer detectable after 48 h. We assume that here also the keto–enol is responsible for the instability. Attempts to purify **14** by aqueous workup resulted in immediate degradation, preventing its isolation in analytically pure form. This pronounced instability contrasts with the previously reported carbon-based five-membered acylgermane analogue, demonstrating the sensitivity of the silicon-linked system.

### Reaction of 13 with Halogermanes in the Ball Mill

We next evaluated the reactivity of the dianion toward halogermanes and halostannanes. In close analogy to the previously investigated halosilanes, unselective reactions occurred with Ph_2_GeCl_2_, (*n*-Bu)_3_GeCl, (*n*-Bu)_3_SnCl or Ph_2_SnCl_2_. In contrast, Et_2_GeCl_2_ and (*n*-Bu)_2_SnCl_2_ displayed a markedly different behavior. Both electrophiles reacted selectively with **13** to afford four-membered ring systems as the sole detectable products (Scheme 10). Notably, Mes_2_GeBr_2_ did not react, likely due to the steric hindrance imposed by the bulky mesityl substituents.

**Scheme 10:**
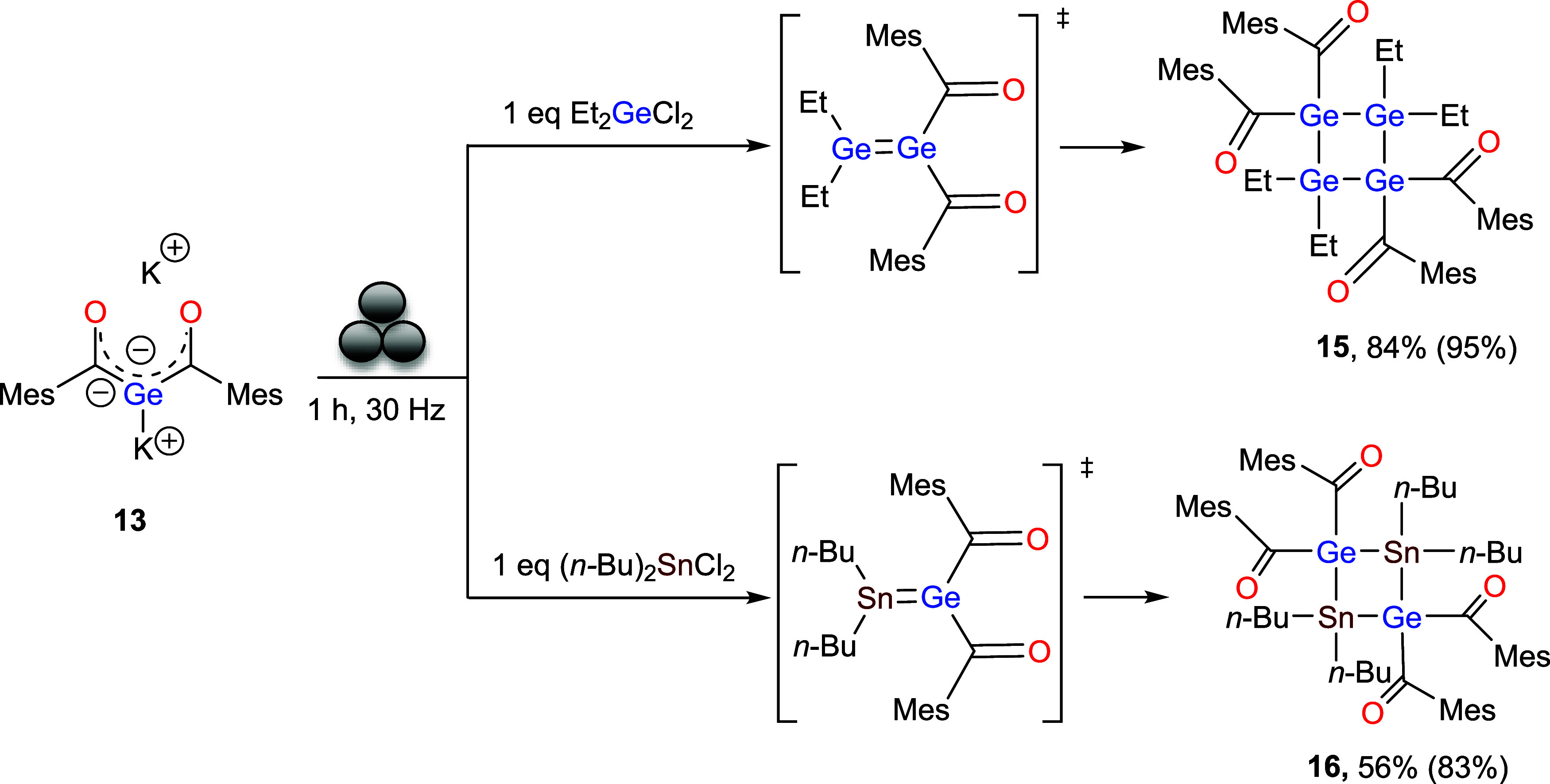
Reactivities of **13** with Dichloro-Germane and -Stannane^a^

a Crude yield in parentheses, isolated yield outside parentheses.

We were able to isolate yellow crystals of **15** in acetonitrile at room temperature in 84% yield. Compound **15** crystallizes in monoclinic space group *P*2_1_/*n* (Figure 11). The molecular structure confirms the formation of a four-membered ring, with Ge–Ge bond lengths of 2.459(5) and 2.4626(5) Å. These values are comparable to those reported for the four-membered ring (Ph_2_Ge)_4_ [2.465 Å] by Ross and Dräger,^22^ but significantly shorter than those observed for the cyclic germanium compound [FluTMSGeCl]_4_ [FluTMS = 9-trimethylsilyl-fluorenyl] described by Schnepf and co-workers [2.515(6)–2.518(6) Å].^23^ The constitution of **15** was further established by NMR spectroscopy. In the ^13^C NMR spectrum, the appearance of a peak at δ 234 ppm is diagnostic of the formation of **15**. Remarkably, this compound was found to be stable toward both acidic and basic aqueous workup. Such stability is particularly important for the envisioned application as photoinitiator, where resistance to hydrolytic degradation under diverse conditions will be crucial. Consequently, this compound was selected for further investigation by CIDNP (Chemically Induced Dynamic Nuclear Polarization) measurements.

**Figure 11:**
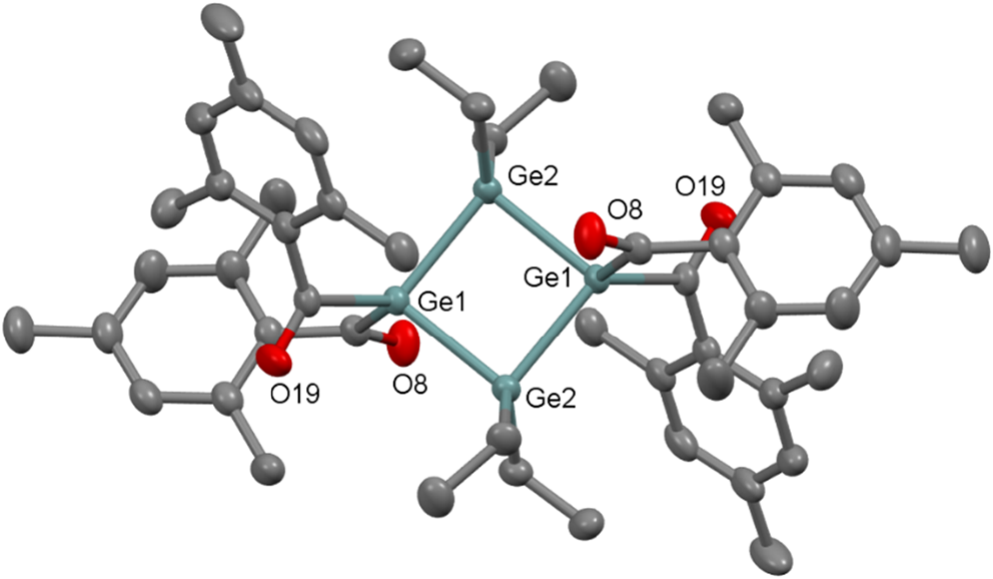
ORTEP representation for compound **15**. Thermal ellipsoids are depicted at the 50% probability level. Hydrogen atoms are omitted for clarity. Selected bond lengths (Å) of **15** with estimated standard deviations: Ge1–Ge2 2.459(5), Ge1–Ge2 2.462(5), Ge1–C1 2.049(1), Ge1–C11 2.042(3).

To evaluate the photoinitiating potential of **15**, we employed ^1^H photo-CIDNP spectroscopy,^24^ which can selectively detect even very inefficient free radical reactions. NMR and CINDP spectra of **15** in the presence of 5 times excess of butyl acrylate (M) as free radical quencher are shown in Figure 12. Although the CIDNP spectrum is largely dominated by background NMR signals of the starting compounds, several polarizations can nevertheless be clearly distinguished. The singlet in enhanced absorption at 10.01 ppm is unambiguously attributed to the aldehyde proton of mesityl aldehyde. This aldehyde is produced via hydrogen atom transfer from the product of the first addition (Ge-M•) to mesitoyl radical (MesC­(O)•).^25^ Ge-M• was in turn the result of the addition of germyl radical Ge• to a double bond of M. The second CIDNP signal at 4.85 ppm (enhanced emission) is due to the recombination of Ge-M• and MesC­(O)•. The doublet of doublets (4.85 ppm, dd, J_1_ = 10 Hz, J_2_ = 5.5 Hz) is assigned to the proton H_1_ (Figure 11) which is coupled to H_2_ and H_3_ that are diastereotopic. Via the observation of secondary reactions, the CIDNP spectra show that upon UV–vis irradiation **15** undergoes α-cleavage producing Ge• and MesC­(O)•. In turn, those radicals can add to a double bond of M initiating free radical polymerization. The similar CIDNP reactivity pattern was observed with other acylgermanes, however, we must mention that compared to tetraacylgermane^26^ CIDNP polarizations of **15** were much weaker, that can point on the lower efficiency of α-cleavage at our experimental conditions.

**Figure 12:**
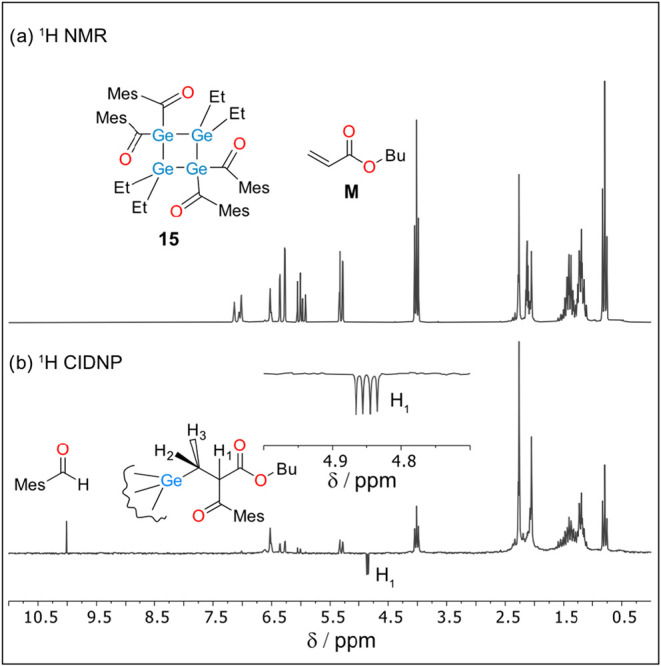
^1^H NMR (a, top) and photo-CIDNP (b, bottom) spectra of **15** in the presence of butyl acrylate (M) in Toluene-d_8_, using Hg/Xe high pressure UV lamp as the light source (flash ca. 300 ms).

Subsequently, a similar type of reaction was performed with (*n*-Bu)_2_SnCl_2_ and **13** under the same reaction conditions. This reaction afforded the four-membered cyclic compound **16**, which is structurally analogous to **15**, with tin atoms occupying two opposite corners of the ring and germanium atoms bearing carbonyl substituents at the remaining positions. The presence of the signal at 239 ppm in ^13^C NMR spectrum indicates the formation of the desired compound. **16** has also been isolated as yellow crystals in acetonitrile. Compound **16** crystallizes in triclinic, space group *P*-1 (see Supporting Information Figure S51).

The UV–vis absorption spectra for both **15** and **16** were compared with the hydride-substituted acylgermane **GeH**_**2**_, which served as a benchmark reference (Figure 13). Compound **15** shows enhanced intensity relative to **GeH**_**2**_, while the Ge–Sn analogue **16** displays an even stronger absorption intensity and a significant red shift, reflecting again the heavy atom effect of tin.

**Figure 13:**
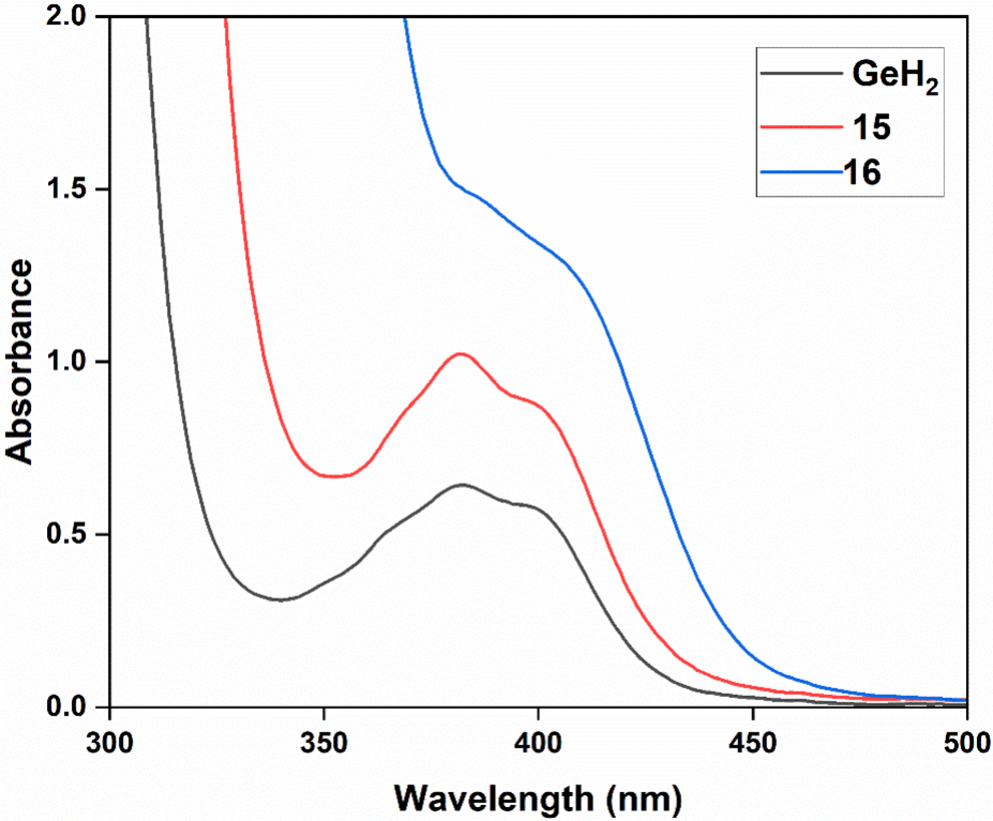
UV–vis absorption spectra of compounds **15** and **16** in THF (c = 1 × 10^–3^ mol/L).

The formation of four-membered rings such as **15** and **16** is particularly intriguing in the context of their potential reactivity. These strained cyclic systems can be viewed as surrogates for germylenes and stannylenes, as the ring strain and electron-rich germanium centers are expected to facilitate bond activation and nucleophilic reactivity reminiscent of low-valent E­(II) species. As such, these four-membered rings could serve as precursors to E­(II)-type reactivity under suitable conditions, opening opportunities for bond activation, coordination chemistry, and potential applications in catalysis or photoinitiated transformations.

To probe the potential germylene- or stannylene-type reactivity of the four-membered ring systems, we subjected the compounds to a series of test reactions. Addition of Et_3_SiH or BH_3_·THF under irradiation did not lead to any detectable transformation, and thermal activation up to 150 °C likewise failed to induce reactivity. In contrast, treatment of **15** with two equivalents of the N-heterocyclic carbene IMe_4_ at room temperature resulted in no observable change, but upon heating to 65 °C the four-membered ring cleaved to give the previously reported NHC-stabilized bisacylgermylene **17** (Scheme 11).^27^ It was not possible to determine the fate of the second germanium fragment. Multiple signals in the ^1^H as well as in the ^13^C NMR spectrum indicate an unselective reaction of this germylene.

**Scheme 11:**
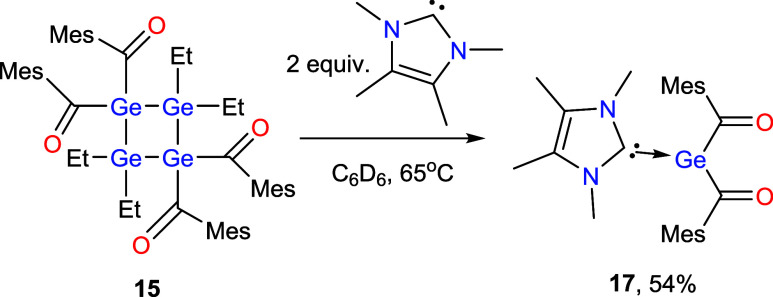
Reactivities of **15** with IMe_4_

In contrast, the Sn derivative did not exhibit the same selectivity; instead, the reaction afforded a complex mixture of products. These results indicate that, despite their structural analogy to germylenes and stannylenes, the cyclic systems are considerably more robust and do not readily undergo the typical bond activation processes associated with low-valent group 14 species under the tested conditions.

## Experimental Section

### General Procedures

All the experiments were performed under an inert atmosphere. The solvent used was dried using column solvent purification system.^28^ (Me_2_Si)_2_Cl_2_, PhMe_2_SiCl, Me_3_SiCl, Me_3_SiI, Me_3_SiOTf, were used without further purification. The deuterated solvent was dried to measure the air-sensitive samples (C_6_D_6_ was dried by 24 h reflux above a sodium/potassium alloy). ^1^H and ^13^C NMR spectra were recorded either on a Varian INOVA 300 MHz, a Bruker AVANCE DPX 200 MHz, or a 400 MHz spectrometer Jeol JNM-ECZL with Royal HFX-Probes with auto sampler in C_6_D_6_ and referenced vs TMS using the internal ^2^H-lock signal of the solvent. Compounds **1** and **13** were synthesized following the reported protocol.^1,15^ UV/vis spectra were recorded with an Agilent Cary 60 UV/vis spectrometer. IR spectra were recorded with a Bruker α. Melting points were determined by a Stuart automatic melting point (SMP50). Elemental analyses were carried out on a Hanau Vario Elementar EL apparatus. Ball milling was done with Retsch MM 400 instrument.

### Safety Statements

All reactions involving germanium and tin halides, acylgermanes, germenolates, and bisgermenolates were carried out under an inert atmosphere using standard Schlenk or glovebox techniques, due to their sensitivity toward moisture and air. Mechanochemical reactions (ball-milling) were performed in sealed jars to prevent exposure to reactive dusts or atmospheric moisture. Proper shielding was used to avoid mechanical hazards from milling equipment. Chlorogermanes, chlorostannanes, and halosilanes are corrosive and may release toxic gases upon contact with moisture. They were handled in a fume hood with appropriate personal protective equipment (lab coat, nitrile gloves, safety goggles).

UV–vis irradiation experiments and photo-CIDNP measurements were performed using enclosed light sources to avoid accidental UV exposure.

### Method A

A 100 mL Schlenk flask was charged with equimolar amounts of compound **1** and the corresponding halosilanes. Subsequently 20 mL of benzene was added to the reaction mixture and kept on stirring at room temperature for 3–4 h. The orange coloration of the enolate was changed to yellow colored solution. The yellow-colored solution was filtered off and then the solvent was evaporated under vacuum. The yellow oily residue was dissolved in acetonitrile and recrystallized at −30 °C.

### Method B

Compound **1** and the corresponding electrophiles were filled in a 10 mL stainless-steel jar with one stainless-steel 12 mm grinding ball. After ball milling for 1 h at 30 Hz, benzene was added to the yellow-colored mixture and filtered using a syringe filter. Then the solvent was evaporated under a vacuum and yellow oily product was obtained. The yellow oily residue was dissolved in acetonitrile and recrystallized at −30 °C.

### Synthesis of 2

**2** was prepared following method **A** using 0.3 g (0.54 mmol) of compound **1** and 0.076 mL (0.54 mmol; 1 equiv) of TMSI in benzene. Yield: 51% (0.15 g) yellow crystals. ^1^H NMR data (C_6_D_6_, TMS, ppm): 1.97 (s, 9H, Mes–C*H*_3_), 2.24 (s, 18H, Mes–C*H*_3_), 6.46 (s, 6H, Mes-*H*). ^13^C NMR data (C_6_D_6_, TMS, ppm): 227 (C = O) 140.11, 139.99, 133.35, 129.08 (Aryl-*C*), 21.08, 19.98 (Aryl-*C*H_3_). UV–vis: λ [nm] (ε [L mol^–1^ cm^–1^]) = 381 (1657), 403 (1228). IR (neat) ν­(C = O): 1641. Anal. Calcd for C_30_H_33_GeIO_3_: C, 56.20; H, 5.19%. Found: C, 56.42; H, 5.30%. MP: 122–127 °C.

### Synthesis of 3

**3** was prepared following method **B** using 0.2 g (0.33 mmol) of compound **1** and 0.051 mL of PhMe_2_SiCl (0.33 mmol). Yield: 92% (0.22 g) of analytically pure yellow oil. ^1^H NMR (C_6_D_6_, TMS, ppm): δ 0.58 (s, 6H, C*H*_3_), 2.00 (s, 9H, Mes–C*H*_3_), 2.10 (s, 18H, Mes–C*H*_3_), 6.41 (s, 6H, Aryl-*H*), 7.49–7.47 and 7.12–7.10 (m, 5H, Ph-*H*). ^13^C NMR (C_6_D_6_, TMS, ppm): δ 237.82 (*C*=O), 143.99, 138.68, 136.77, 135.17, 132.89, 129.64, 128.97 (Aryl-*C*), 21.06, 19.58 (Aryl-*C*H_3_), −1.54 (*C*H_3_). ^29^Si NMR (C_6_D_6_, TMS, ppm) −8.50 (SiPhMe_2_). UV–vis: λ [nm] (ε [L mol^–1^ cm^–1^]) = 383 (887), 402 (742). IR (neat) ν­(C = O): 1478, Anal. Calcd for C_38_H_44_GeO_3_Si: C, 70.27; H, 6.83%. Found: C, 70.20; H, 6.79%.

### Synthesis of 4

Compound **4** was prepared as yellow oily compound following methods **B** with 0.2 g (0.33 mmol) of compound **1** and 0.08 mL (0.66 mmol) of TMSCl or 0.076 mL (0.54 mmol; 1 equiv) of TMSI or 0.056 mL (0.33 mmol) of Me_3_SiOTf. ^1^H NMR data (C_6_D_6_, TMS, ppm): 0.28 (s, 9H, SiMe_3_-*H*), 2.03 (s, 9H, Mes–C*H*_3_), 2.19 (s, 18H, Mes–C*H*_3_), 6.48 (br, 6H, Mes-*H*). ^13^C NMR data (C_6_D_6_, TMS, ppm): 238.27 (*C*=O), 144.17, 138.64, 132.62, 128.99 (Aryl-*C*), 21.07, 19.51 (Aryl-*C*H_3_), 0.14 (Si­(*C*H)_3_). ^29^Si NMR data (C_6_D_6_, TMS, ppm): −3.42 (*Si*Me_3_). Compound is not stable. Consequently, no further characterization was possible.

### Synthesis of 5

**5** was prepared following both methods **A** and **B** with 0.2 g (0.33 mmol) of compound **1** and 0.06 mL of (Me_2_Si)_2_Cl_2_ (0.33 mmol; 1 equiv). Yield: 86% (180 mg) of pure yellow crystalline solid. ^1^H NMR (C_6_D_6_, TMS, ppm): δ 0.52 (s, 6H, CH_3_), 0.76, (s, 6H, CH_3_) 2.00 (s, 9H, Mes–C*H*_3_), 2.10 (s, 18H, Mes–C*H*_3_), 6.45 (s, 6H, Aryl-*H*). ^13^C NMR (C_6_D_6_, TMS, ppm): δ 237.25 (*C*=O), 143.37, 139.06, 132.97, 129.09 (Aryl-*C*), 21.02, 19.72 (Aryl-*C*H_3_), 3.40 (Ge–Si-(*C*H_3_)_2_), −4.17 ppm (Cl–Si-(*C*H_3_)_2_). ^29^Si (C_6_D_6_, TMS, ppm) δ −29.16 (Ge-*Si*-(CH_3_)_2_), 27.90 (Cl-*Si*-(CH_3_)_2_). UV–vis: λ [nm] (ε [L mol^–1^ cm^–1^]) = 381 (1357), 401 (1009). IR (neat) ν­(C = O): 1606, 1641, 1685. Anal. Calcd for C_25_H_34_ClGeO_4_Si_2_: C, 61.32; H, 6.81%. Found: C, 60.72; H, 6.52%. MP: 97 °C.

### Synthesis of 6

**6** was prepared as yellow oily compound following the method **B** with 0.2 g (0.33 mmol) of compound **1** and 80 mg (0.33 mmol,1 equiv) of (Me_2_Si)_3_Cl_2_. ^13^C NMR data (C_6_D_6_, TMS, ppm): 237.67 (*C*=O), 143.47, 138.93, 133.03, 129.08 (Aryl-*C*), 21.07, 19.84 (Aryl-*C*H_3_), 3.50, −1.89, −5.69 (Si-(CH_3_)_2_). ^29^Si NMR data (C_6_D_6_, TMS, ppm): −37.31, −25.16, 27.85. In this case we have observed the formation of some siloxanes as side products which are visible in all the NMR spectra.

### Synthesis of 7

**7** was prepared following method **B** with 0.2 g (0.33 mmol) of compound **1** and 0.05 g (0.165 mmol, 0.5 equiv) of (Me_2_Si)_4_Cl_2_. Yield: 49% (96 mg) of pure yellow solid. ^1^H NMR data (C_6_D_6_, TMS, ppm): 0.60 (d, 24H, Si-(C*H*_3_)_2_). 2.19 (s, 36H Mes–C*H*_3_), 2.03 (s, 18H, Mes–C*H*_3_), 6.49 (s, 12H, Aryl-*H*). ^13^C NMR data (C_6_D_6_, TMS, ppm): 238.26 (*C*=O), 143.73, 138.79, 133.12, 129.08 (Aryl-*C*), 21.05, 19.94 (Aryl-*C*H_3_), – 1.06 (Si-(*C*H_3_)_2_), −3.59 (Si-(*C*H_3_)_2_). ^29^Si NMR data (C_6_D_6_, TMS, ppm): −32.56. −22.23. UV–vis: λ [nm] (ε [L mol^–1^ cm^–1^]) = 383 (1120), 404 (828). Anal. Calcd for C_68_H_90_Ge_2_O_6_Si_4_: C, 64.77; H, 7.19%. Found: C, 64.00; H, 7.27%.

### Synthesis of 8

**8** was prepared following the method **B** with 0.2 g (0.33 mmol) of compound **1** and 0.021 mL (0.33 mmol, 1 equiv) of Me_2_GeCl_2_. ^1^H NMR data (C_6_D_6_, TMS, ppm): 0.80 (s, 6H, −C*H*_3_), 2.00 (s, 9H, Mes–C*H*_3_), 2.22 (s, 18H, Mes–C*H*_3_), 6.41 (br, 6H, Aryl-*H*). ^13^C NMR data (C_6_D_6_, TMS, ppm): 233.47 (*C*=O), 143.12, 139.48, 132.85, 129,10 (Aryl-*C*), 21.08, 19.60 (Aryl-*C*H_3_), 7.22 (Me-*C*).

### Synthesis of 9

**9** was prepared following method **B** with 0.2 g (0.33 mmol, 1 equiv) of compound **1** and 0.024 mL of GeEt_2_Cl_2_ (0.33 mmol, 1 equiv). Yield: 44% (93 mg) of pure yellow solid. ^1^H NMR data (C_6_D_6_, TMS, ppm): 0.91–1.45 (m, 10H, C_2_ *H*_5_), 2.00 (s, 9H, Mes–C*H*_3_), 2.25 (s, 18H, Mes–C*H*_3_), 6.41­(s, 6H, Aryl-*H*). ^13^C NMR data (C_6_D_6_, TMS, ppm): 234.17 (*C*=O), 143.17, 139.36, 133.00, 129.07 (Aryl-*C*), 21.06, 19.76 (Aryl-*C*H_3_), 15.12, 8.74 (Et-*C*). UV–vis: λ [nm] (ε [L mol^–1^ cm^–1^]) = 381 (537), 401 (389). IR (neat) ν­(C = O): 1605, 1640, 1673. Anal. Calcd for C_34_H_43_ClGe_2_O_3_: C, 60.02; H, 6.37%. Found: C, 58.75; H, 5.97%. MP: 91 °C.

### Synthesis of 10

**10** was prepared following the method **B** with 0.2 g (0.33 mmol, 1 equiv) of compound **1** and 0.034 mL (0.33 mmol, 1 equiv) of Ph_2_GeCl_2_ Yield: 49% (118 mg) of pure yellow solid ^1^H NMR data (C_6_D_6_, TMS, ppm): 1.93, (s, 9H Mes–C*H*_3_), 2.13 (s, 18H, Mes–C*H*_3_), 6.31 (s, 6H, Aryl-*H*), 7.06–7.10 (m, 6H, Ph), 7.95–7.98 (m, 4H, Ph). ^13^C NMR data (C_6_D_6_, TMS, ppm): 232.50 (*C*=O), 142.60, 139.44, 137.77, 134.29, 133.29, 129.95, 129.14, 128.59 (Aryl-*C*), 19.75, 21.04 (Aryl-*C* H_3_). UV–vis: λ [nm] (ε [L mol^–1^ cm^–1^]) = 381 (1190), 400 (884). IR (neat) ν­(C = O): 1606, 1632, 1647. Anal. Calcd for C_42_H_43_ClGe_2_O_3_: C, 64.97; H, 5.58%. Found: C, 62.39; H, 4.96%. MP: 110 °C.

### Synthesis of 11

**11** was prepared following the method **B** with 0.2 g (0.33 mmol) of compound **1** and 0.086 mL (0.33 mmol) of (C_4_H_9_)_3_GeCl.Yield: 43% (100 mg) of pure yellow solid. ^1^H NMR data (C_6_D_6_, TMS, ppm): 0.93–0.97 (m, 9H, *t*Bu–C*H*_3_) 1.18–1.40 (m, 18H, *t*Bu–C*H*_2_), 2.03 (s, 9H, Mes–C*H*_3_), 2.25 (s, 18H, Mes–C*H*_3_), 6.50 (s, 6H, Aryl-*H*). ^13^C NMR data (C_6_D_6_, TMS, ppm): 238.39 (*C*=O), 144.05, 138.69, 132.88, 129.06 (Aryl-*C*) 13.90, 14.91, 27.04, 28.32 (*t*Bu-*C*), 19.74, 21.05 (Aryl-*C*H_3_). UV–vis: λ [nm] (ε [L mol^–1^ cm^–1^]) = 382 (766), 401 (594). IR (neat) ν­(C = O): 1604, 1629, 1649, 1674. Anal. Calcd for C_42_H_60_Ge_2_O_3_: C, 66.53, H, 7.98%; Found: C, 65.17, H, 7.69%. MP: 74 °C

### Synthesis of 12

**12** was prepared following the method **B** with 0.2 g (0.33 mmol) of compound **1** and 0.089 mL (0.33 mmol) of (C_4_H_9_)_3_SnCl. Yield: 39% (98 mg) of pure yellow solid. ^1^H NMR data (C_6_D_6_, TMS, ppm): 0.91–0.95 (m, 9H, *n-*Bu-CH_3_), 1.33–1.35 (m, 18H, *n-*Bu–C*H*_2_), 2.04 (s, 9H, Mes–C*H*_3_), 2.19 (s, 18H, Mes–C*H*_3_), 6.50 (s, 6H, Aryl-*H*). ^13^C NMR data (C_6_D_6_, TMS, ppm): 238.24 (*C*=O), 144.0, 138.74, 132.55, 129.07 (Aryl-*C*), 29.92, 27.87, 13.84, 10.98, (*n-*Bu-*C*), 21.08, 19.65 (Aryl-*C*H_3_), ^119^Sn (C_6_D_6_, TMS, ppm): – 64.71. UV–vis: λ [nm] (ε [L mol^–1^ cm^–1^]) = 382 (1569), 402 (1177). Anal. Calcd for C_42_H_60_GeO_3_Sn: C, 62.72; H, 7.52%. Found: C, 62.24; H, 7.08%. IR­(neat) ν­(C = O): 1641, 1627, 1608. MP: 86 °C

### Synthesis of 14

**14** was prepared following method **B** with 0.2 g (0.45 mmol) of compound **13** and 0.136 g (0.45 mmol) of (Me_2_Si)_4_Cl_2_. ^1^H NMR data (C_6_D_6_, TMS, ppm): 0.39 (s, 12H, Si–C*H*_3_), 0.47 (s, 12H, Si–C*H*_3_), 2.02 (s, 6H, Mes–C*H*_3_), 2.10 (s, 12H Mes–C*H*_3_), 6.33 (s, 4H, Ph). ^13^C NMR data (C_6_D_6_, TMS, ppm): 243.86 (*C*=O), 143.96, 138.10, 132.10, 128.81 (Aryl-*C*), 21.12, 19.66 (Aryl-*C*H_3_), – 3.68, – 6.46 (Si-*C*H_3_) ^29^Si NMR data (C_6_D_6_, TMS, ppm): – 39.3, – 22.6

### Synthesis of 15

**15** was prepared following the method **B** with 0.2 g (0.45 mmol) of compound **13** and 0.044 mL (0.45 mmol) of Et_2_GeCl_2_. Yield: 84% (126 mg) of pure yellow solid. ^1^H NMR data (C_6_D_6_, TMS, ppm): 1.28–1.33 (m, 10H Et–C*H*_3_), 1.47–1.52 (m, 8H, Et–C*H*_2_), 1.99 (s, 12H, Mes–C*H*_3_), 2.28 (s, 24H, Mes–C*H*_3_), 6.50 (s, 8H, Aryl-*H*). ^13^C NMR data (C_6_D_6_, TMS, ppm): 234.59 (*C*=O), 144.28, 139.50, 132.45, 129.26 (Aryl-*C*), 21.00, 19.83 (Aryl-*C*H_3_), 15.58, 8.82 (*C*_2_H_5_). UV–vis: λ [nm] (ε [L mol^–1^ cm^–1^]) = 382 (1027), 400 (885). IR (neat) ν­(C = O): 1605, 1630, 1646. MP: 88 °C.

### Synthesis of 16

**16** was prepared following the method **B** with 0.2 g (0.45 mmol) of compound **13** and 0.09 g (0.45 mmol) of (C_4_H_9_)_2_SnCl_2_. Yield: 56% (100 mg) of pure yellow solid. ^1^H NMR data (C_6_D_6_, TMS, ppm): 0.92–0.96 (m, 12H, *n-*Bu-CH_3_), 1.33–1.56 (m, 24H, *n-*Bu–C*H*_2_), 2.07 (s, 12H, Mes–C*H*_3_), 2.31 (s, 24H, Mes–C*H*_3_), 6.53 (s, 8H, Aryl-*H*). ^13^C NMR data (C_6_D_6_, TMS, ppm): 239.91 (*C*=O), 144.54, 138.39, 131.97, 129.16 (Aryl-*C*), 21.13, 19.98 (Aryl-*C*H_3_), 30.44, 27.88, 14.31, 13.85 (*C*_4_H_9_). UV–vis: λ [nm] (ε [L mol^–1^ cm^–1^]) = 406 (1285). IR (neat) ν­(C = O): 1607, 1630. Anal. Calcd for C_56_H_80_Ge_2_O_4_Sn_2_: C, 56.05; H, 6.72%. Found: C, 56.20; H, 6.43%. MP: 105 °C.

### Synthesis of 17

100 mg of **15** (0.10 mmol) and 25 mg of 1,3-dimethyl-4,5-dimethylimidazol-2-ylidene (IMe_4_) (0.20 mmol; 2.00 equiv) were dissolved in 0.8 mL of C_6_D_6_ in a NMR tube. The reaction solution was brought to 65 °C and stirred for 12 h to ensure complete conversion of the starting material. After removal of the solvent and resuspending the crude product in 1 mL of *n*-pentane, the solution was cooled to −30 °C to ensure precipitation. The germylene **17** was isolated as an orange crystalline solid after filtration. **Yield:** 53 mg (0.10 mmol; 54%) of analytically pure **17** as orange crystalline solid.

## Conclusion

In this work, we have demonstrated the versatile reactivity of triacylgermenolate **1** and geminal bisgermenolate **13** toward a broad range of group 14 halides under mechanochemical conditions. The ball-milling approach proved to be an efficient and selective pathway, affording a variety of new acylgermanes via simple salt metathesis. Only small amounts of benzene were used during the workup and acetonitrile for crystallization, emphasizing the environmentally beneficial and solvent-minimized nature of the mechanochemical protocol. With halosilanes, mono- and disubstituted products were obtained, several of which were structurally characterized by single-crystal X-ray diffraction. However, the resulting silyl-substituted acylgermanes proved to be hydrolytically unstable. In contrast, reactions with chlorogermanes afforded hydrolytically robust Ge–Ge bonded derivatives in moderate yields, many of which were also confirmed crystallographically.

The reactivity of the dianion **13** revealed a distinct behavior: while halosilanes gave unselective mixtures, dichlorogermanes and dichlorostannanes selectively afforded unprecedented four-membered Ge–Ge and Ge–Sn ring systems (**15** and **16**). Notably, compound **15** was found to be stable toward aqueous workup and exhibited photochemical reactivity consistent with α-cleavage, as demonstrated by photo-CIDNP experiments, underscoring its potential as a visible-light photoinitiator.

Overall, these results establish triacylgermenolates and bisgermenolates as valuable synthons for accessing novel acyl metalloids with tunable structural and electronic properties. The combination of mechanochemical synthesis, structural diversity, and photochemical activity highlights their promise as building blocks for next-generation main-group photoinitiators. Future work will focus on harnessing the reactivity of the four-membered Ge- and Sn-containing ring systems as *masked* germylene and stannylene precursors, exploring their potential in bond activation and catalysis. In addition, strategies to enhance the hydrolytic stability of silyl-substituted acylgermanes will be pursued, aiming to expand their applicability as robust visible-light photoinitiators in polymerization chemistry and related photochemical transformations.

## Supplementary Material



## Data Availability

The data that support the findings of this study are available in the Supporting Information of this article. In addition, all underlying NMR, IR and UV/vis data supporting this work are openly available via the TU Graz repository at 10.3217/dgted-hs797.

## Notes

NMR spectra, UV–Vis absorption spectra, and X-ray crystallographic data (PDF)
