# Supplementary material for: Bonding and Reactivity of Germanium Enolates toward Group 14 Halides
Source: Inorg Chem. 2026 Jan 27;65(5):2745–56. doi: 10.1021/acs.inorgchem.5c04482 (PMC12892317; doi:10.1021/acs.inorgchem.5c04482)
Supplement: Supplementary file 1 [file ic5c04482_si_001.pdf]

Supporting Information

Bonding and Reactivity of Germanium Enolates toward Group 14 Halides

Nilanjana Sen,[a] Manfred Drusgala,[a] Roland C. Fischer,[a] Dmytro Neshchadin,[b] Thomas Lainer,[a] Michael Haas[a]*

[a]Institute of Inorganic Chemistry, Graz University of Technology Stremayrgasse 9/IV, 8010 Graz (Austria). Email: michael.haas@tugraz.at

[b]Institute of Physical and Theoretical Chemistry, Graz University of Technology Stremayrgasse 9/II, 8010 Graz (Austria)

Table of Content

[S1]. Comparative study between conventional and ball-mill method **4**

Figure S1. ^{13}C NMR spectrum of **5** in conventional method (above) and ball mill method (below)... 4

[S2]. ^{1}H, ^{13}C, ^{29}Si and ^{119}Sn NMR spectra of compound 2-16. .... **5**

Figure S2. ^{1}H NMR spectrum of compound **3** in C_6D_6 5

Figure S4. ^{29}Si NMR spectrum of compound **3** in C_6D_6. 7

Figure S5. ^{1}H NMR spectrum of compound **2** in C_6D_6. 8

Figure S6. ^{13}C NMR spectrum of compound **2** in C_6D_6 9

Figure S7. ^{1}H NMR spectrum of compound **4** in C_6D_6. 10

Figure S8. ^{13}C NMR spectrum of compound **4** in C_6D_6 11

Figure S9. ^{29}Si NMR spectrum of compound **4** in C_6D_6. 12

Figure S10. ^{1}H NMR spectrum of compound **5** in C_6D_6. 13

Figure S11. ^{13}C NMR spectrum of compound **5** in C_6D_6. 14

Figure S12. ^{29}Si NMR spectrum of compound **5** in C_6D_6 15

Figure S13. ^{1}H NMR spectrum of compound **6** in C_6D_6. ● = Unisolated siloxanes 16

Figure S14. ^{13}C NMR spectrum of compound **6** in C_6D_6. 17

Figure S15. ^{29}Si NMR spectrum of compound **6** in C_6D_6. ● = Unisolated siloxanes 18

Figure S16. ^{1}H NMR spectrum of compound **7** in C_6D_6. 19

Figure S17. ^{13}C NMR spectrum of compound **7** in C_6D_6. 20

Figure S18. ^{29}Si NMR spectrum of compound **7** in C_6D_6. 21

Figure S19. ^{1}H NMR spectrum of compound **8** in C_6D_6. 22

Figure S20. ^{13}C NMR spectrum of compound **8** in C_6D_6 23

Figure S21. ^{1}H NMR spectrum of compound **9** in C_6D_6 24

Figure S22. ^{13}C NMR spectrum of compound **9** in C_6D_6. 25

Figure S23. ^{1}H NMR spectrum of compound **10** in C_6D_6. 26

Figure S24. ^{13}C NMR spectrum of compound **10** in C_6D_6. 27

Figure S25. ^{1}H NMR spectrum of compound **11** in C_6D_6. 28

Figure S26. ^{13}C NMR spectrum of compound **11** in C_6D_6. 29

Figure S27. ^{1}H NMR spectrum of compound **12** in C_6D_6. 30

Figure S28. ^{13}C NMR spectrum of compound **12** in C_6D_6. 31

Figure S29. ^{119}Sn NMR spectrum of compound **12** in C_6D_6. 32

Figure S30. ^{1}H NMR spectrum of compound **14** in C_6D_6. 33

Figure S31. ^{13}C NMR spectrum of compound **14** in C_6D_6. 34

Figure S32. ^{29}Si NMR spectrum of compound **14** in C_6D_6. 35

Figure S33. ^{1}H NMR spectrum of compound **15** in C_6D_6. 36

Figure S34. ^{13}C NMR spectrum of compound **15** in C_6D_6. 37

Figure S35. ^{1}H NMR spectrum of compound **16** in C_6D_6. 38

Figure S36. ^{13}C NMR spectrum of compound **16** in C_6D_6. 39

Figure S37. ^{119}Sn NMR spectrum of compound **16** in C_6D_6. 40

[S3]. ^{13}C and ^{29}Si NMR spectra of silyl substituted acylgermanes after aqueous workup. 41

Figure S38. ^{13}C NMR spectrum of silyl substitute acylgermane and aqueous workup. 41

Figure S39. ^{29}Si NMR spectrum of silyl substitute acylgermane and aqueous workup. 42

Figure S40. Series of ^{1}H spectra to monitor the keto-enol equilibrium and subsequent degradation process. 49

[S4]. UV-Vis absorption spectra 50

Figure S41. UV-Vis absorption spectrum of compound **2** 50

Figure S42. UV-Vis absorption spectrum of compound **3**. 50

Figure S43. UV-Vis absorption spectrum of compound **5** 51

Figure S44. UV-Vis absorption spectrum of compound **7** 51

Figure S45. UV-Vis absorption spectrum of compound **9**. 52

Figure S46. UV-Vis absorption spectrum of compound **10** 52

Figure S47. UV-Vis absorption spectrum of compound **11** 53

Figure S48. UV-Vis absorption spectrum of compound 1**2** 53

Figure S49. UV-Vis absorption spectrum of compound **15**. 54

Figure S50. UV-Vis absorption spectrum of compound **17**. 54

[S5]. X-ray Crystallography 55

Figure S51. ORTEP representation for compound **16**. Thermal ellipsoids are depicted at the 50% probability level. Hydrogen atoms are omitted, and mesityl groups are displayed as wireframes for clarity. Selected bond lengths (Å) of **16** with estimated standard deviations: Sn1-Ge1 2.639(2), Sn-C22 2.41(4), Sn1-C26 2.28(6) Ge1-C1 2.12(2), Ge1- C11 2.03(2). 57

[S1]. Comparative study between conventional and ball-mill method

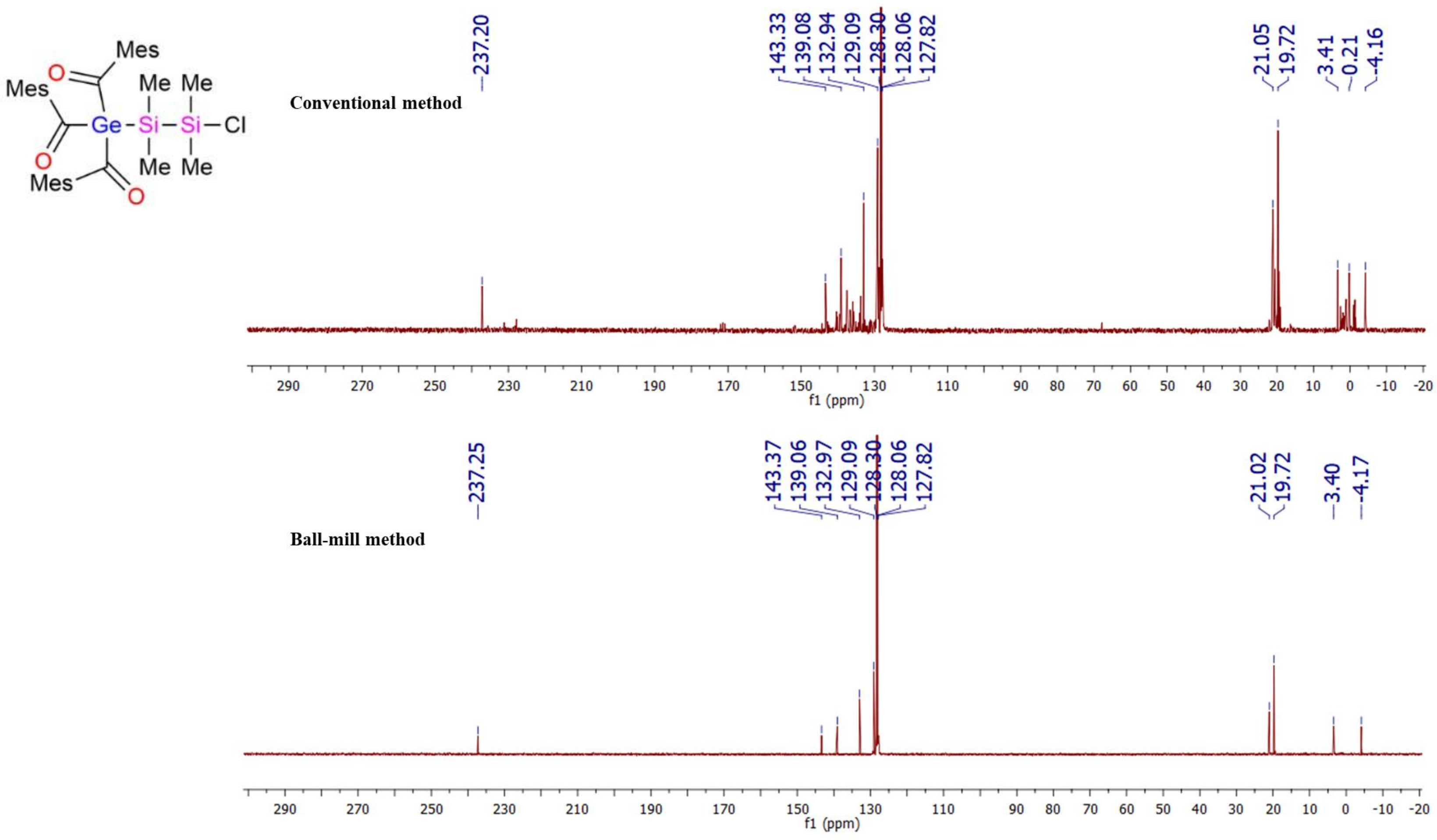

Figure S1. ^{13}C NMR spectrum of **5** in conventional method (above) and ball mill method (below).

[S2]. ^{1}H, ^{13}C, ^{29}Si and ^{119}Sn NMR spectra of compound 2-16.

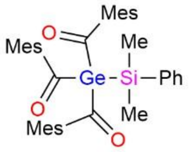

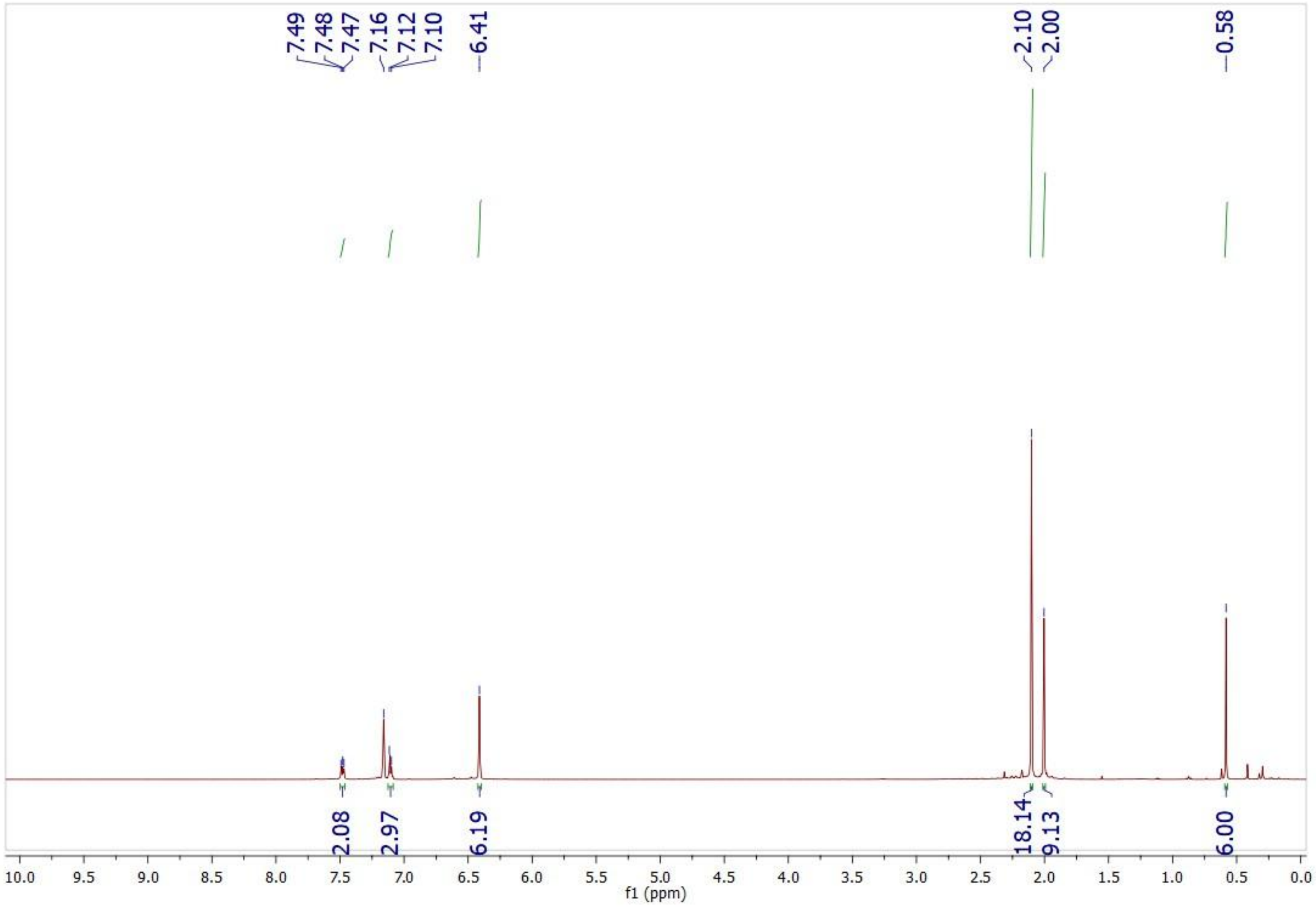

Figure S2. ^{1}H NMR spectrum of compound **3** in C_6D_6.

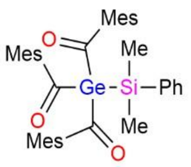

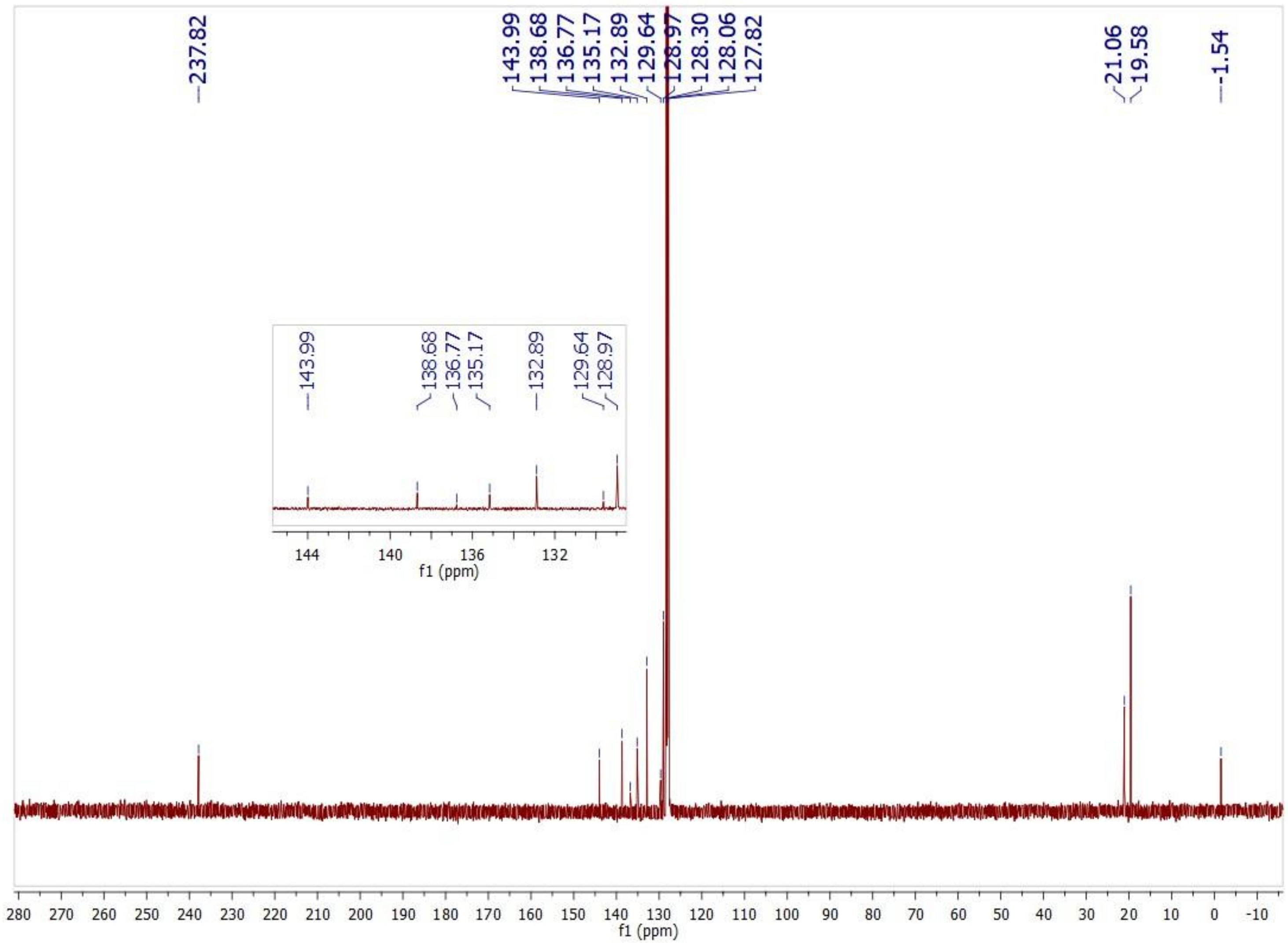

Figure S3. ^{13}C NMR spectrum of compound **3** in C_6D_6.

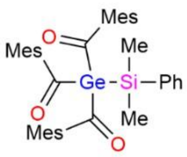

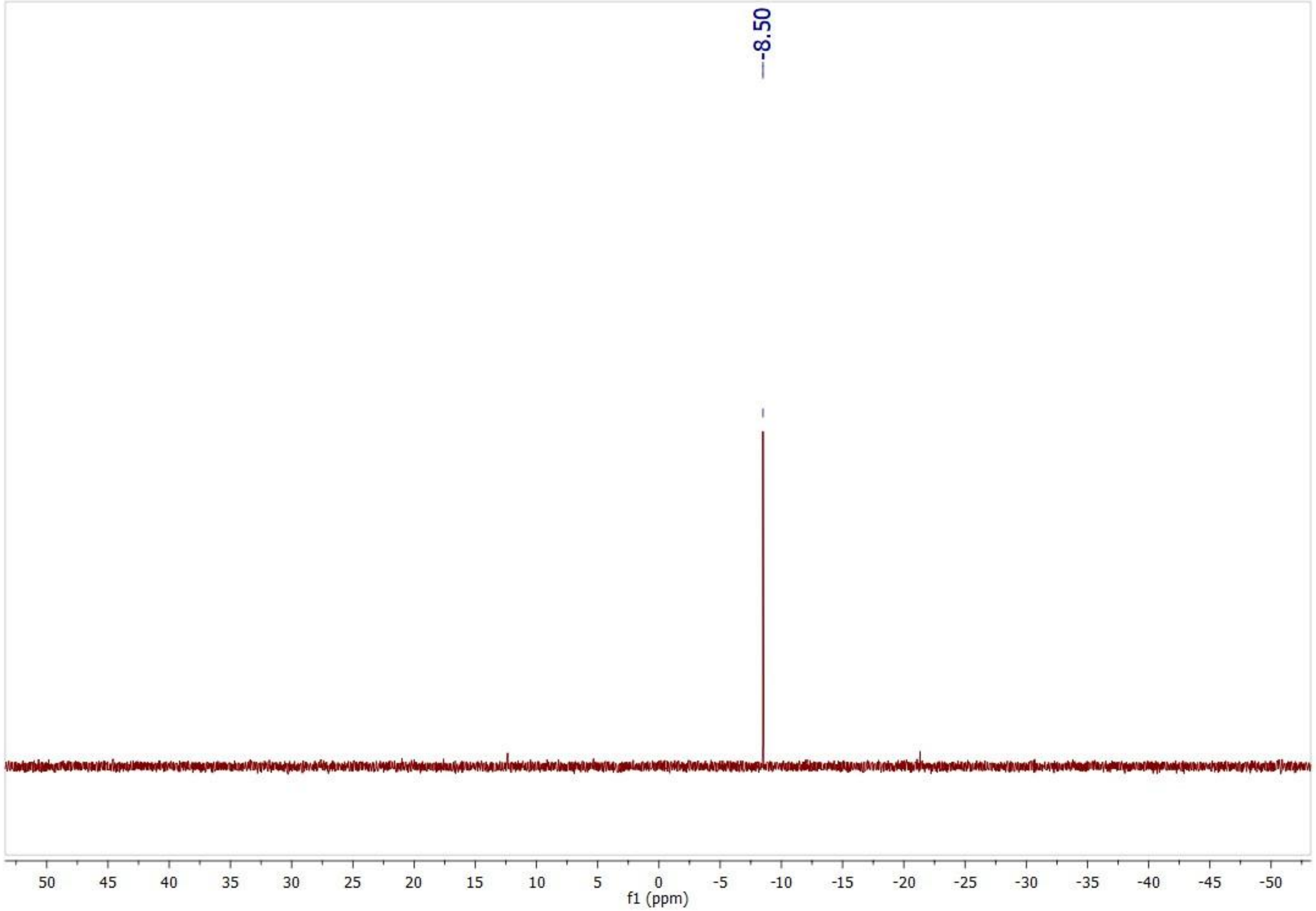

Figure S4. ^{29}Si NMR spectrum of compound **3** in C_6D_6.

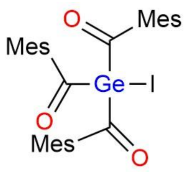

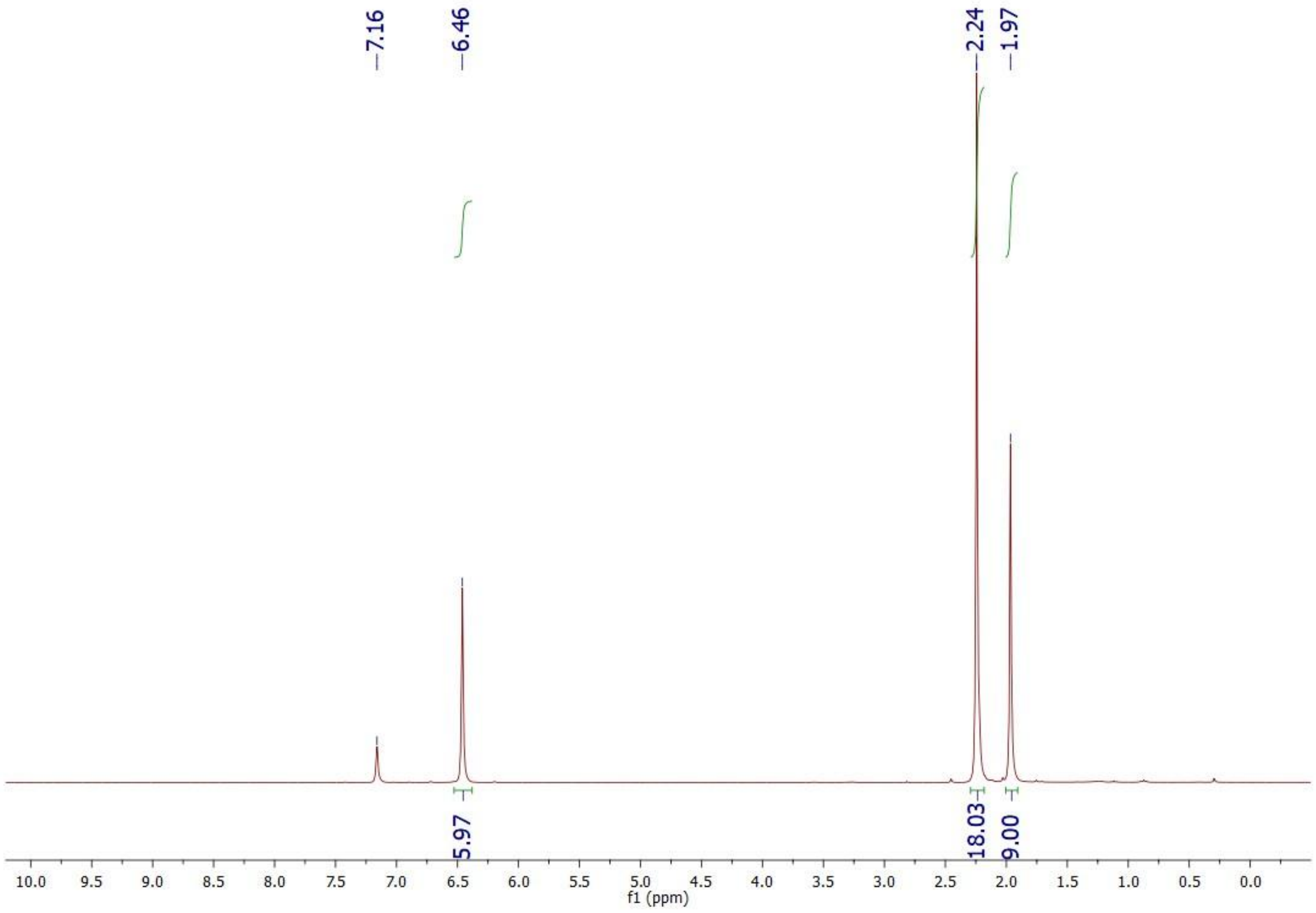

Figure S5. ^{1}H NMR spectrum of compound **2** in C_6D_6.

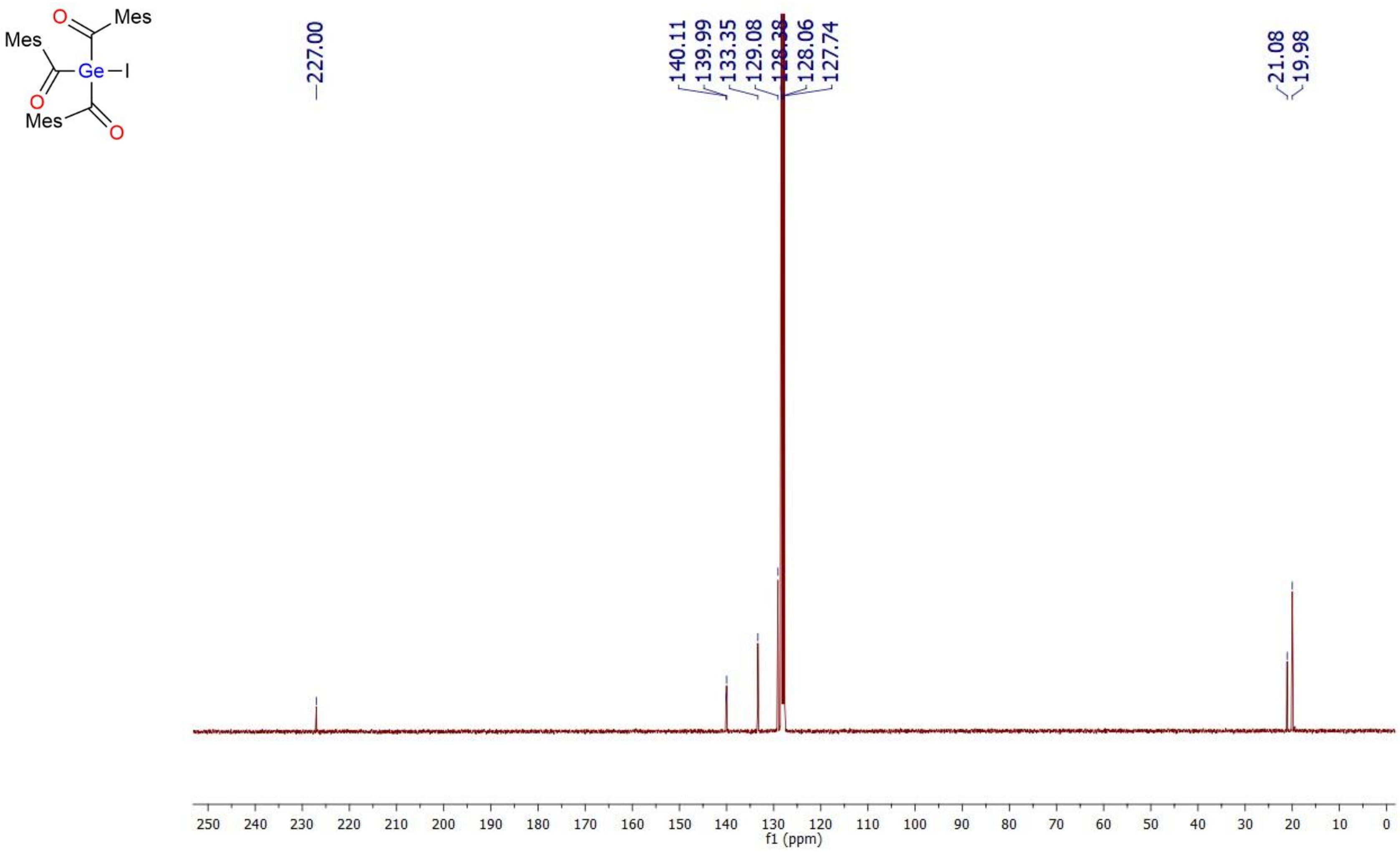

Figure S6. ^{13}C NMR spectrum of compound **2** in C_6D_6.

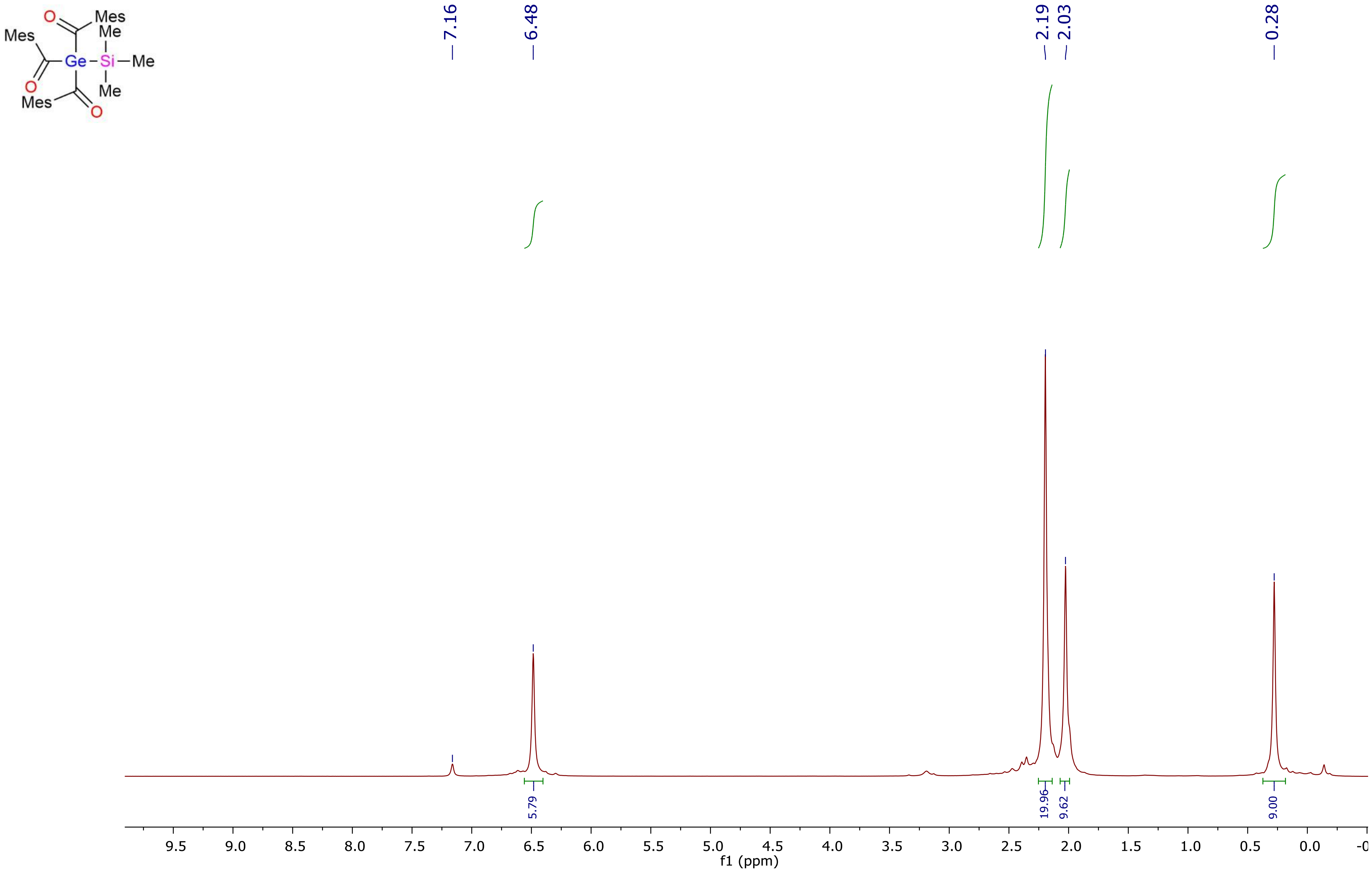

Figure S7. ^{1}H NMR spectrum of compound **4** in C_6D_6.

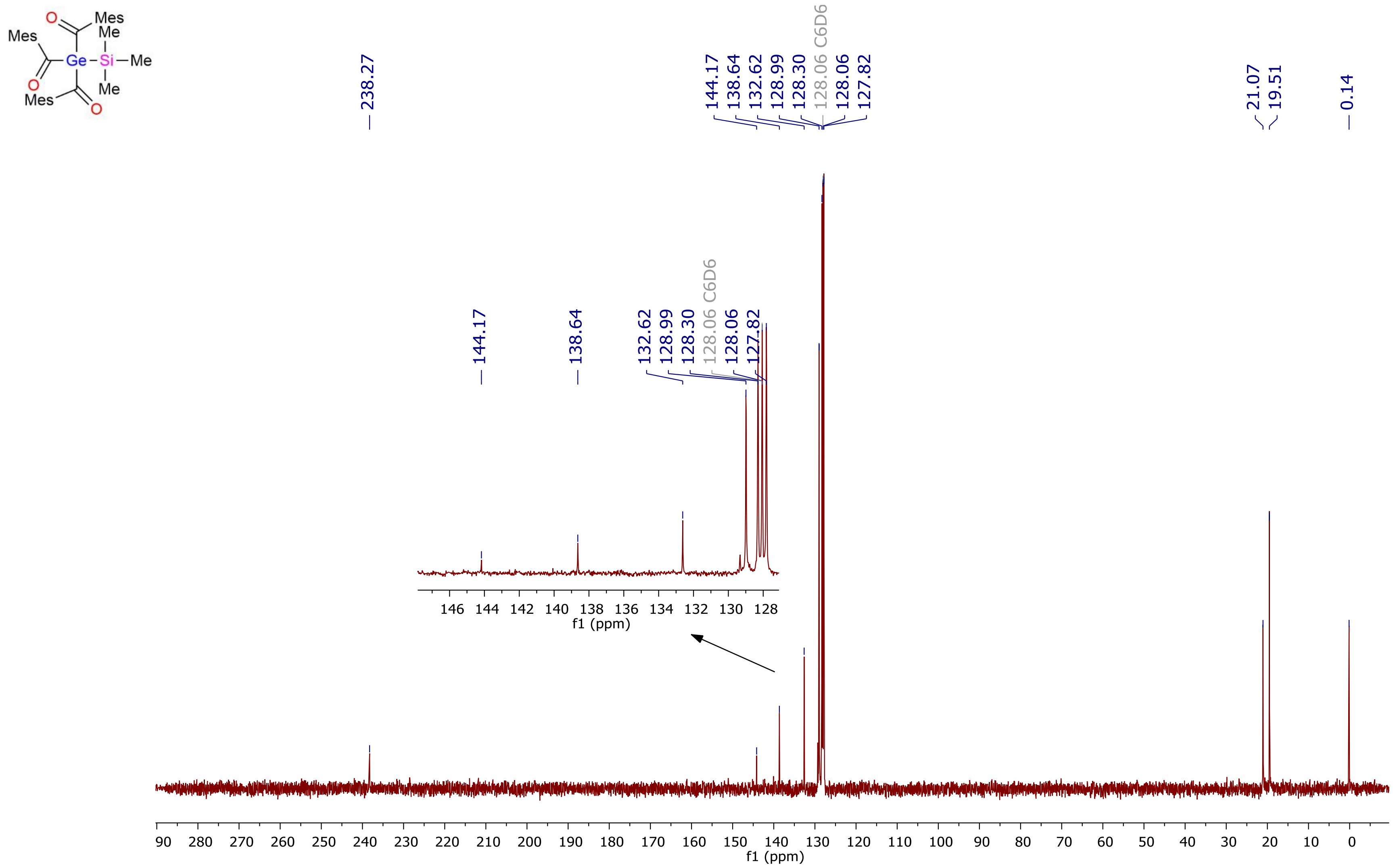

Figure S8. ^{13}C NMR spectrum of compound **4** in C_6D_6.

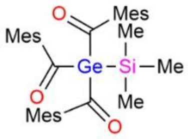

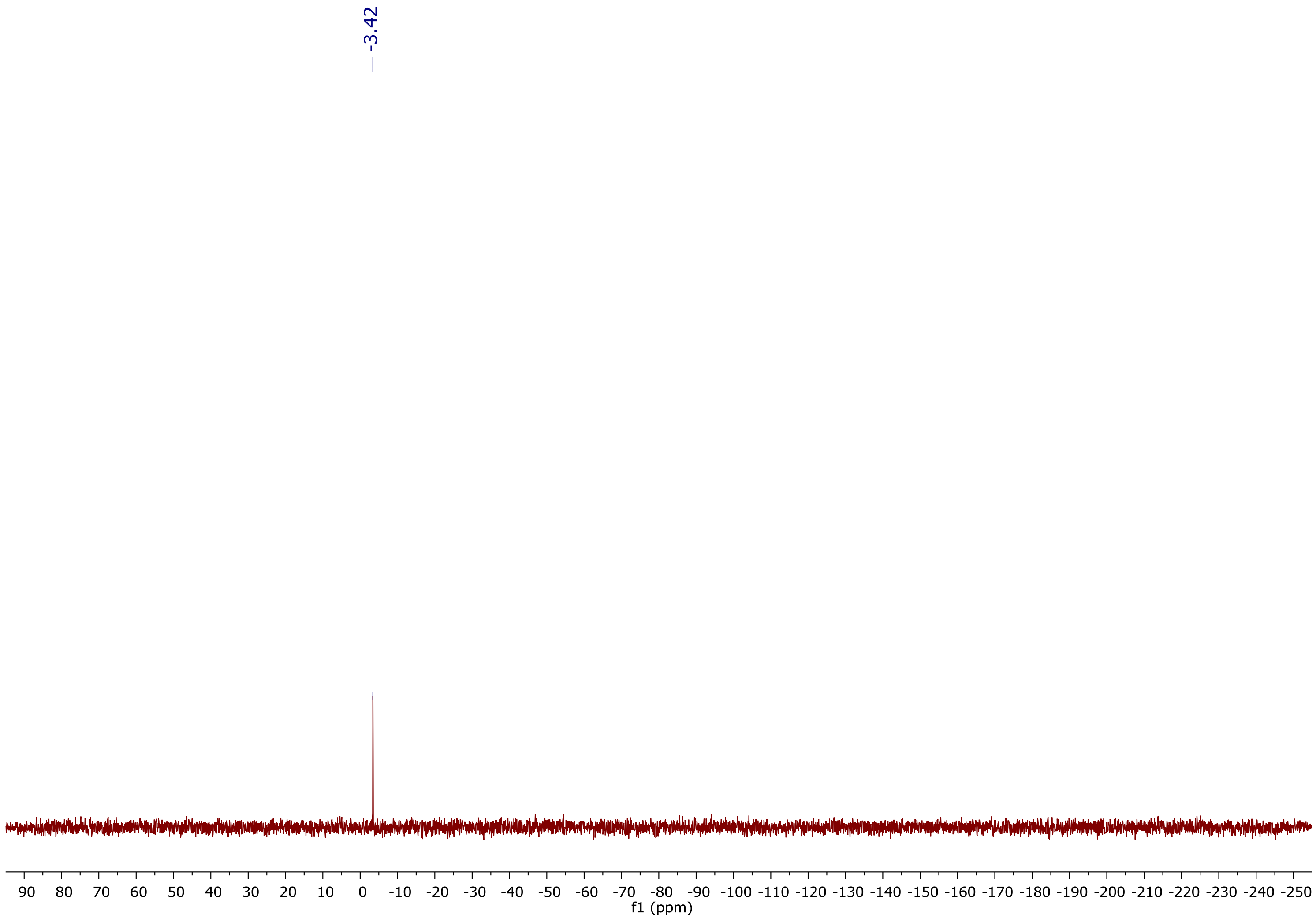

Figure S9. ^{29}Si NMR spectrum of compound **4** in C_6D_6.

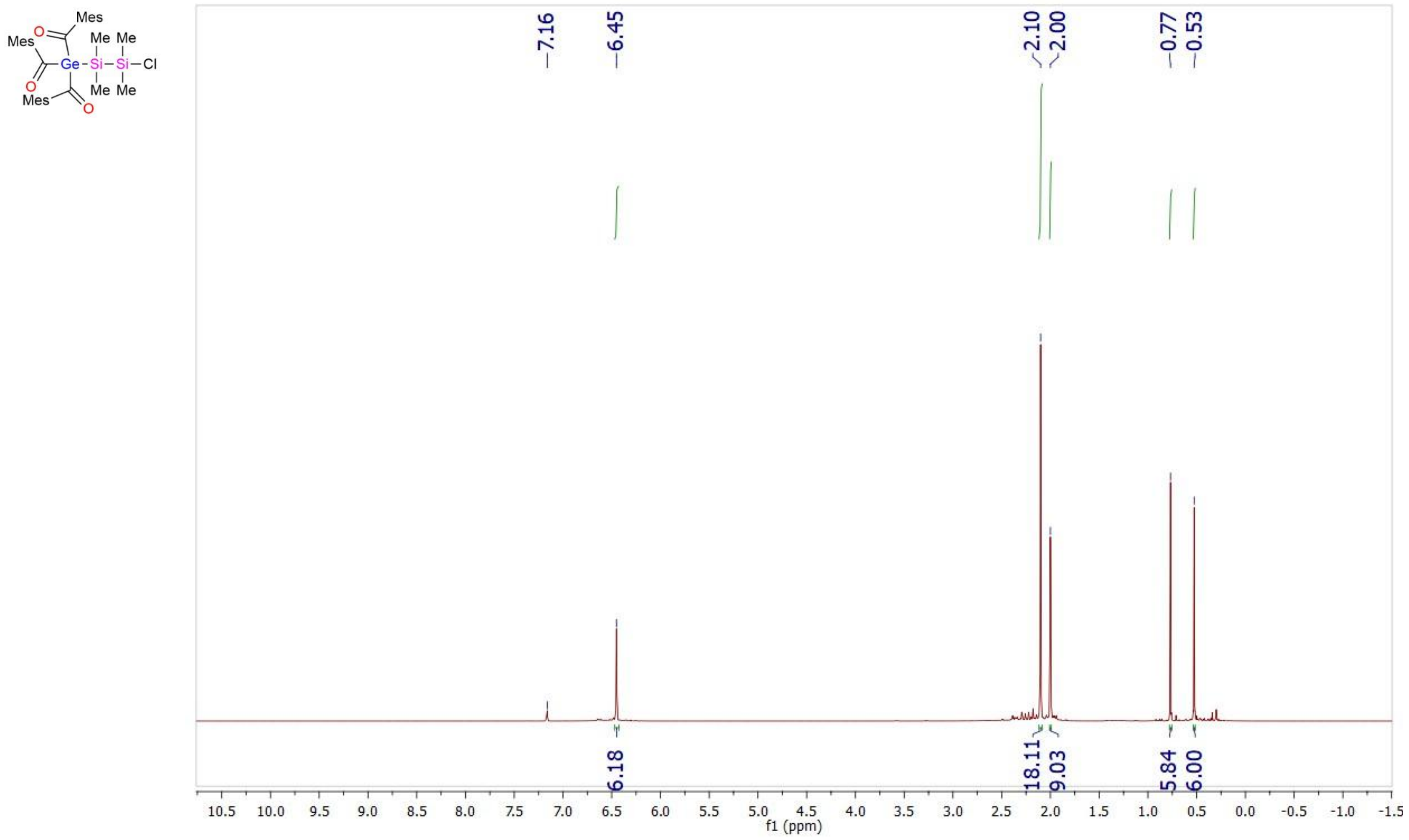

Figure S10. ^{1}H NMR spectrum of compound **5** in C_6D_6.

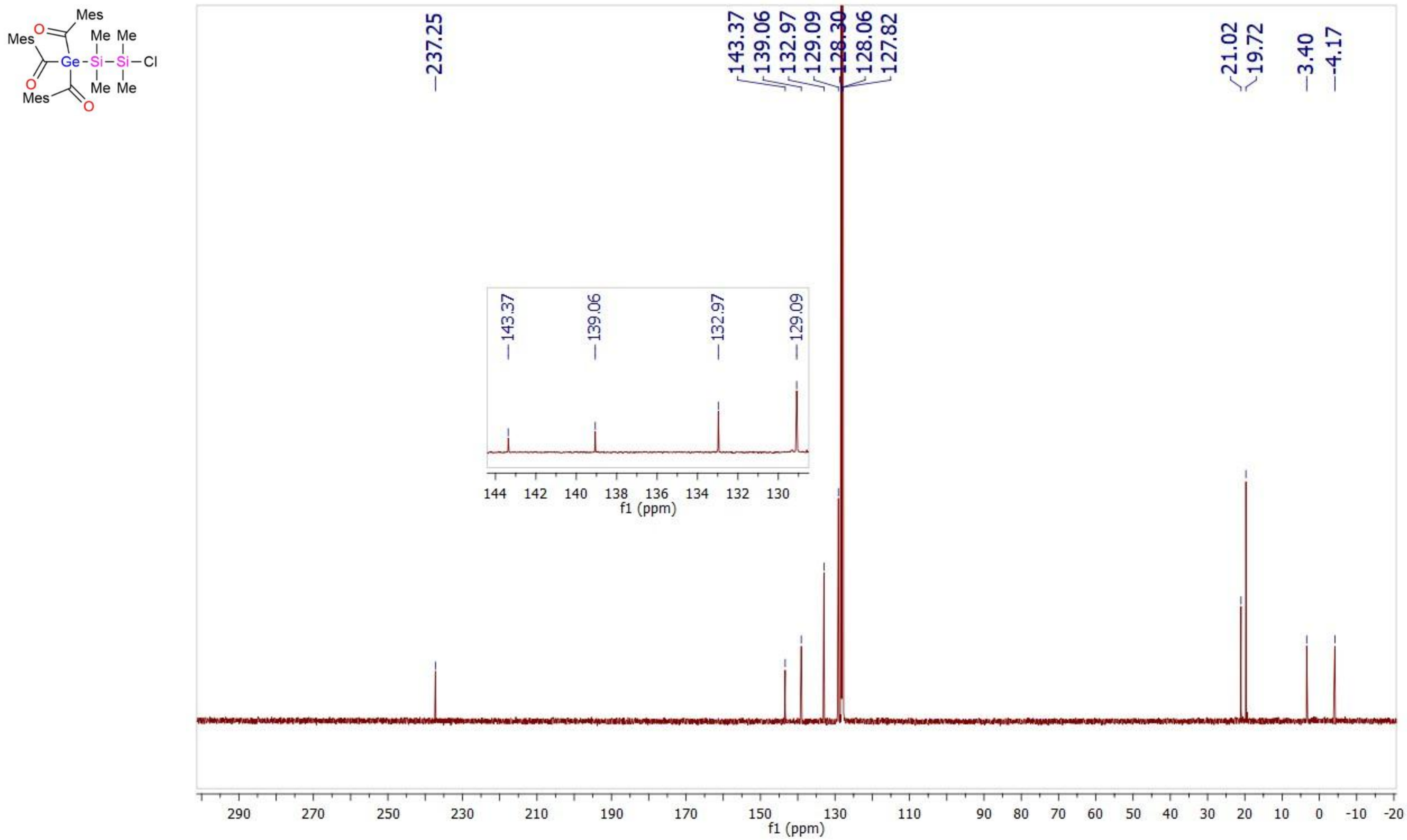

Figure S11. ^{13}C NMR spectrum of compound **5** in C_6D_6.

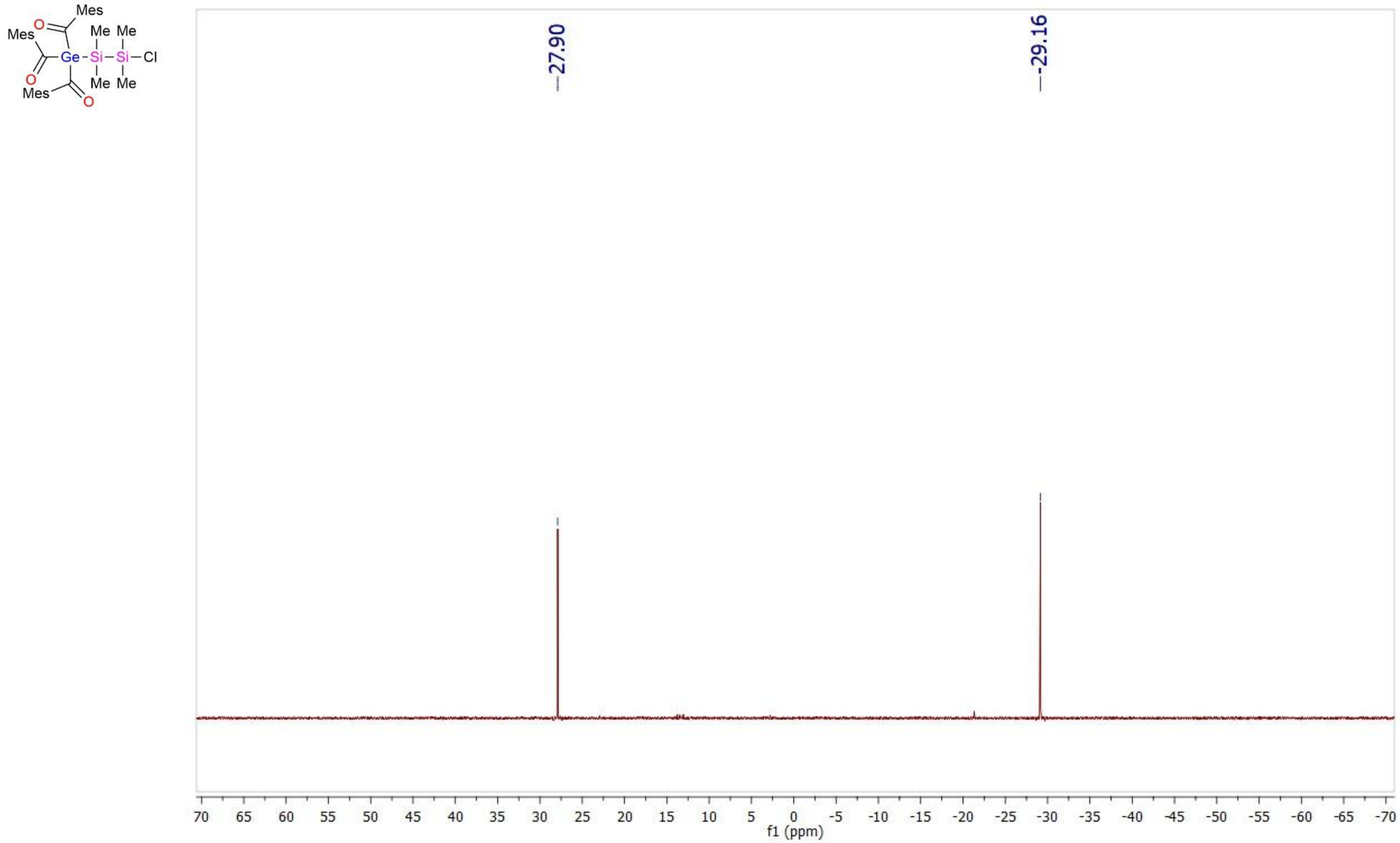

Figure S12. ^{29}Si NMR spectrum of compound **5** in C_6D_6.

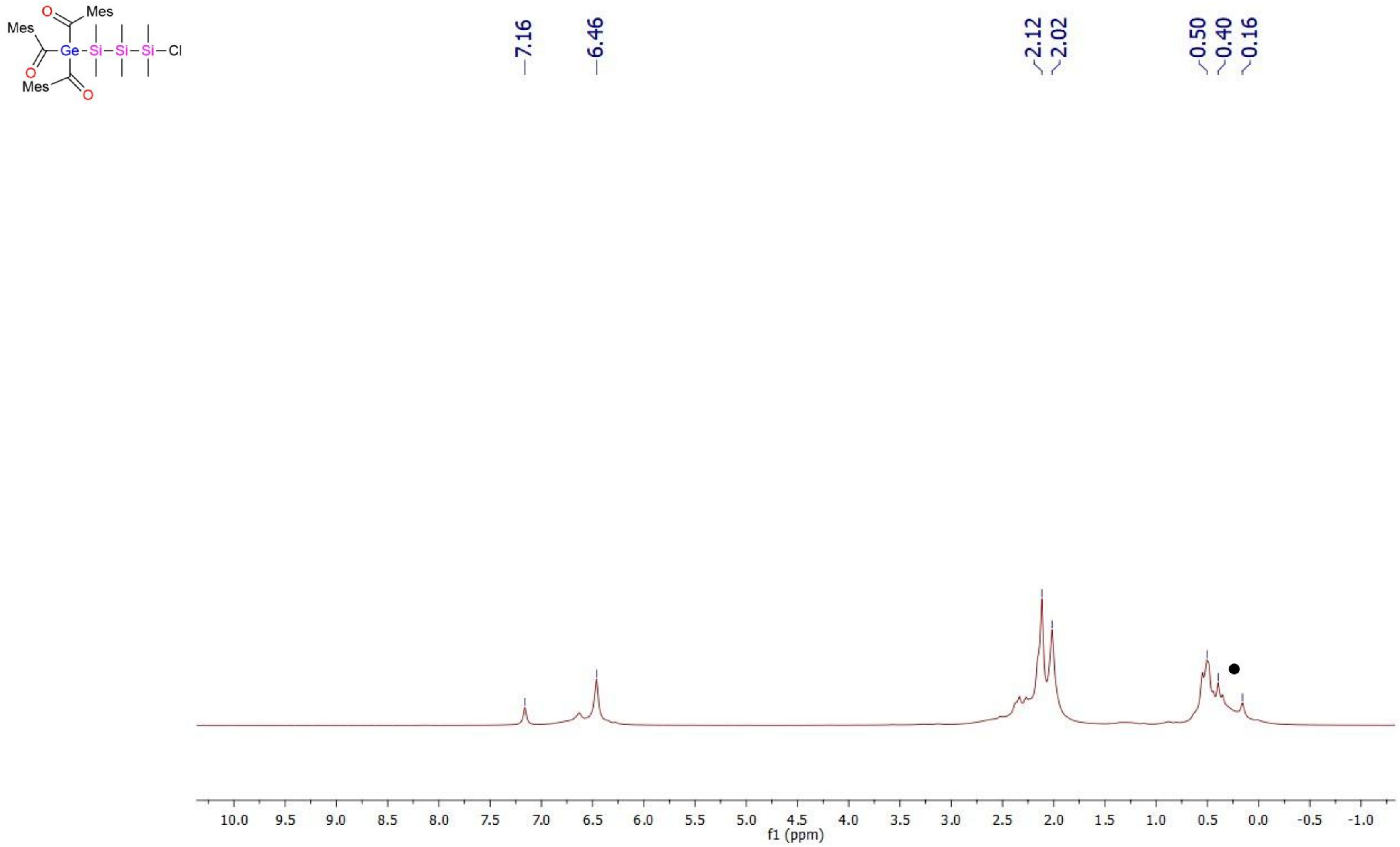

Figure S13. ^{1}H NMR spectrum of compound **6** in C_6D_6. ● = Unisolated siloxanes

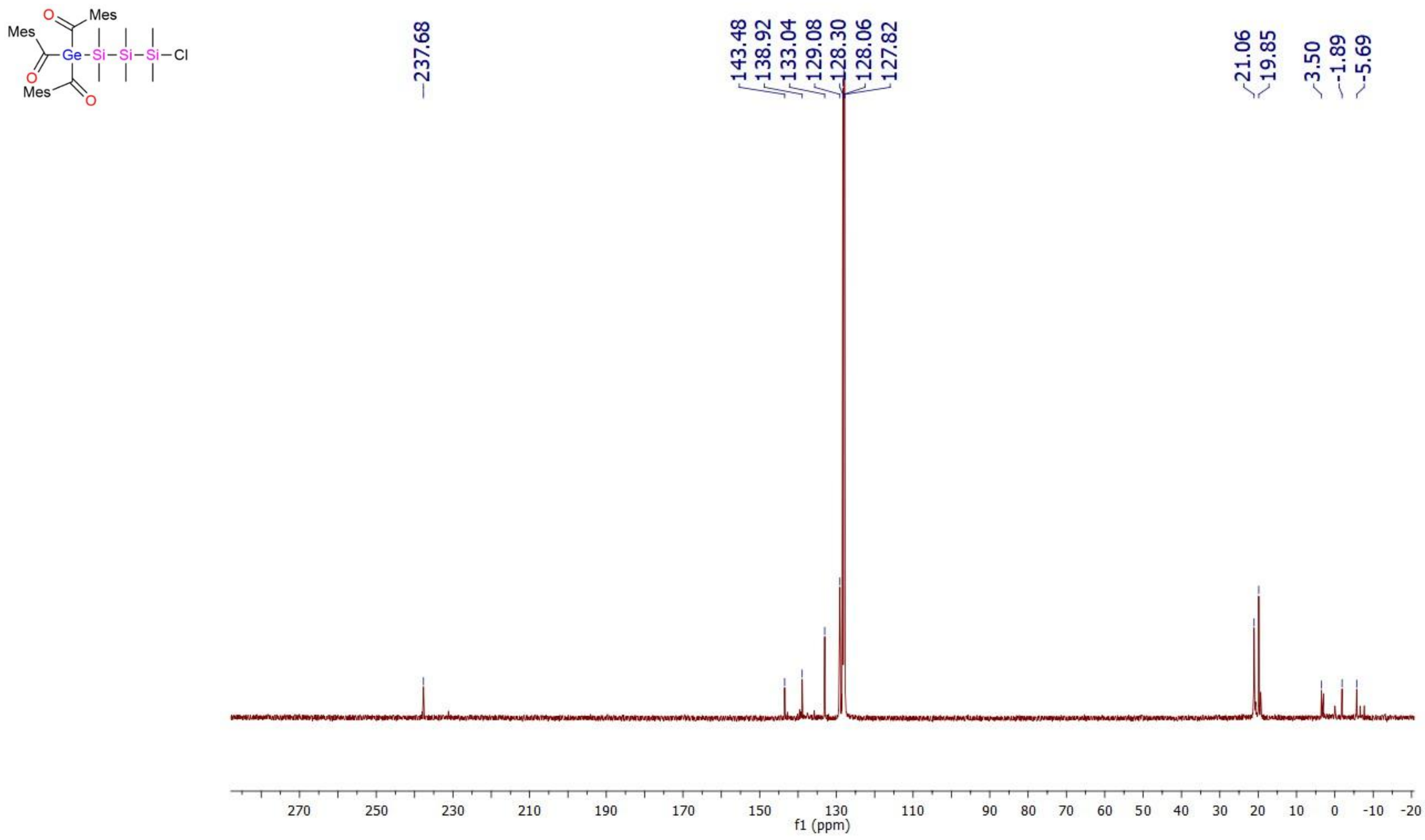

Figure S14. ^{13}C NMR spectrum of compound **6** in C_6D_6.

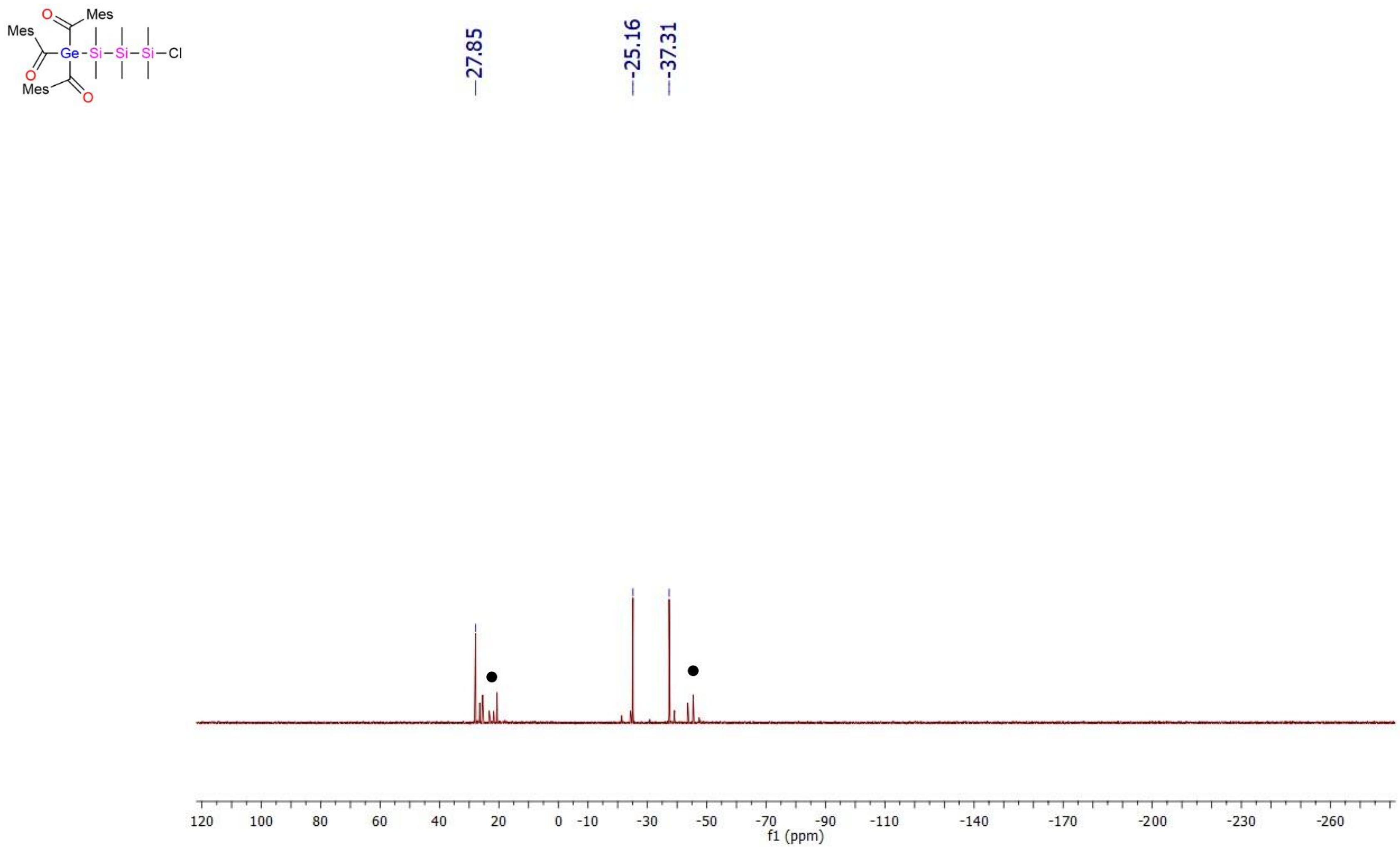

Figure S15. ^{29}Si NMR spectrum of compound **6** in C_6D_6. ● = Unisolated siloxanes

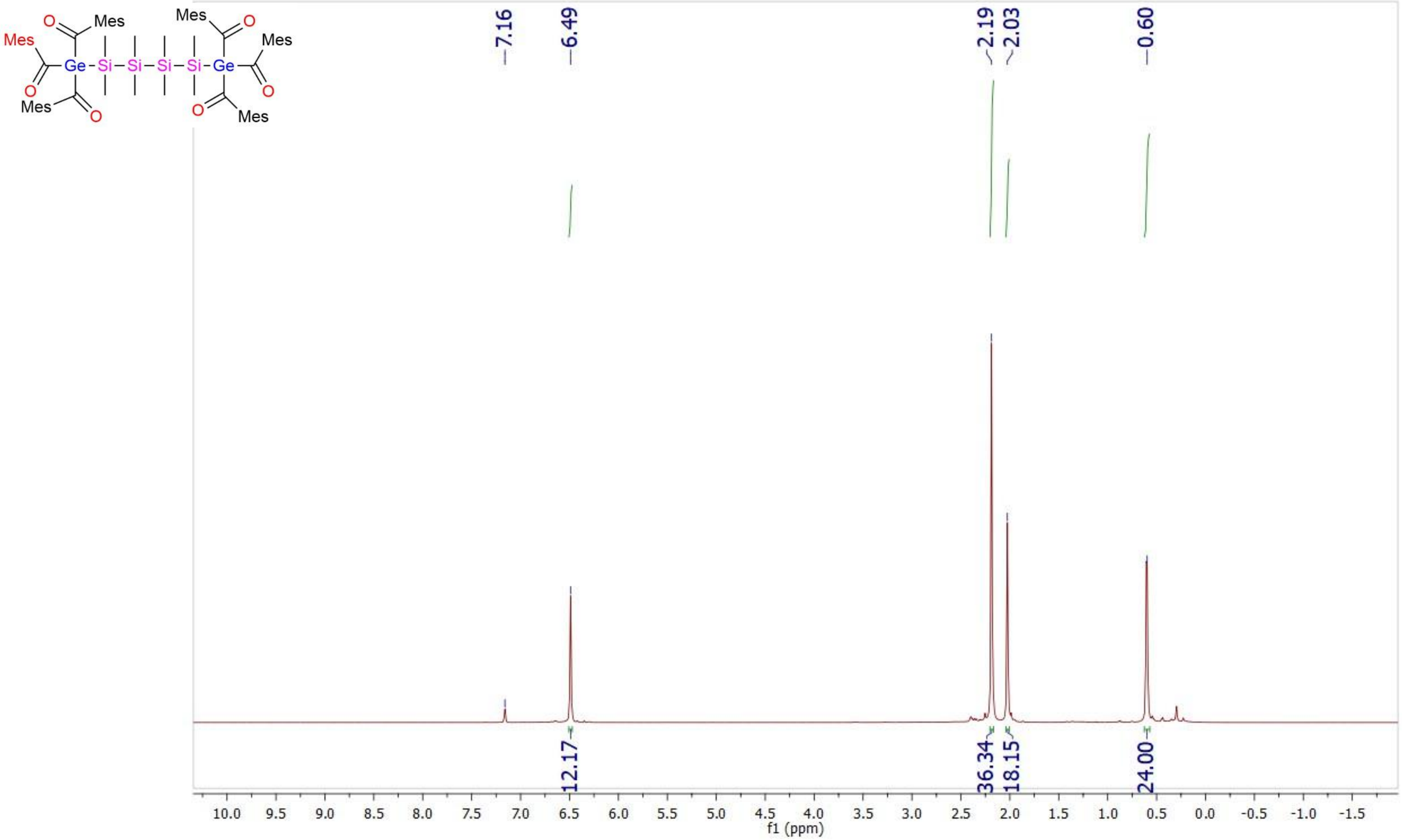

Figure S16. ^{1}H NMR spectrum of compound **7** in C_6D_6.

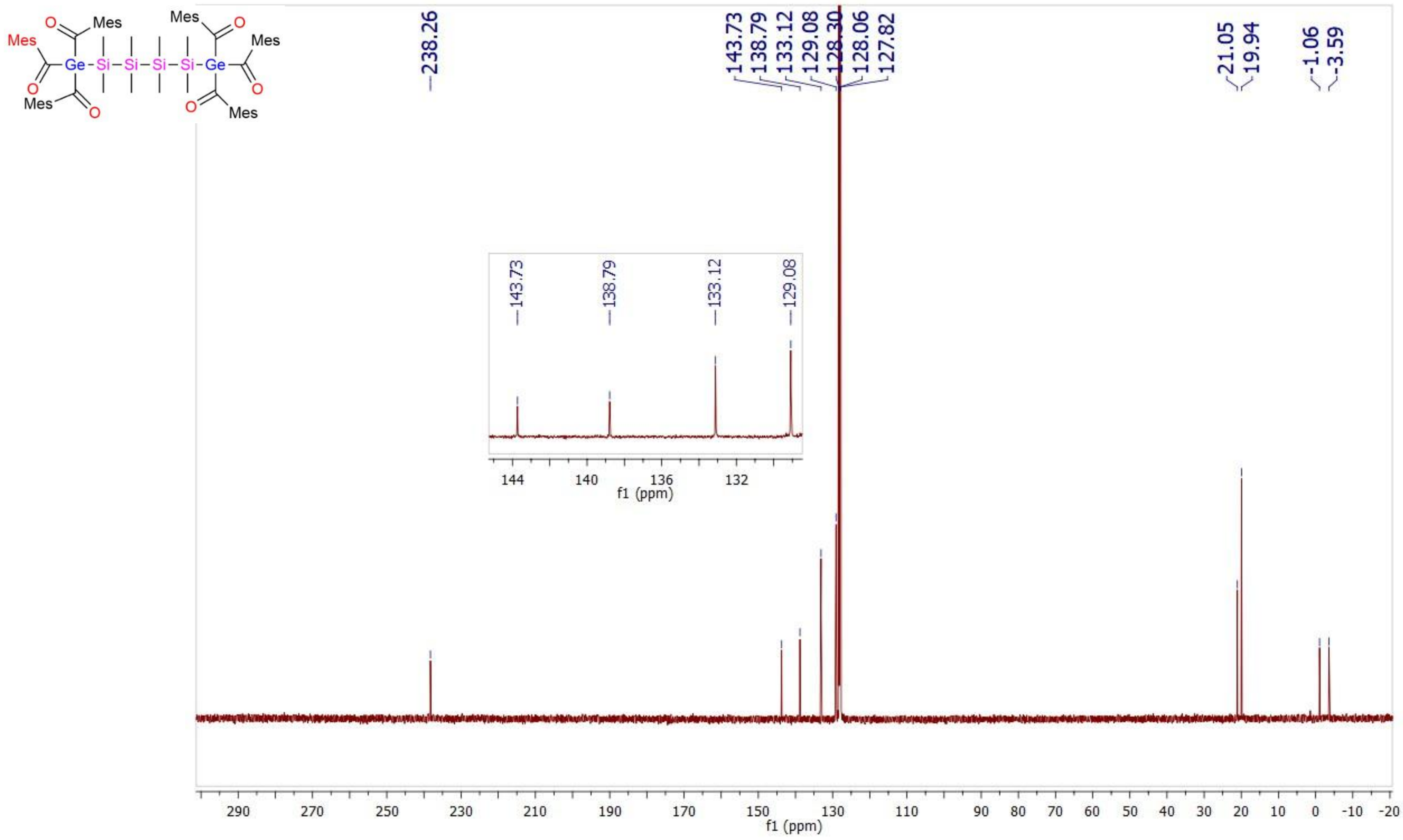

Figure S17. ^{13}C NMR spectrum of compound **7** in C_6D_6.

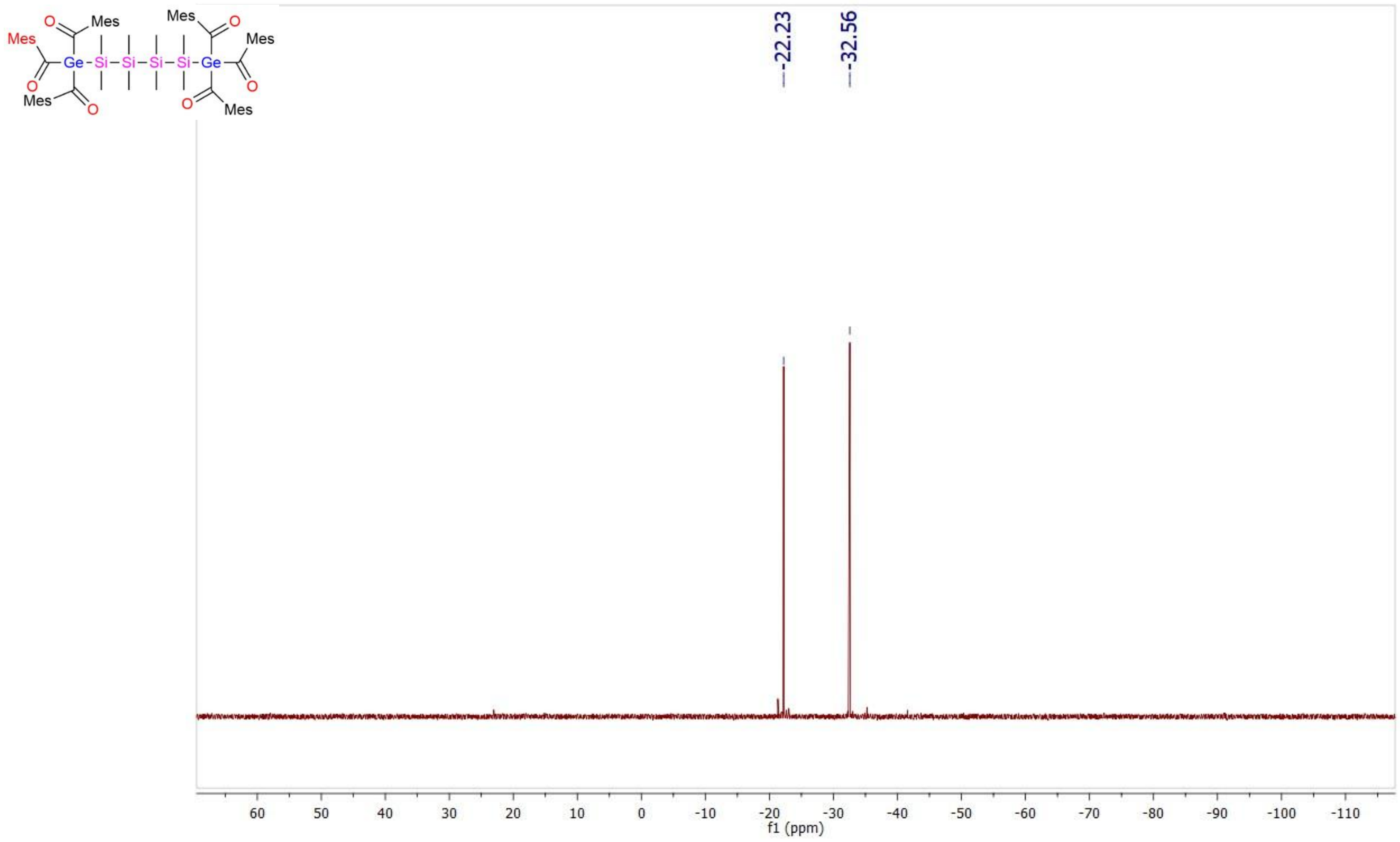

Figure S18. ^{29}Si NMR spectrum of compound **7** in C_6D_6.

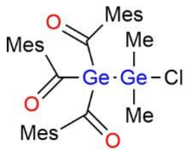

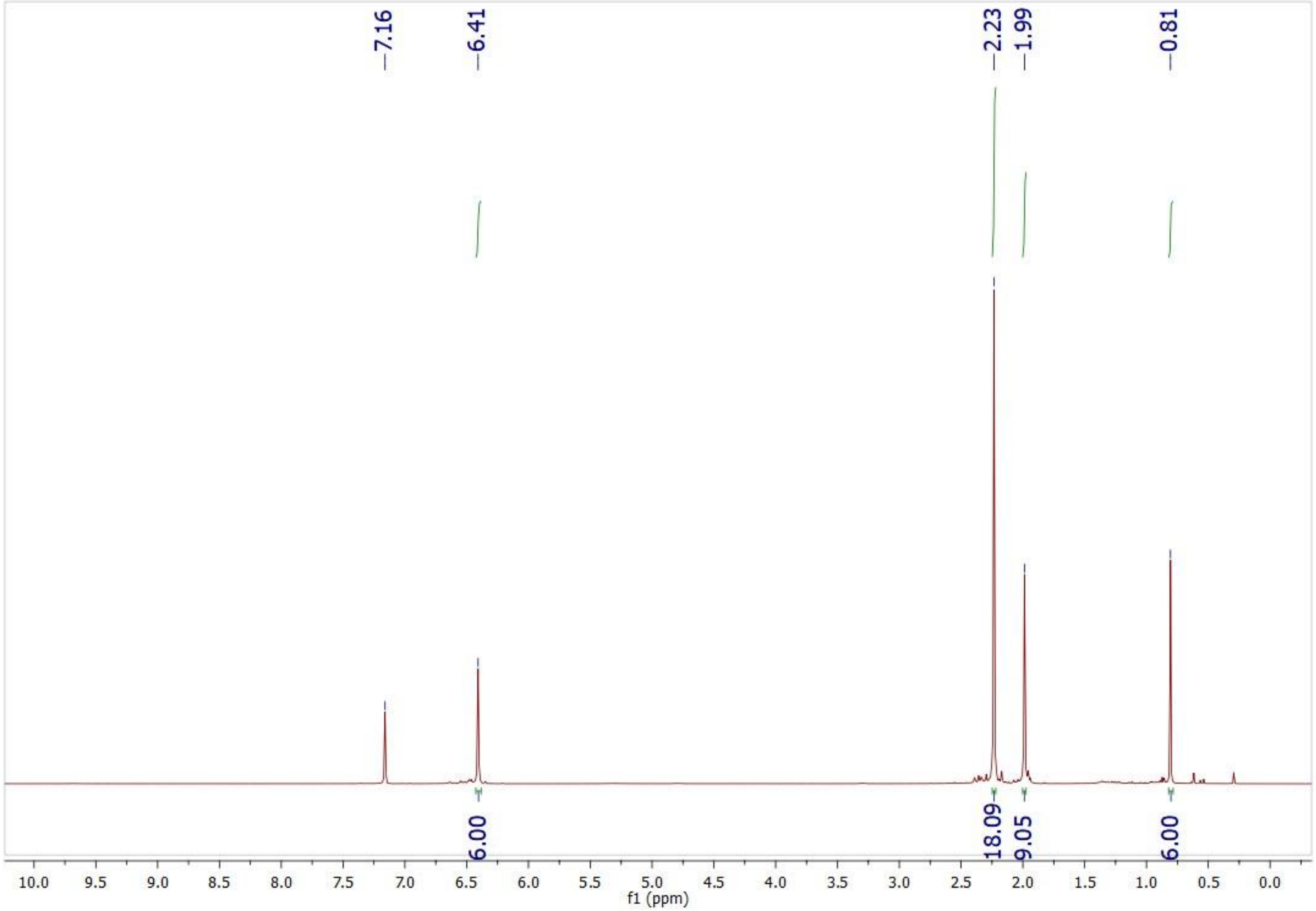

Figure S19. ^{1}H NMR spectrum of compound **8** in C_6D_6.

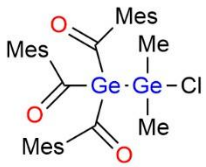

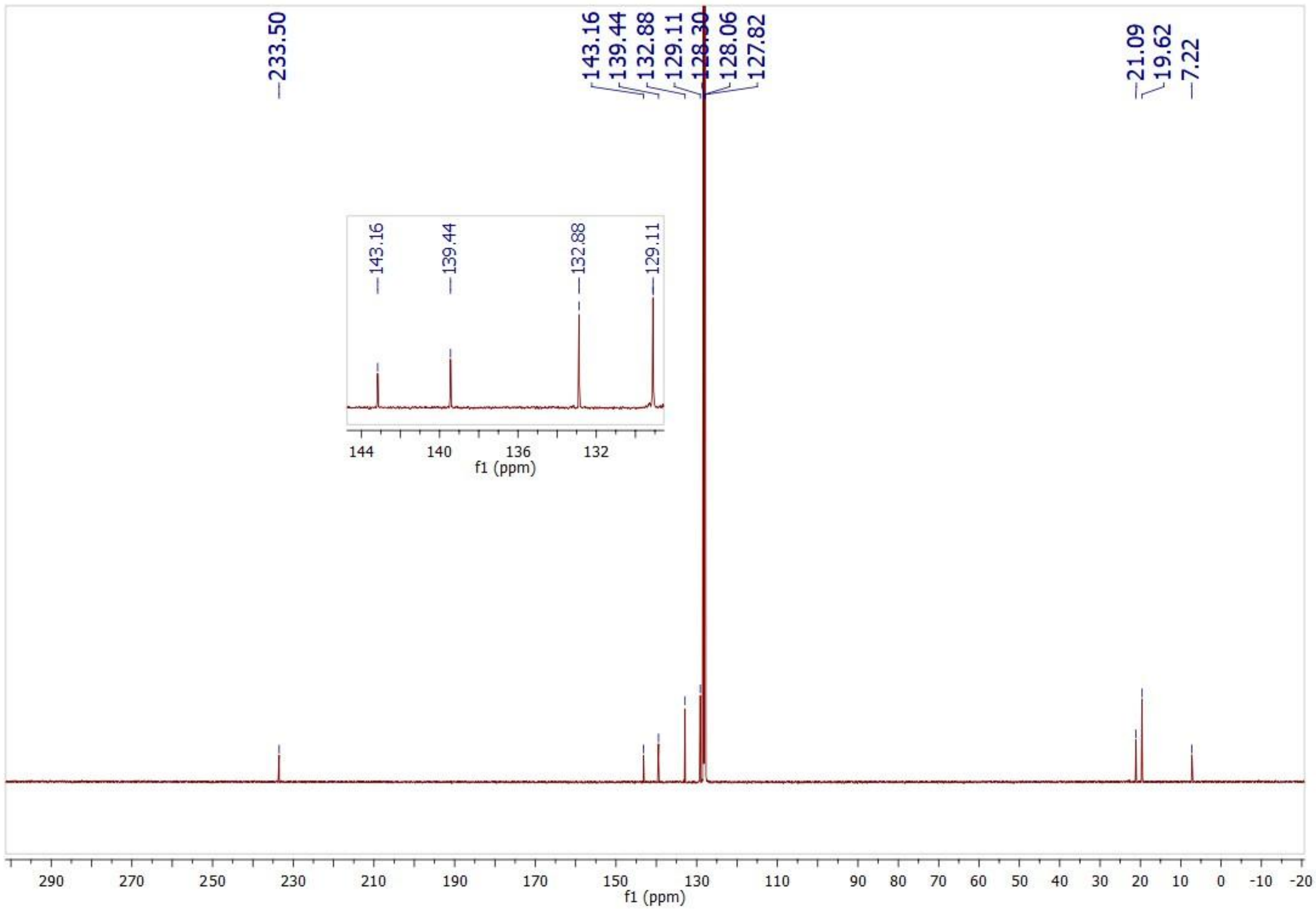

Figure S20. ^{13}C NMR spectrum of compound **8** in C_6D_6.

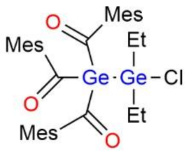

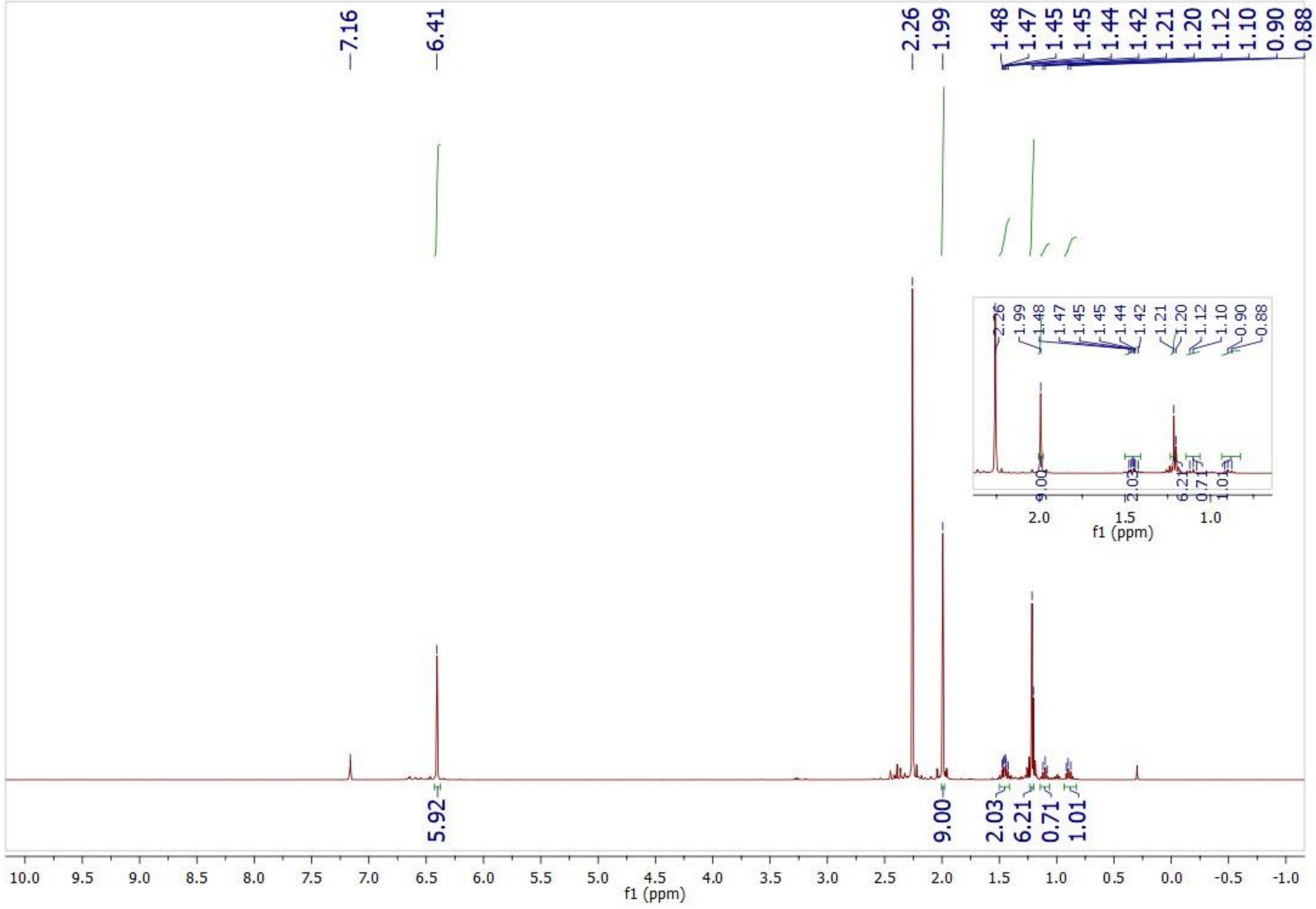

Figure S21. ^{1}H NMR spectrum of compound **9** in C_6D_6.

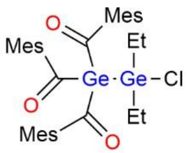

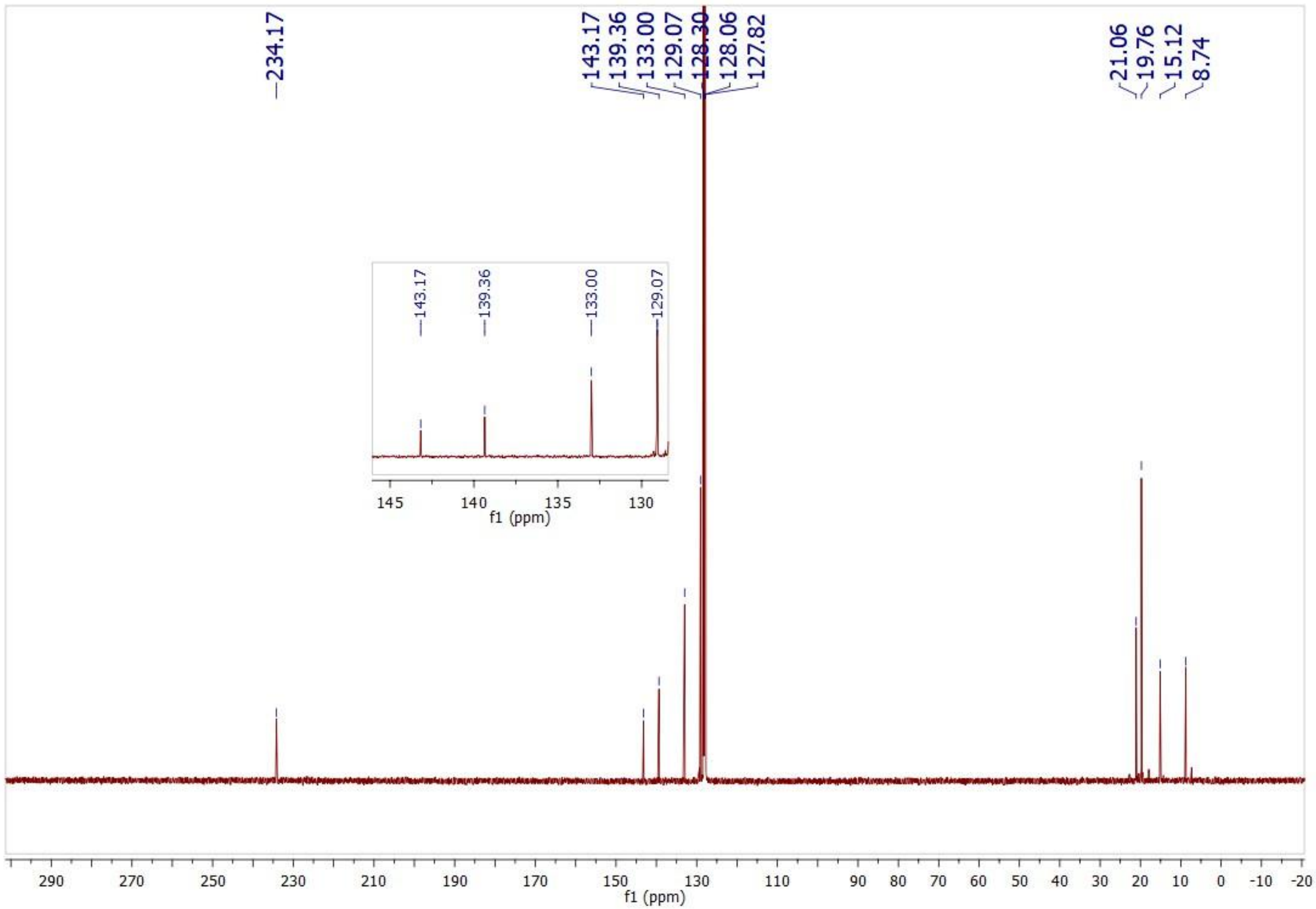

Figure S22. ^{13}C NMR spectrum of compound **9** in C_6D_6.

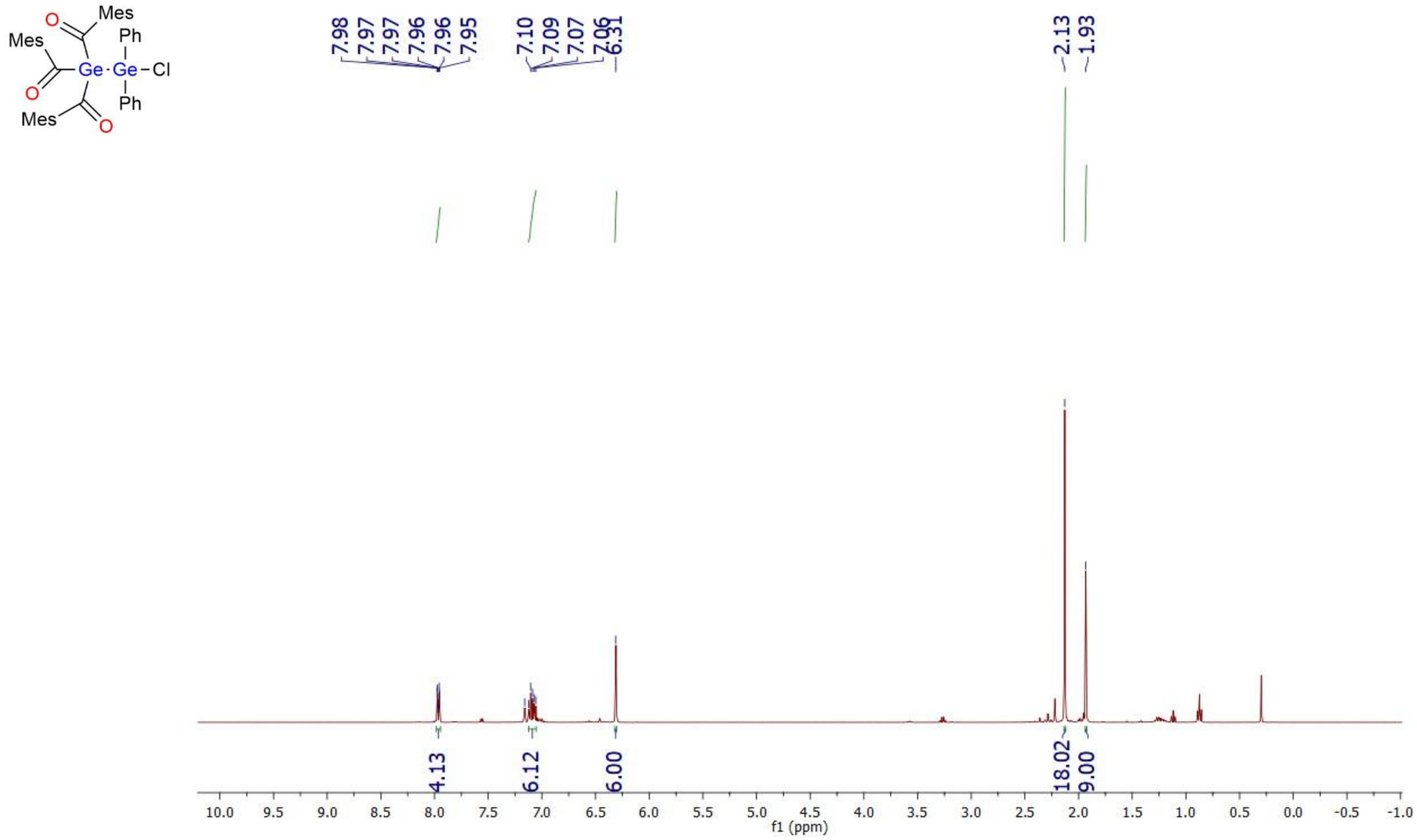

Figure S23. ^{1}H NMR spectrum of compound **10** in C_6D_6.

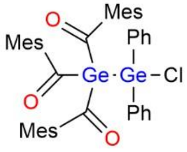

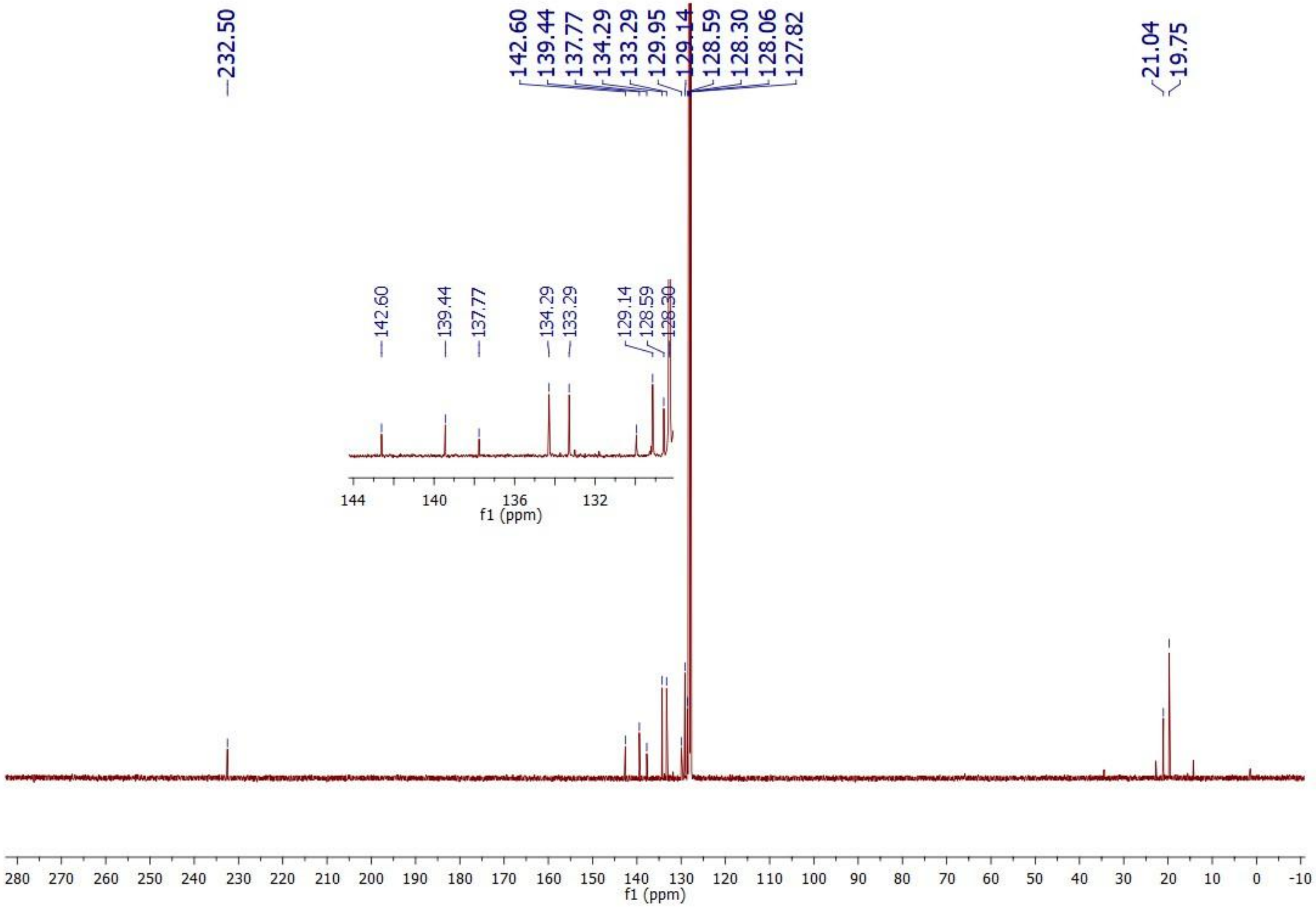

Figure S24. ^{13}C NMR spectrum of compound **10** in C_6D_6.

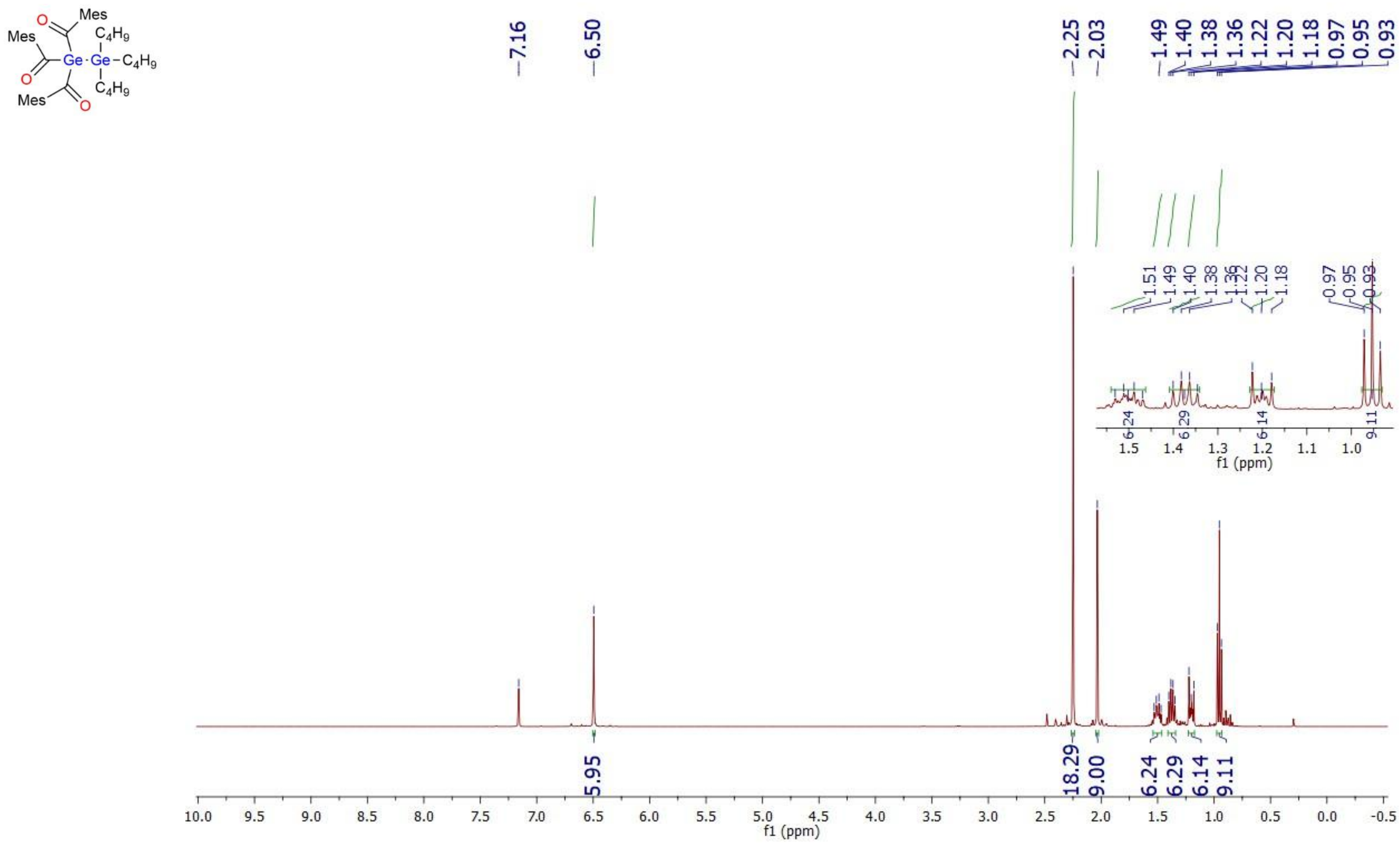

Figure S25. ^{1}H NMR spectrum of compound **11** in C_6D_6.

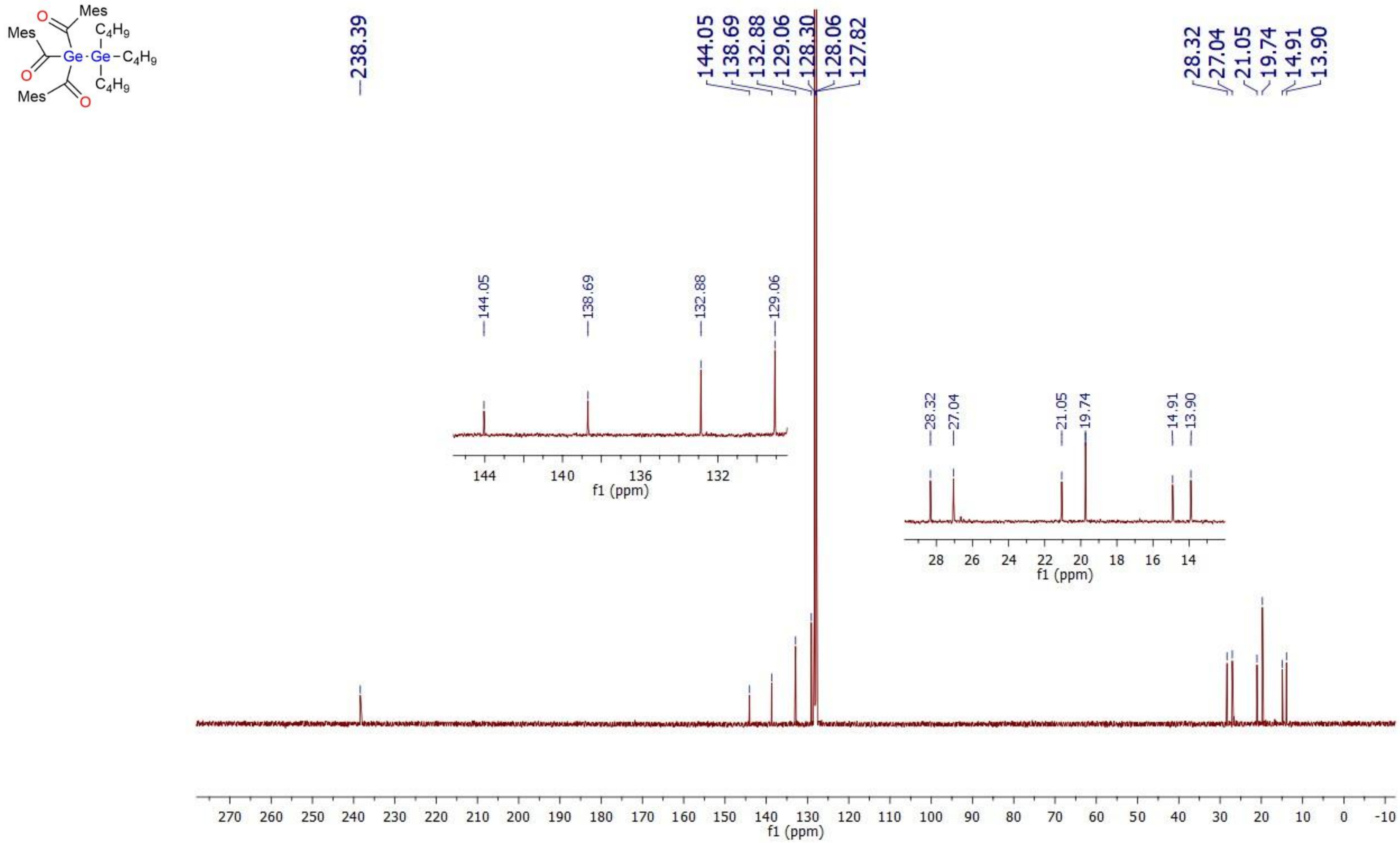

Figure S26. ^{13}C NMR spectrum of compound **11** in C_6D_6.

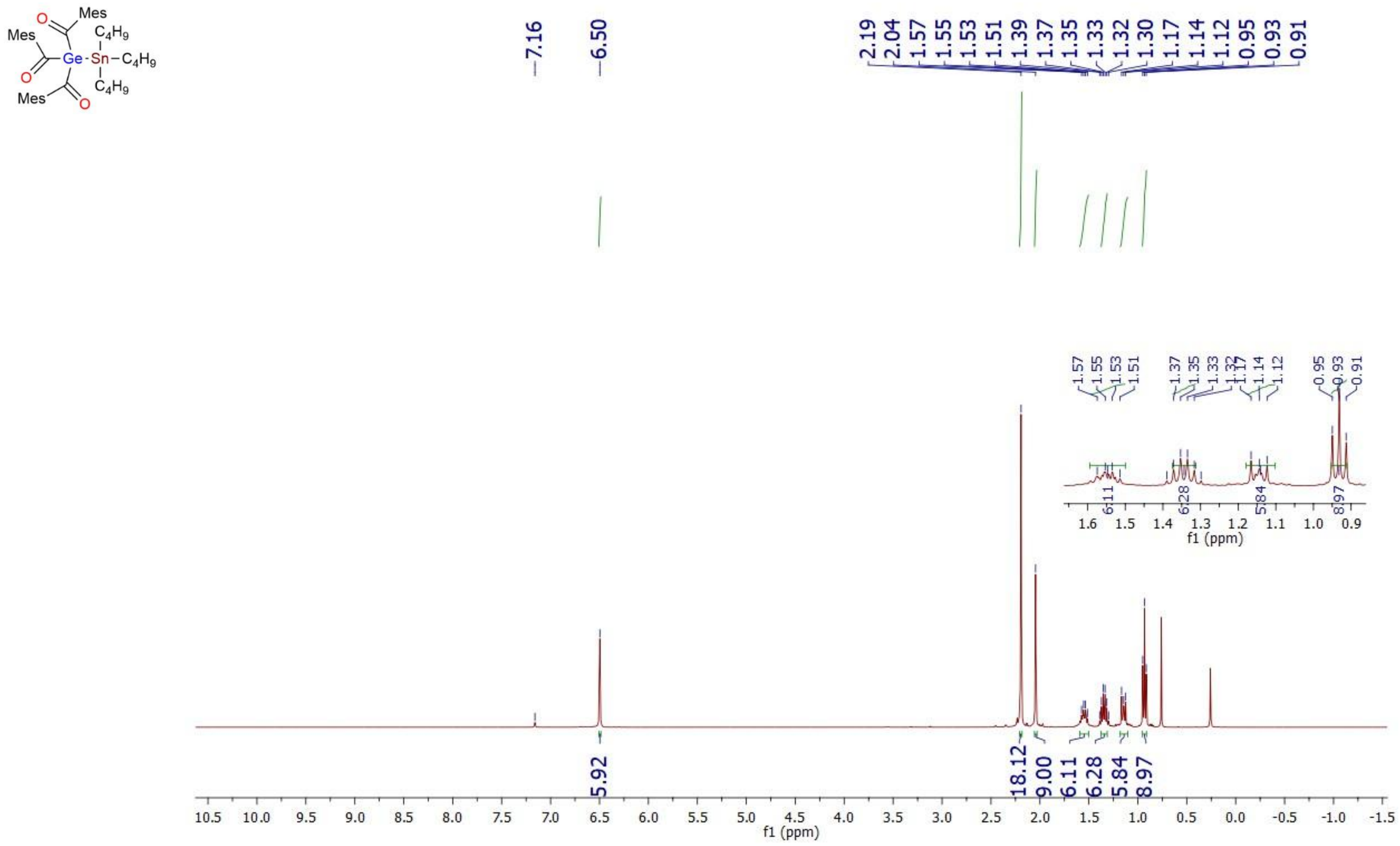

Figure S27. ^{1}H NMR spectrum of compound **12** in C_6D_6.

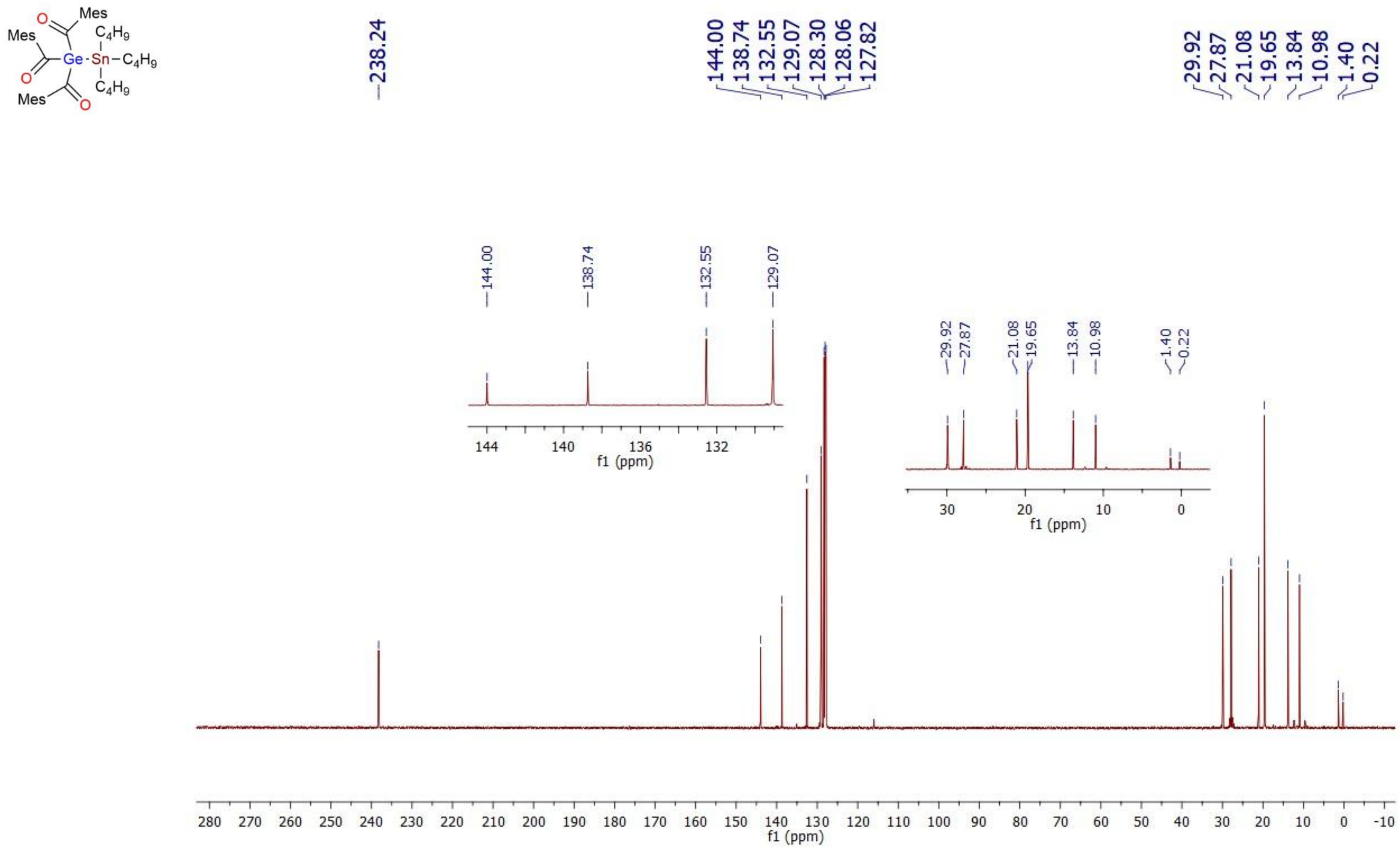

Figure S28. ^{13}C NMR spectrum of compound **12** in C_6D_6.

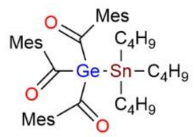

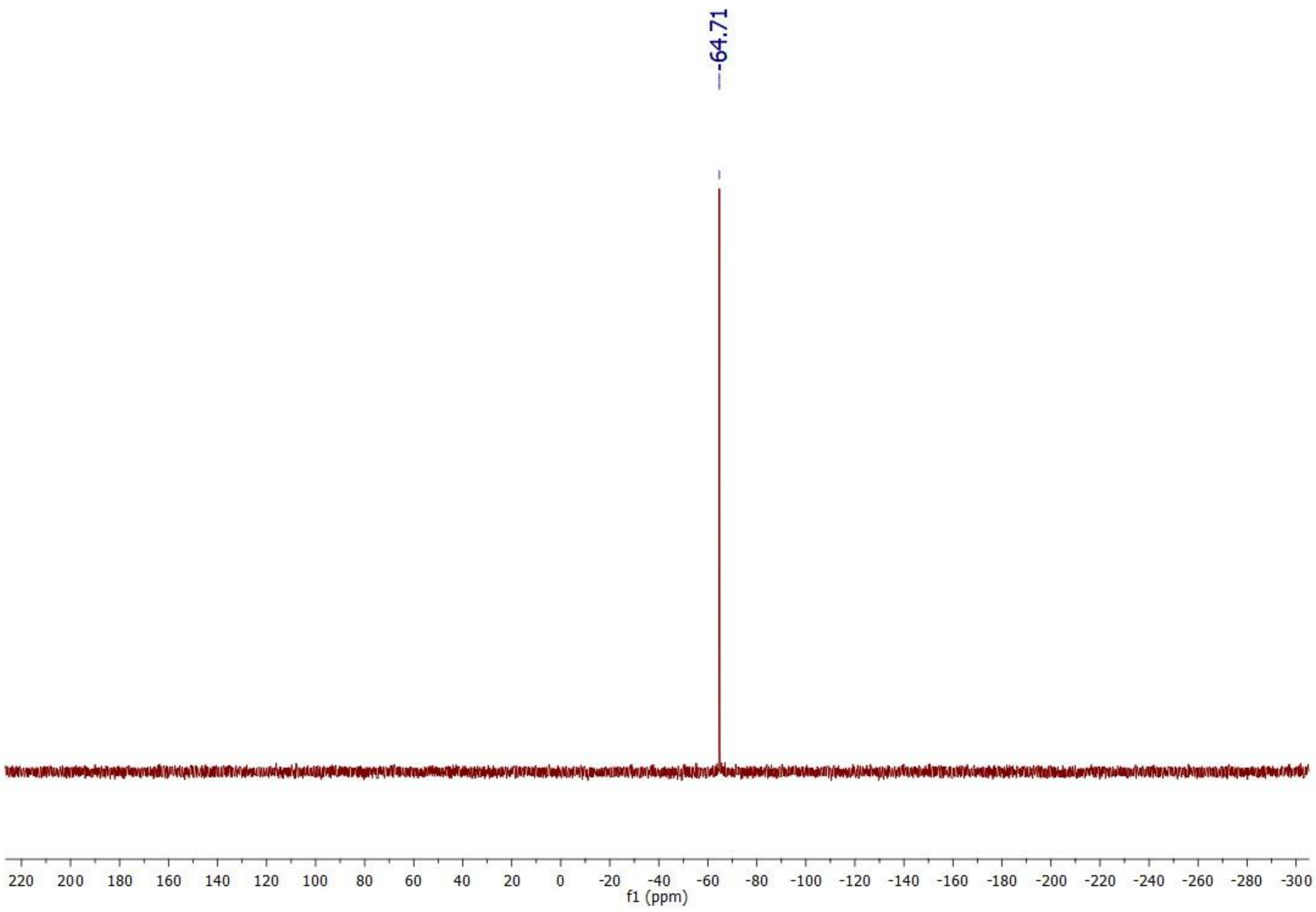

Figure S29. ^{119}Sn NMR spectrum of compound **12** in C_6D_6.

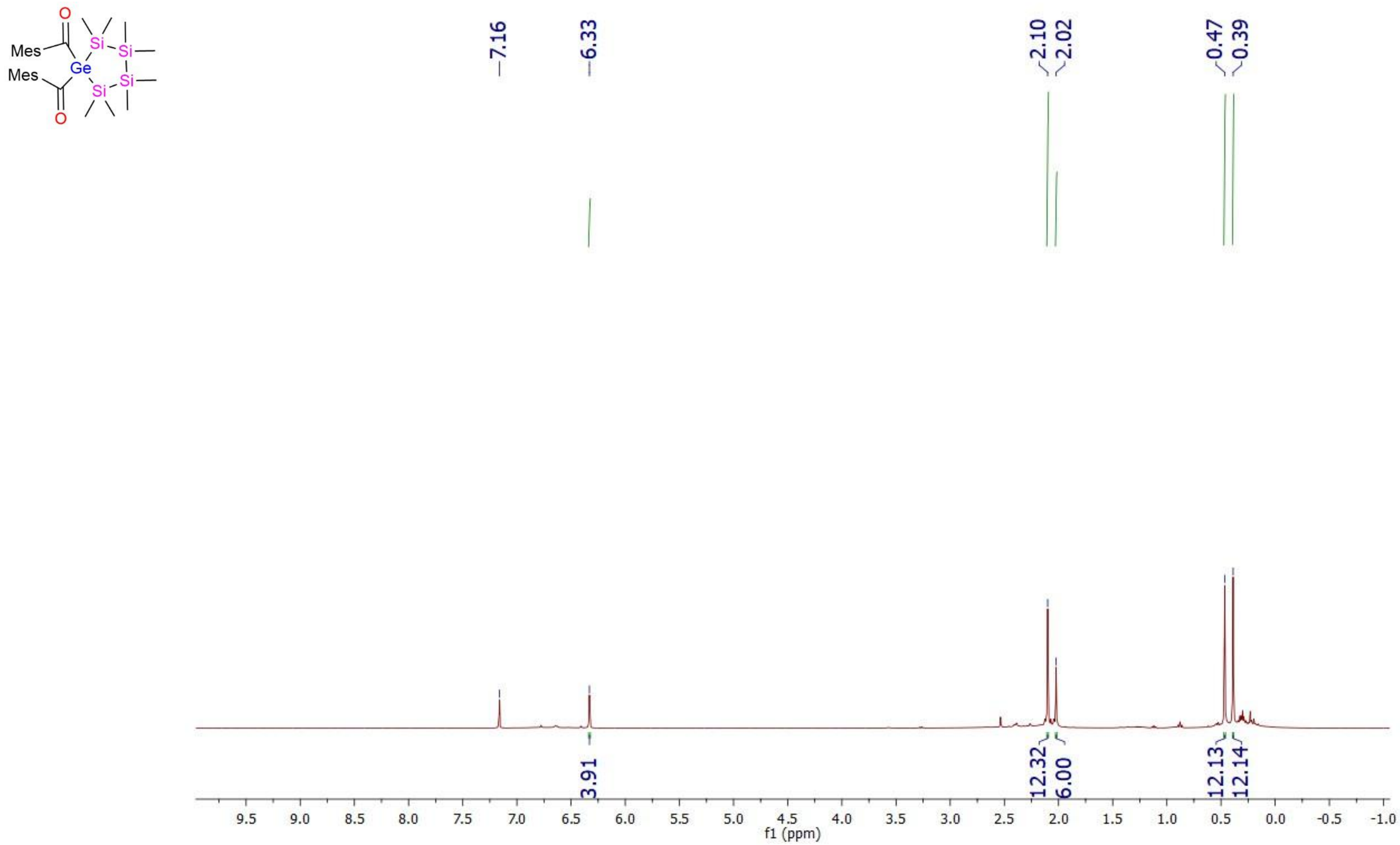

Figure S30. ^{1}H NMR spectrum of compound **14** in C_6D_6.

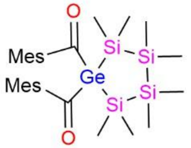

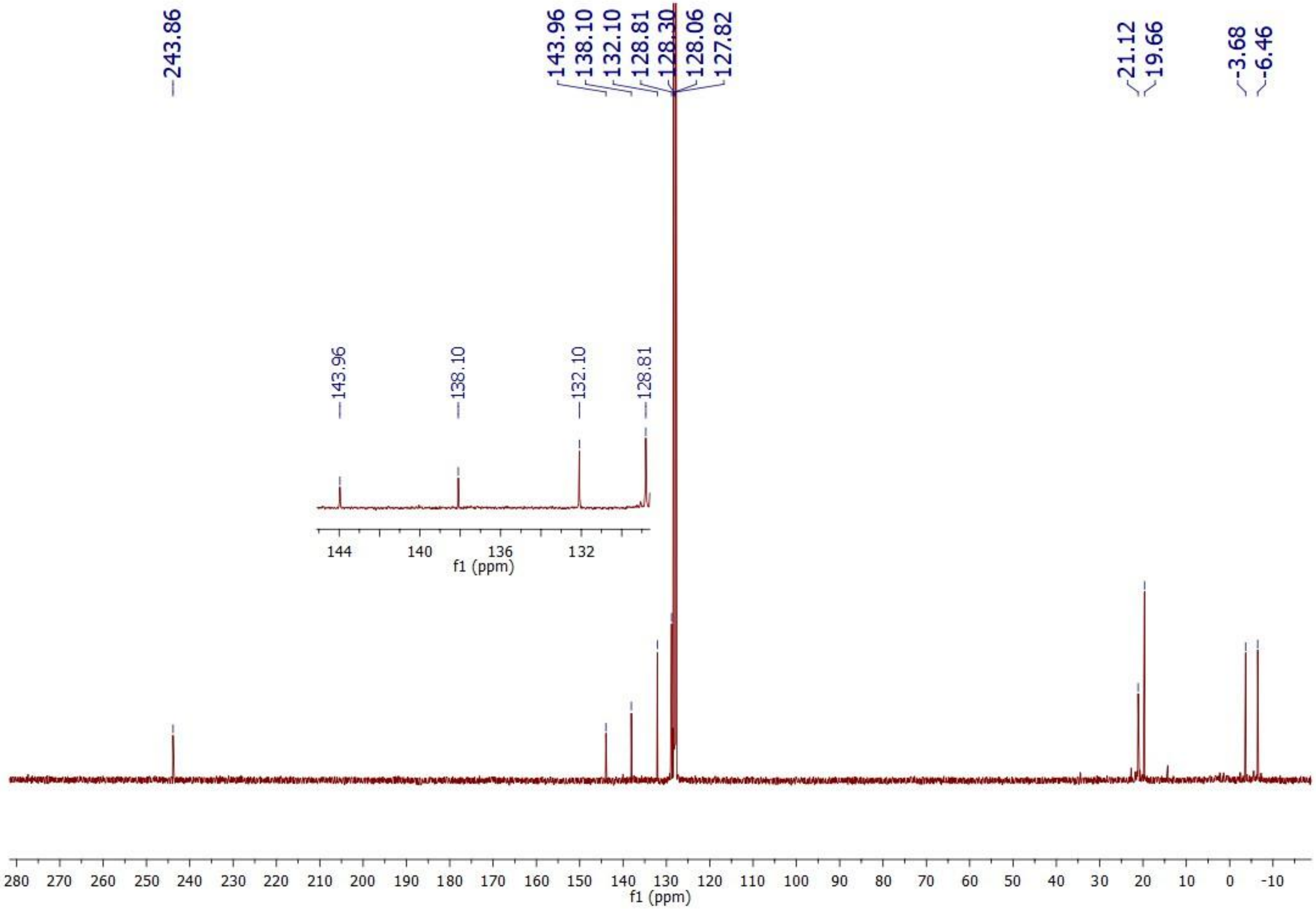

Figure S31. ^{13}C NMR spectrum of compound **14** in C_6D_6.

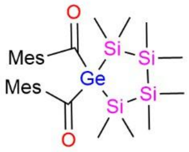

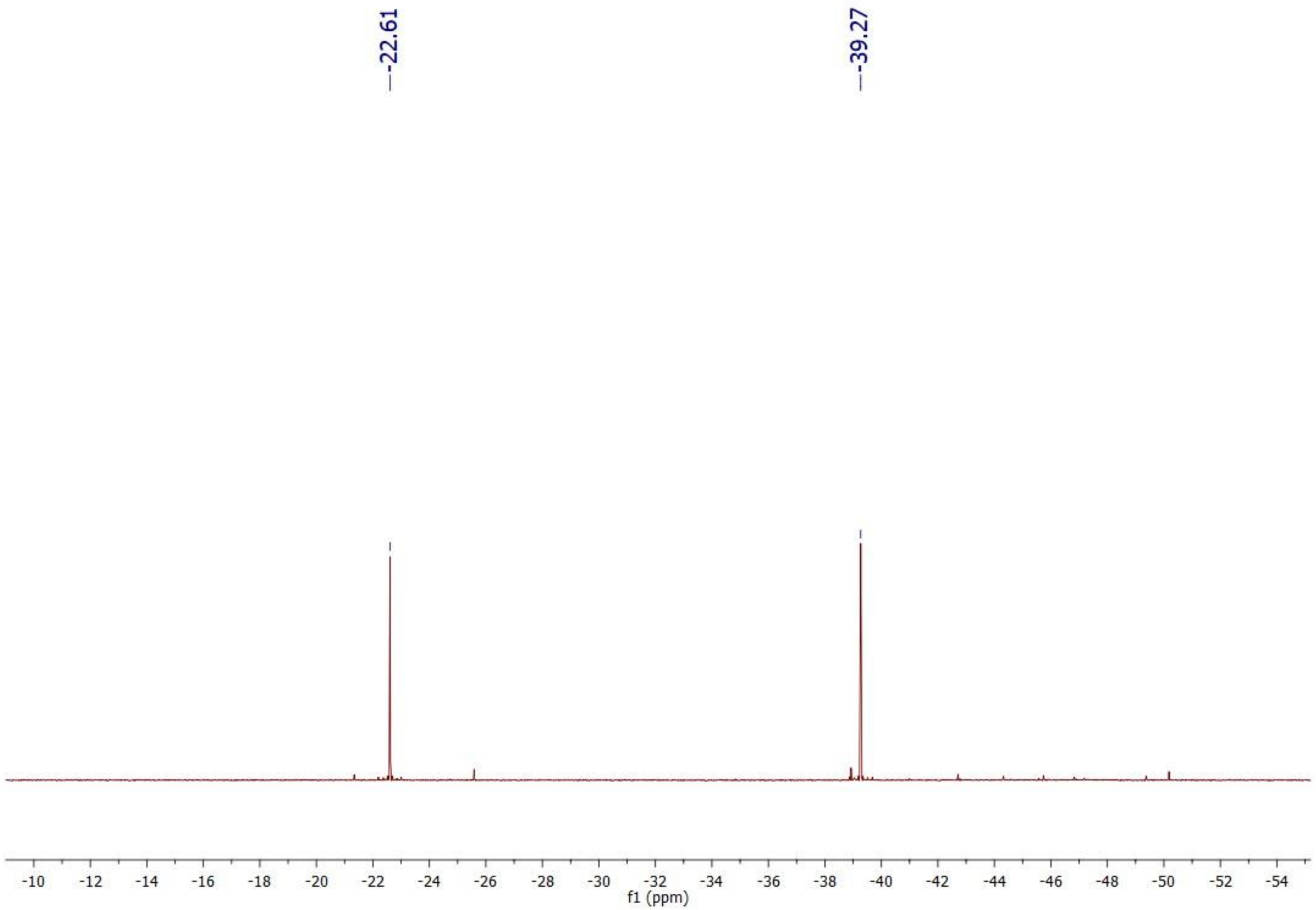

Figure S32. ^{29}Si NMR spectrum of compound **14** in C_6D_6.

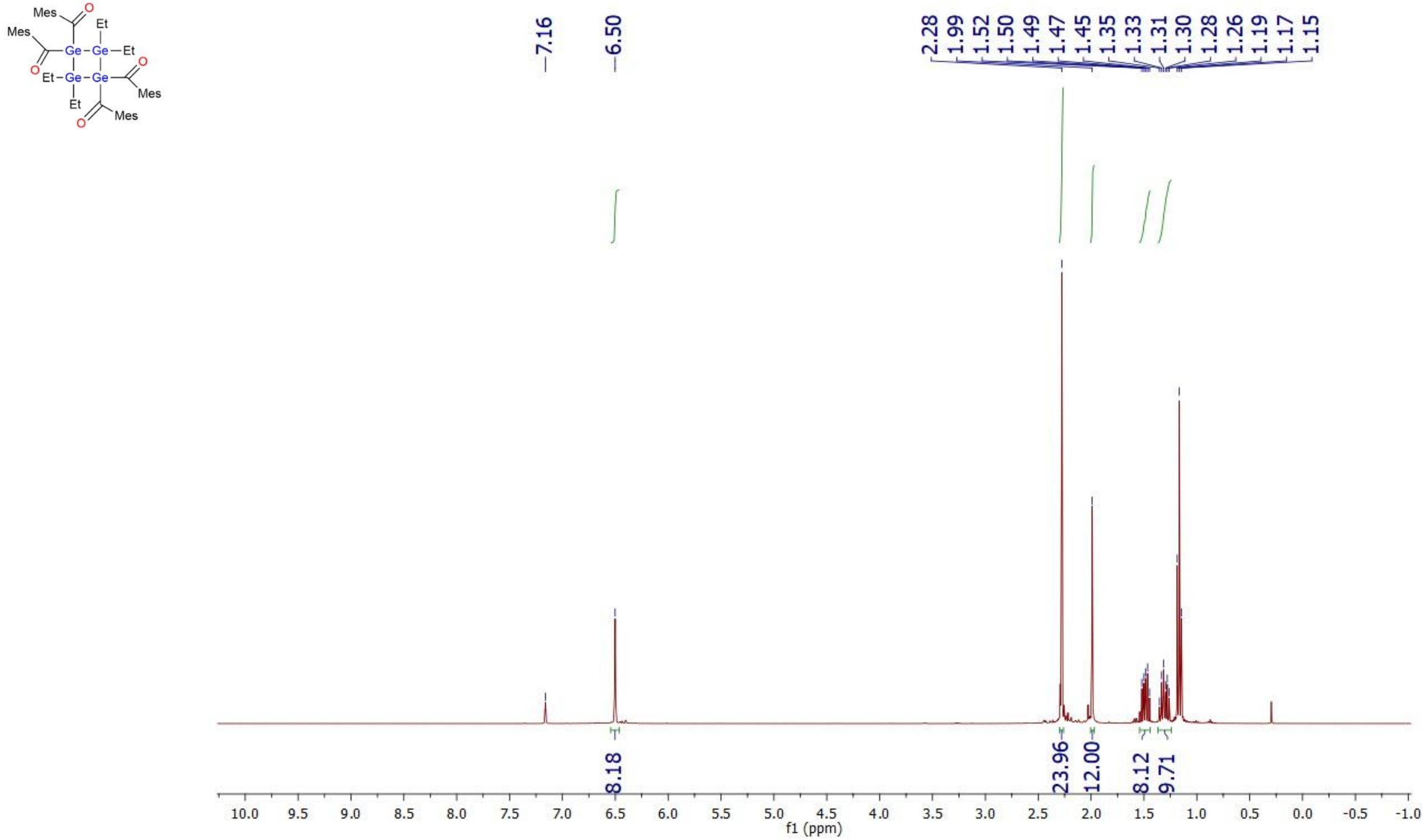

Figure S33. ^{1}H NMR spectrum of compound **15** in C_6D_6.

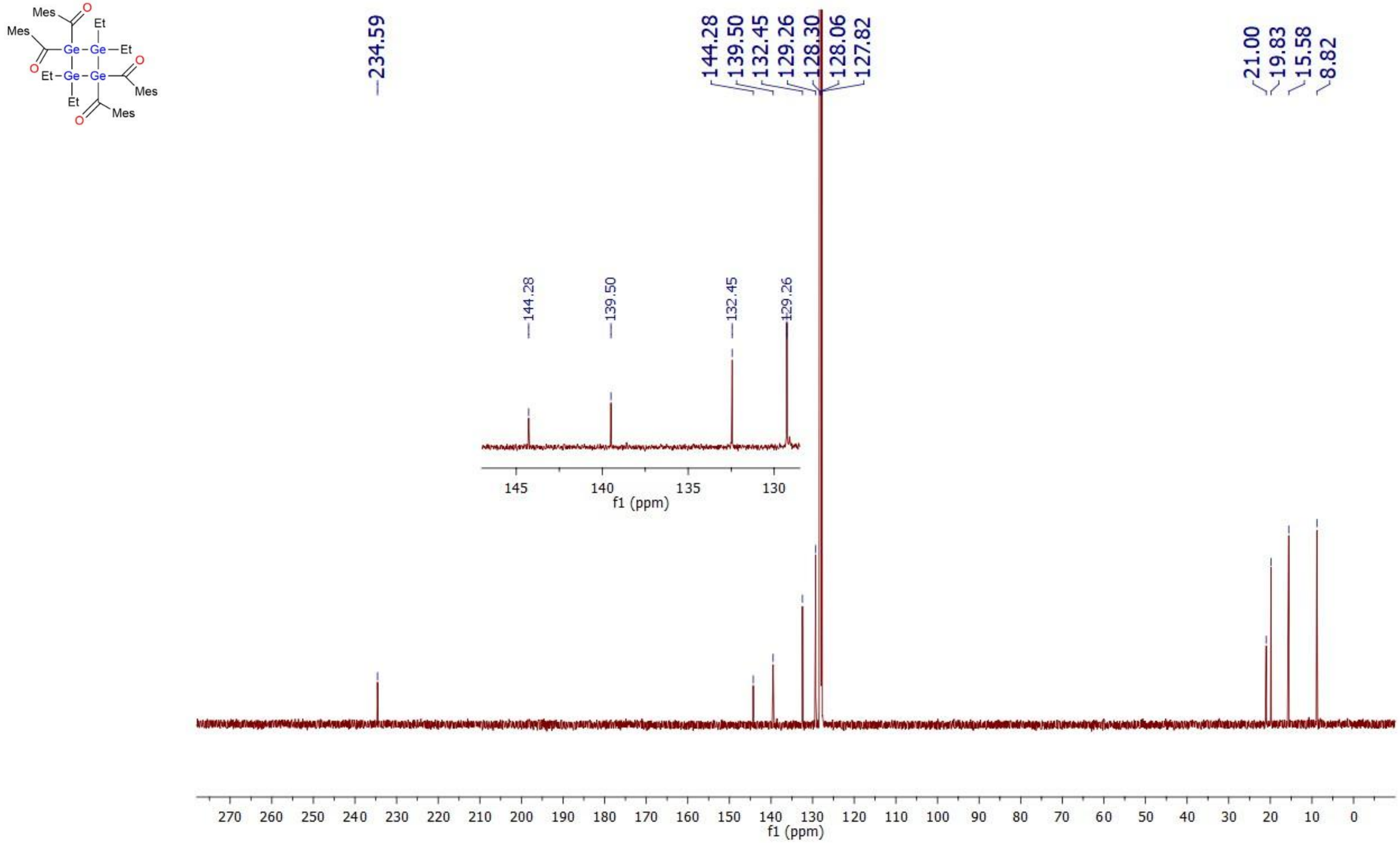

Figure S34. ^{13}C NMR spectrum of compound **15** in C_6D_6.

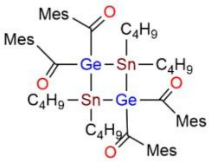

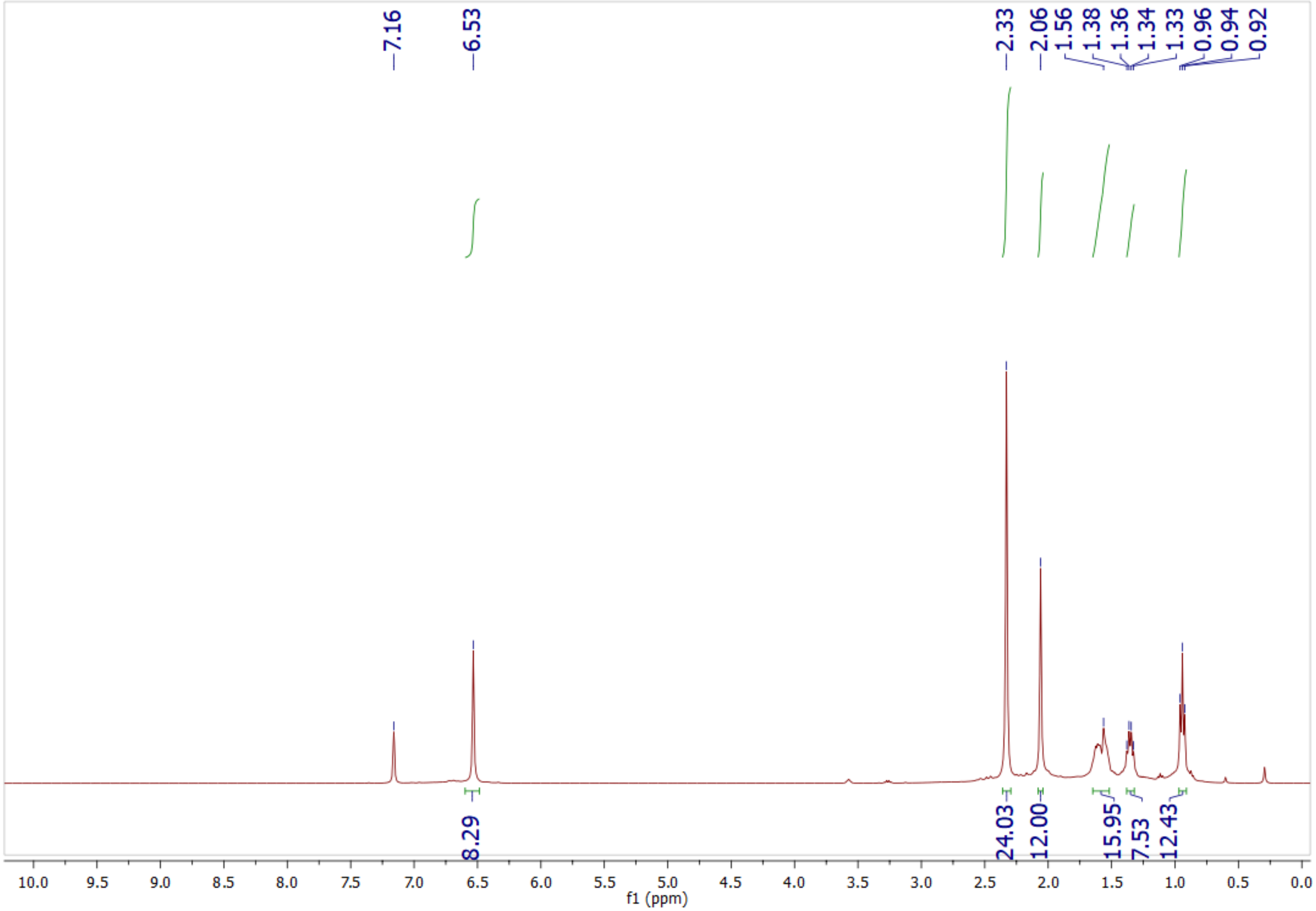

Figure S35. ^{1}H NMR spectrum of compound **16** in C_6D_6.

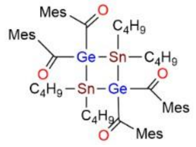

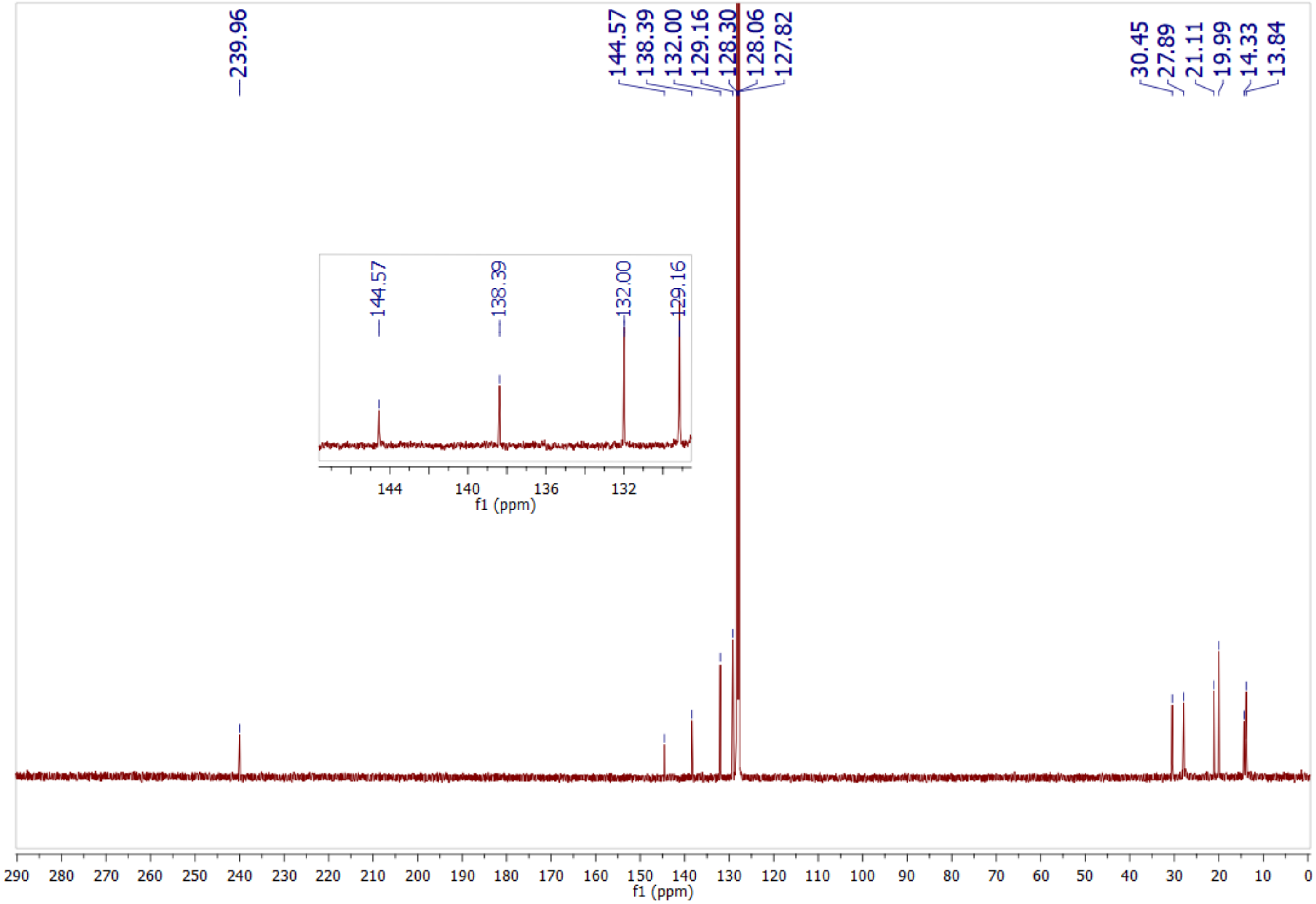

Figure S36. ^{13}C NMR spectrum of compound **16** in C_6D_6.

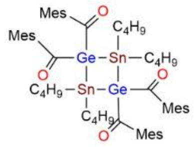

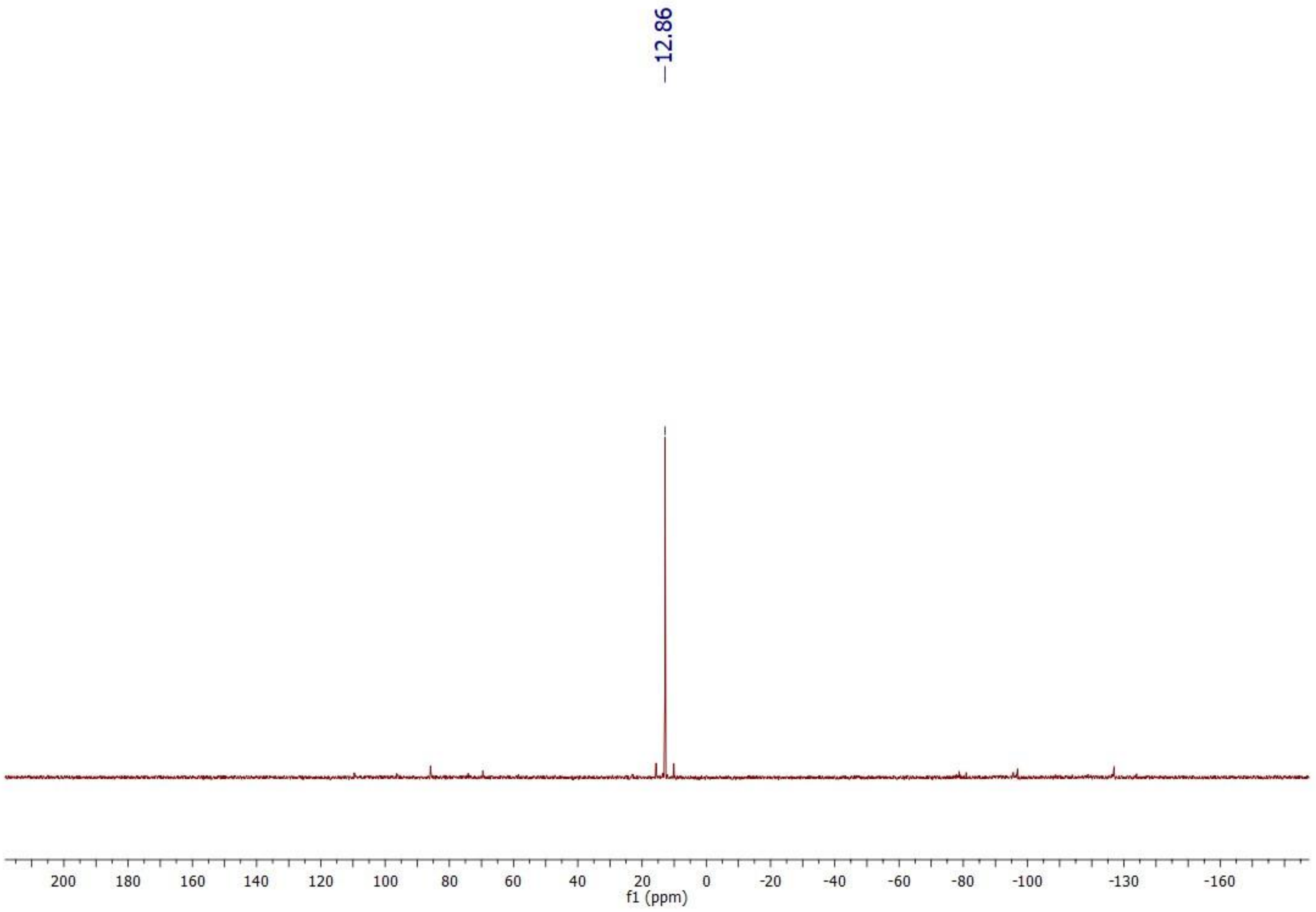

Figure S37. ^{119}Sn NMR spectrum of compound **16** in C_6D_6.

[S3]. ^{13}C and ^{29}Si NMR spectra of silyl substituted acylgermanes after aqueous workup.

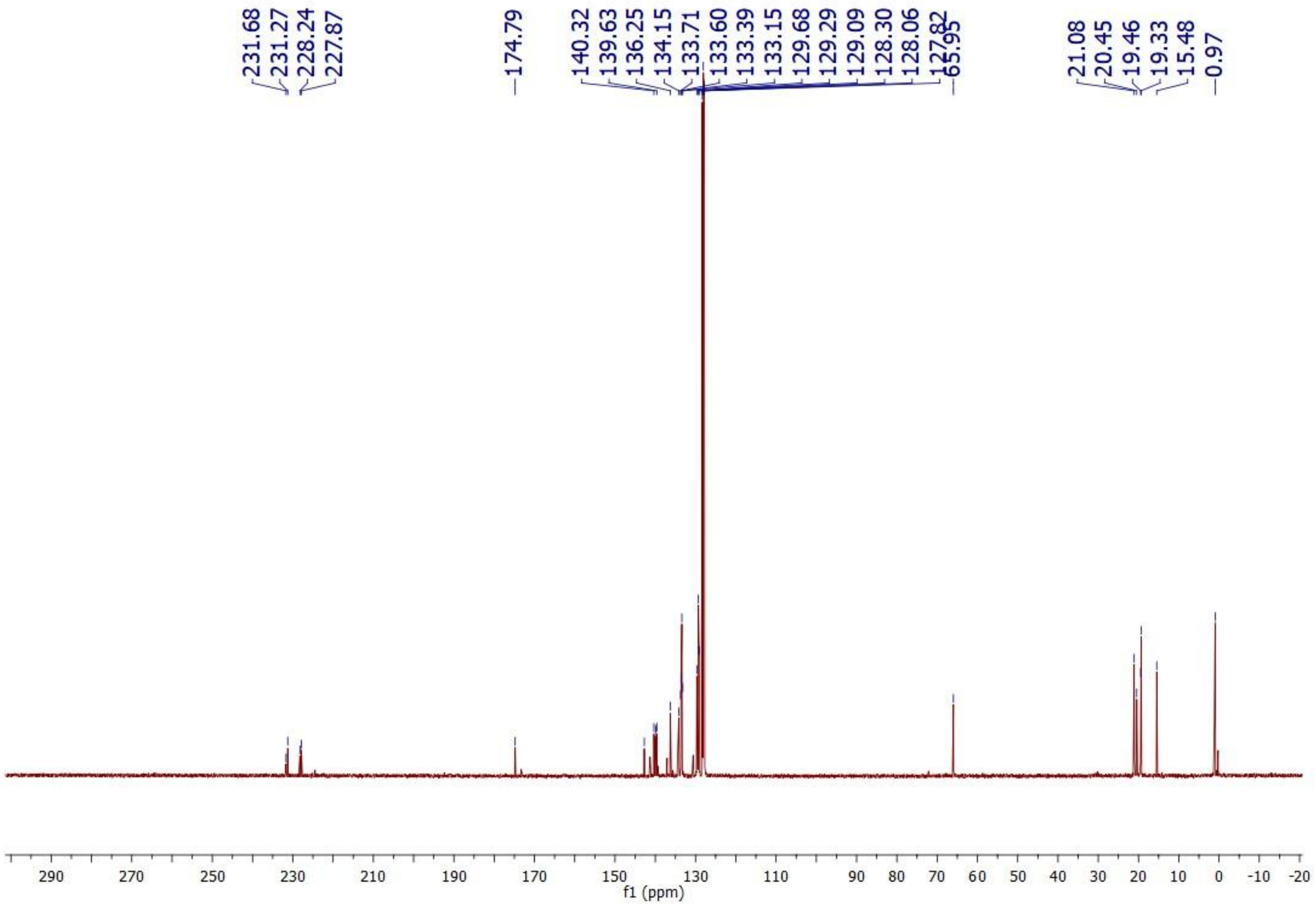

Figure S38. ^{13}C NMR spectrum of silyl substitute acylgermane and aqueous workup.

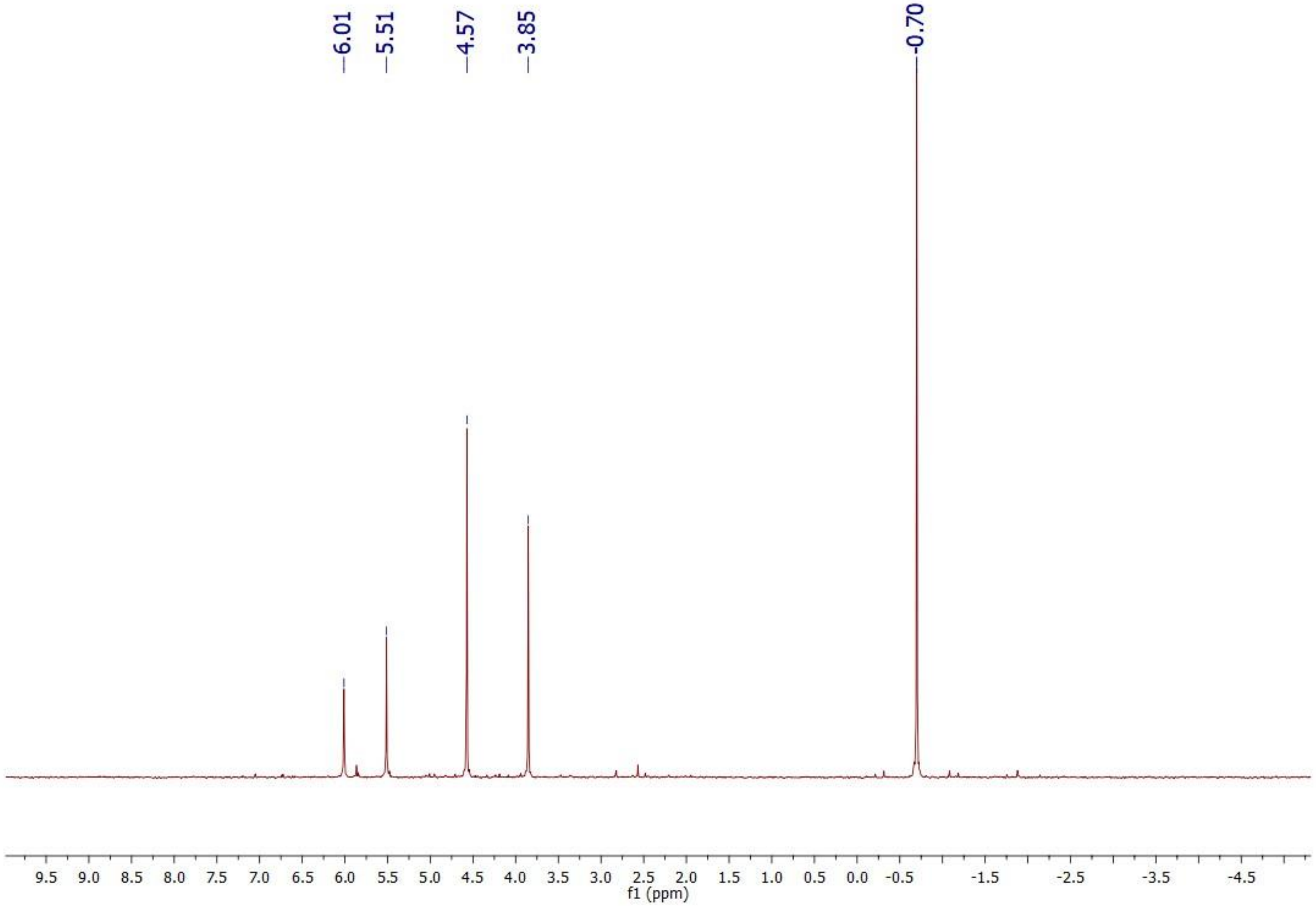

Figure S39. ^{29}Si NMR spectrum of silyl substitute acylgermane and aqueous workup.

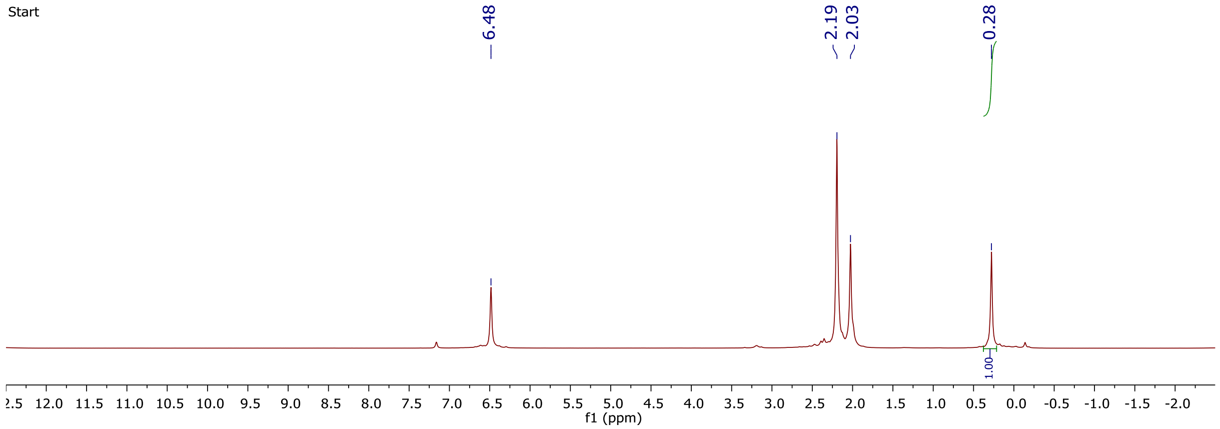

Start
6.48
2.19
2.03
0.28
1.00
f1 (ppm)

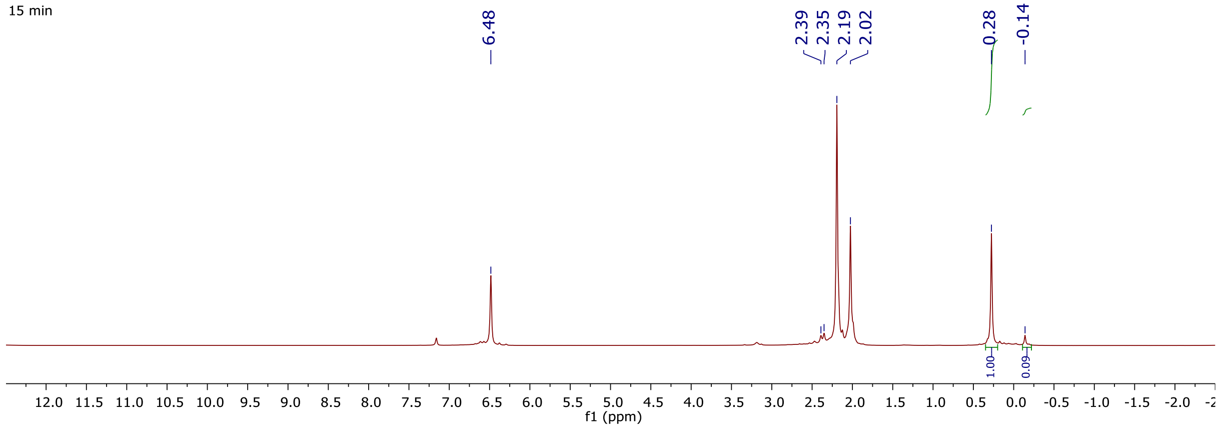

15 min
6.48
2.39
2.35
2.19
2.02
0.28
-0.14
1.00
0.09
f1 (ppm)

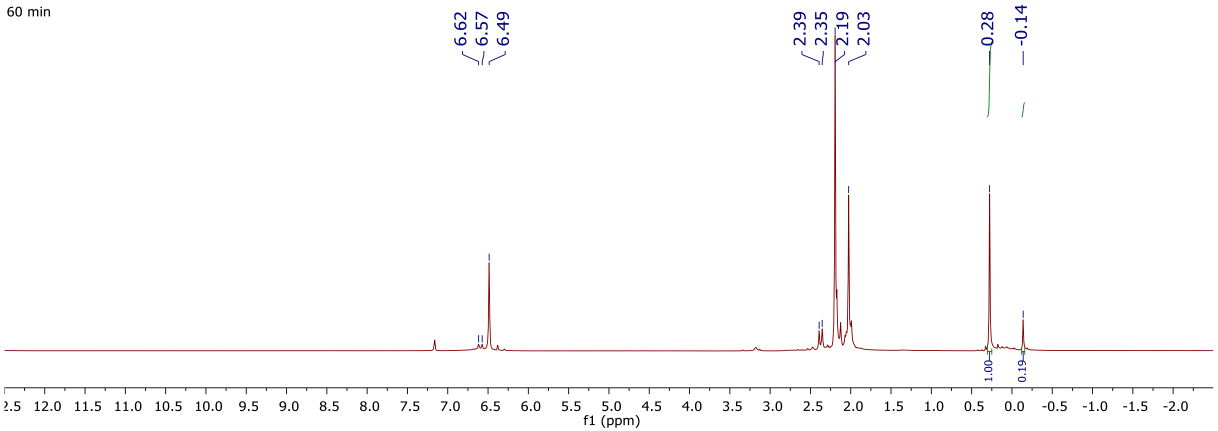
60 min
6.62
6.57
6.49
2.39
2.35
2.19
2.03
0.28
-0.14
1.00
0.19
f1 (ppm)

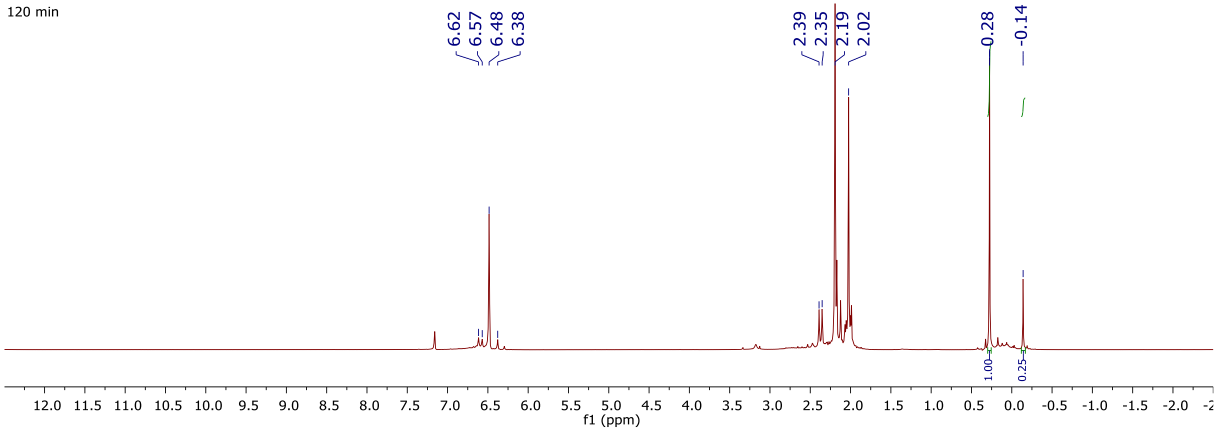
120 min
6.62
6.57
6.48
6.38
2.39
2.35
2.19
2.02
0.28
-0.14
1.00
0.25
f1 (ppm)

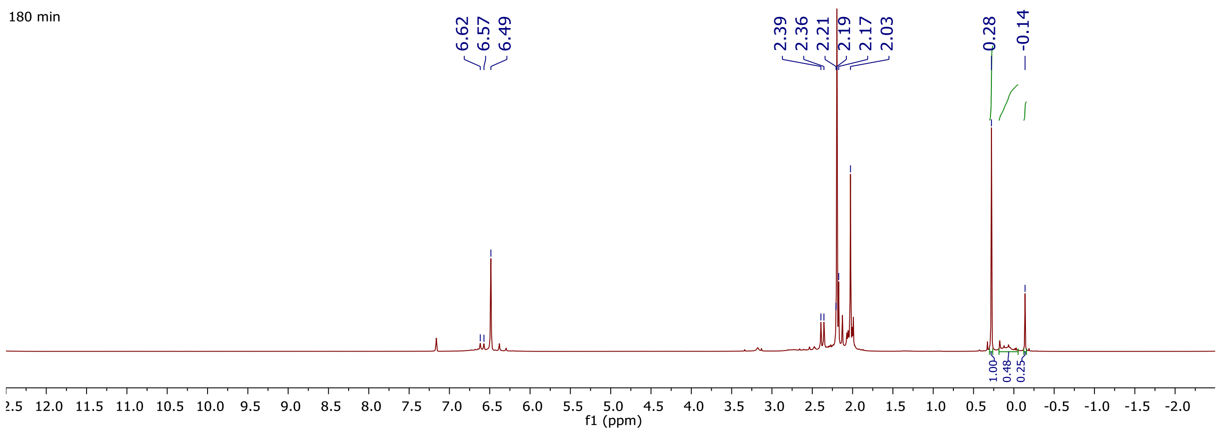
180 min
6.62
6.57
6.49
2.39
2.36
2.21
2.19
2.17
2.03
0.28
-0.14
1.00
0.48
0.25
f1 (ppm)

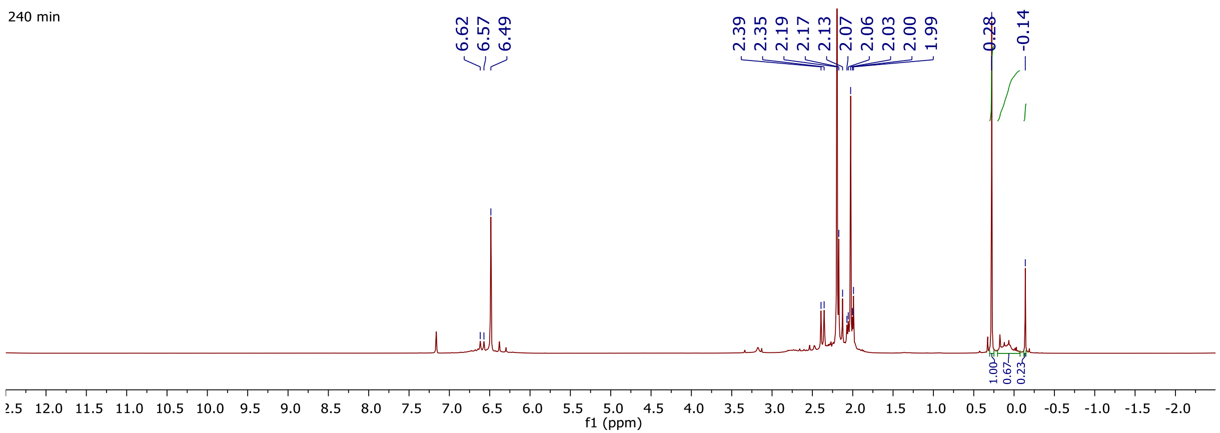
240 min
6.62
6.57
6.49
2.39
2.35
2.19
2.17
2.13
2.07
2.06
2.03
2.00
1.99
0.28
-0.14
1.00
0.67
0.23
f1 (ppm)

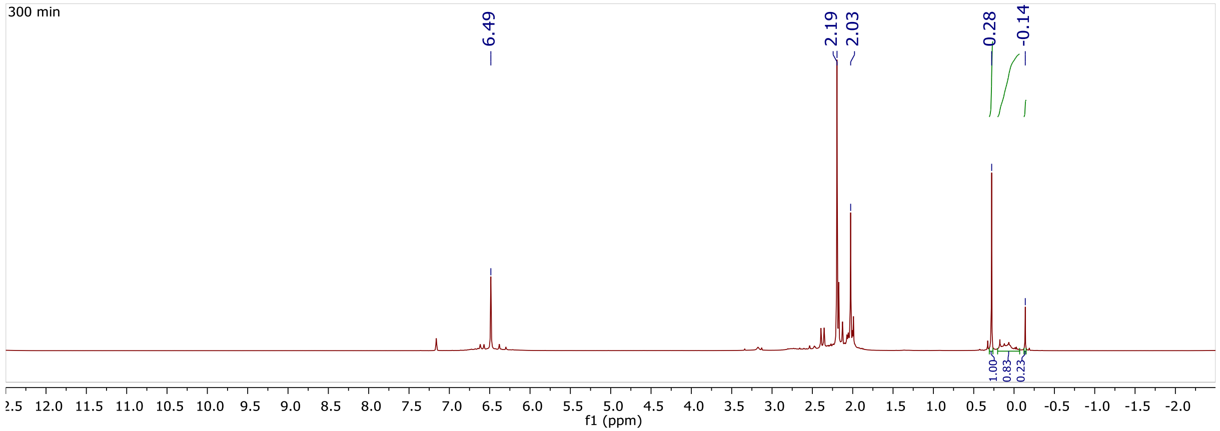
300 min
6.49
2.19
2.03
0.28
-0.14
1.00
0.83
0.23
f1 (ppm)

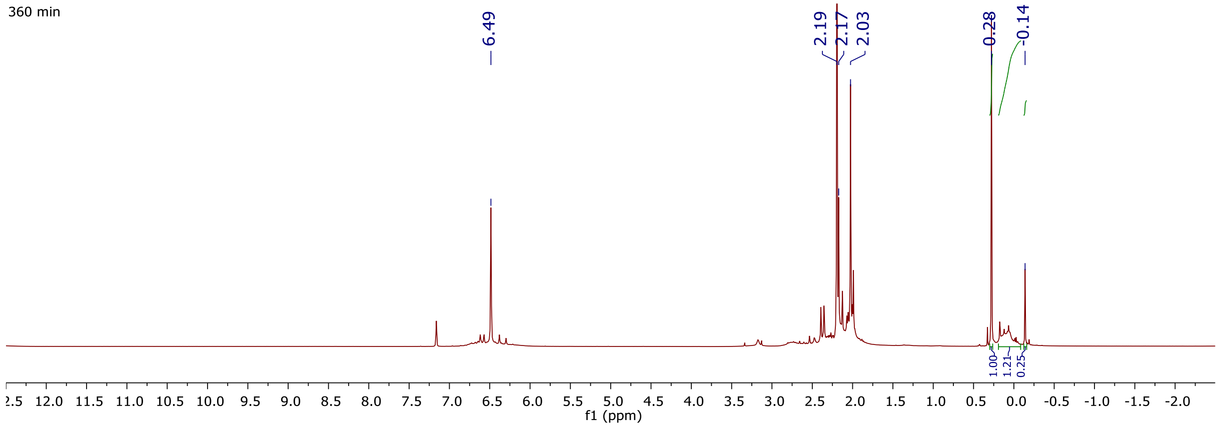
360 min
6.49
2.19
2.17
2.03
0.28
-0.14
1.00
1.21
0.25
f1 (ppm)

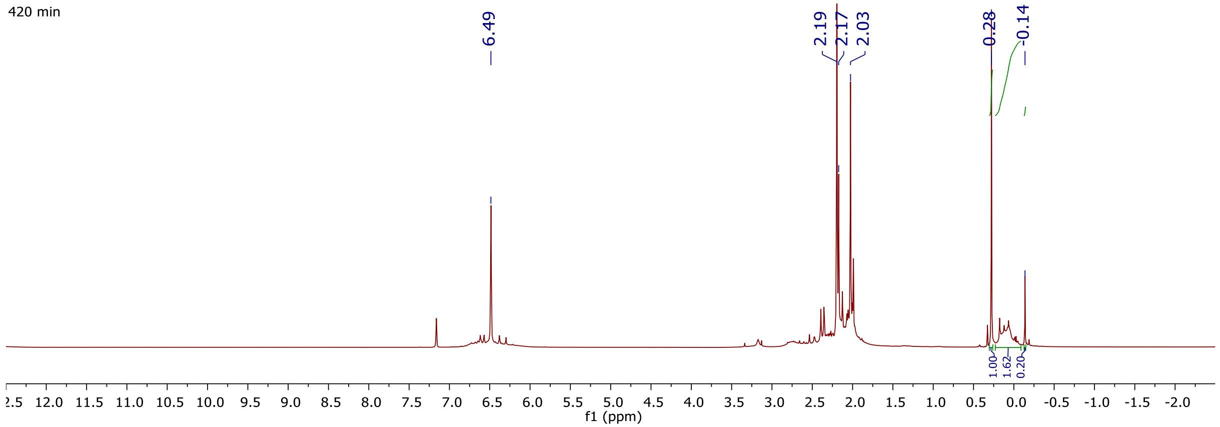
420 min
6.49
2.19
2.17
2.03
0.28
-0.14
1.00
1.62
0.20
f1 (ppm)

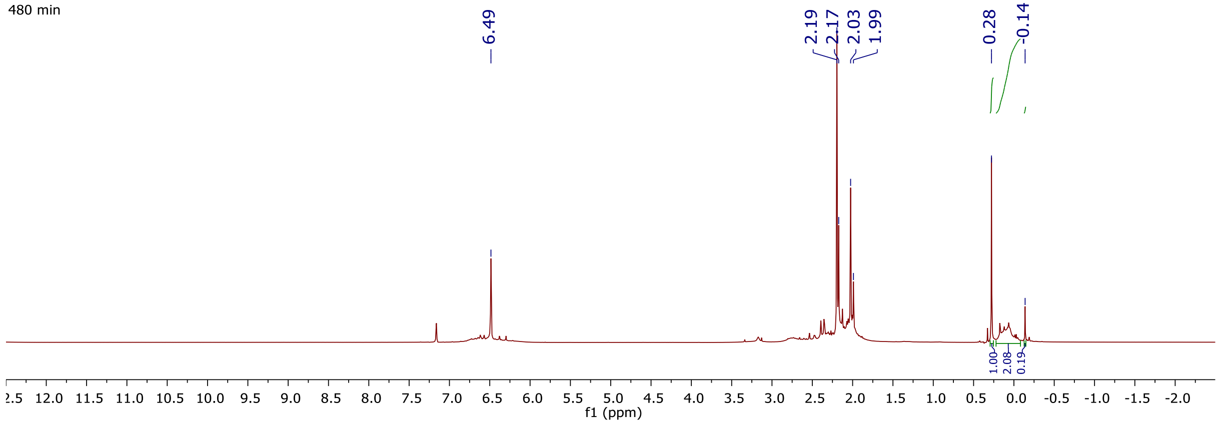
480 min
6.49
2.19
2.17
2.03
1.99
0.28
-0.14
1.00
2.08
0.19
f1 (ppm)

540 min

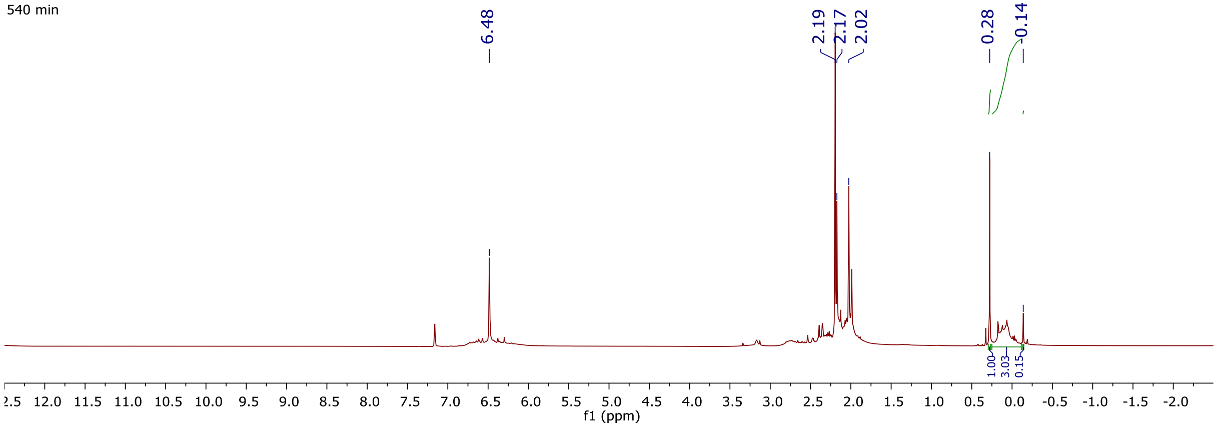

600 min

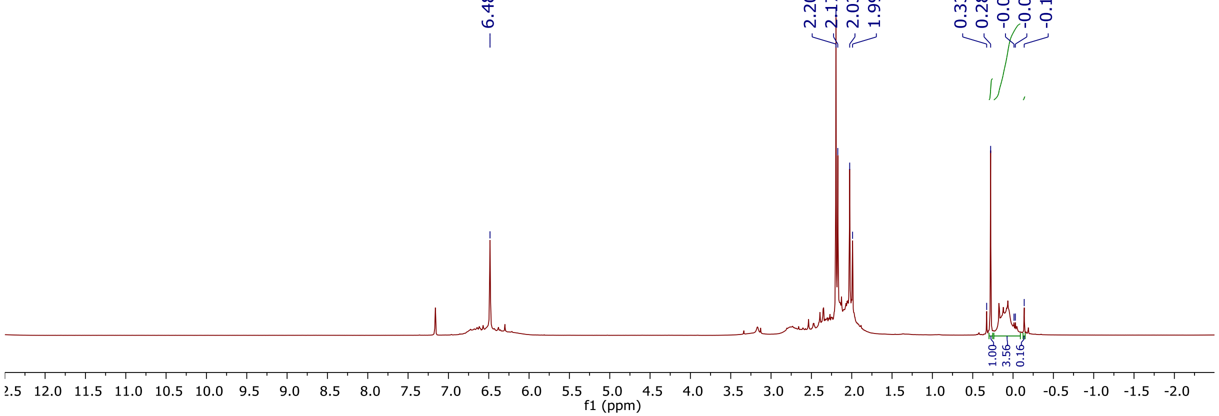

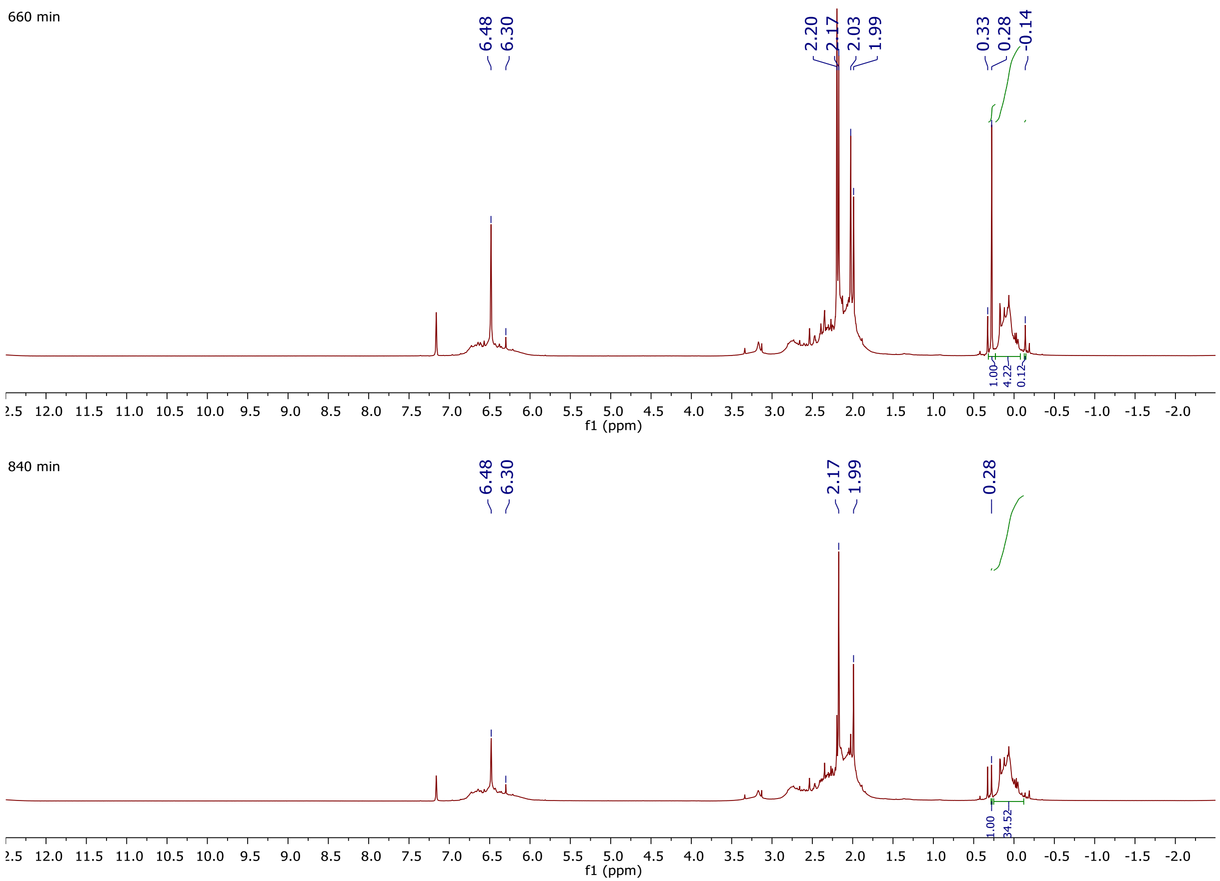

Figure S40. Series of ^{1}H spectra to monitor the keto-enol equilibrium and subsequent degradation process.

[S4]. UV-Vis absorption spectra

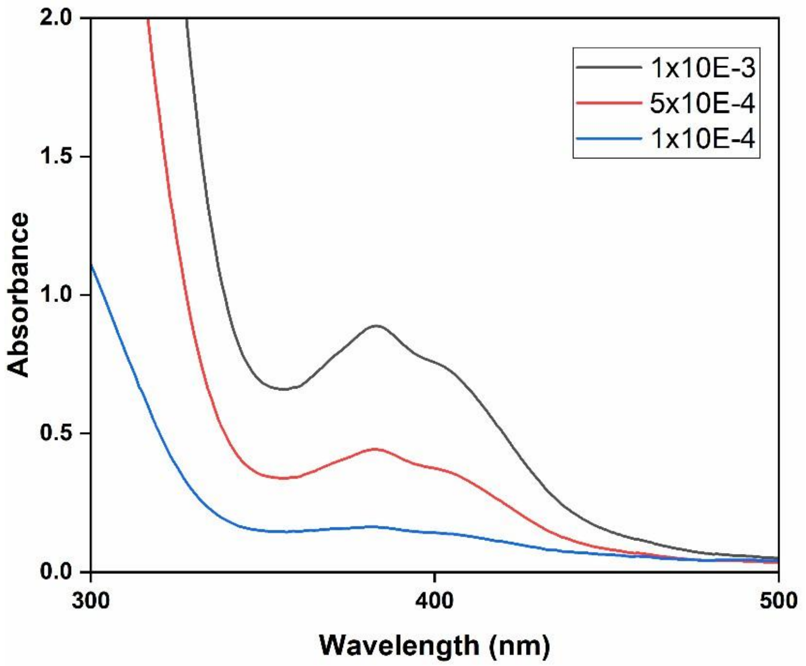

Figure S41. UV-Vis absorption spectrum of compound **2**

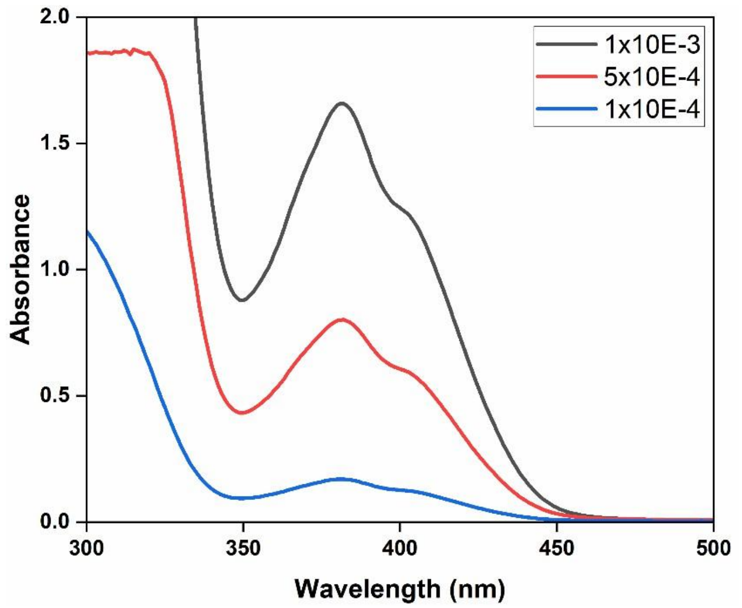

Figure S42. UV-Vis absorption spectrum of compound **3**.

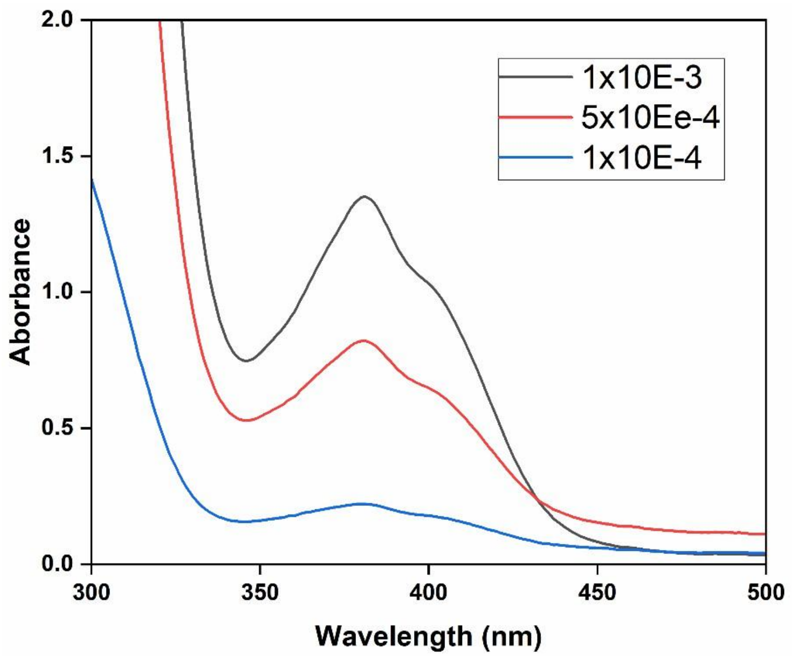

Figure S43. UV-Vis absorption spectrum of compound **5**

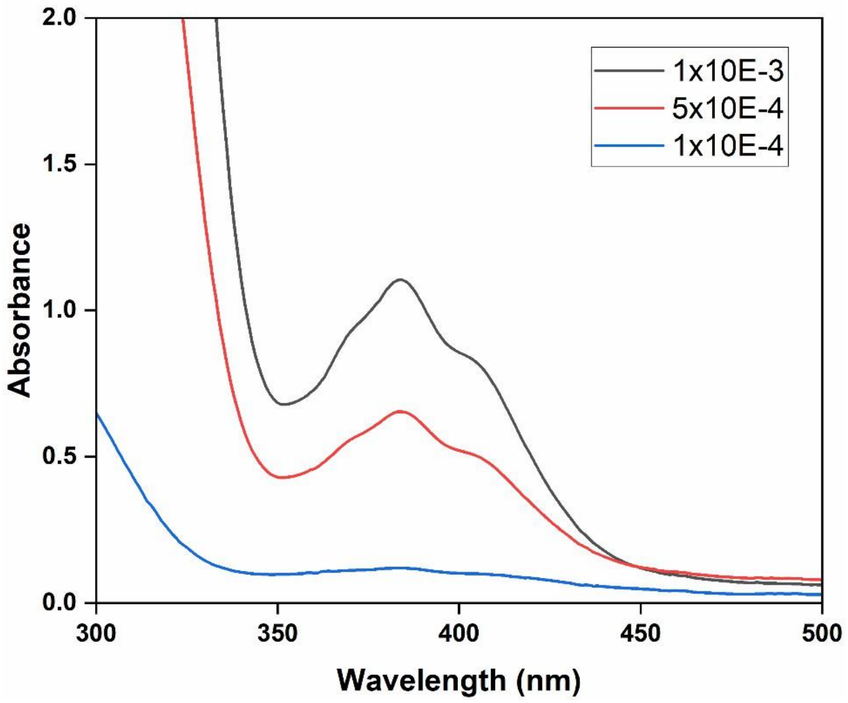

Figure S44. UV-Vis absorption spectrum of compound **7**

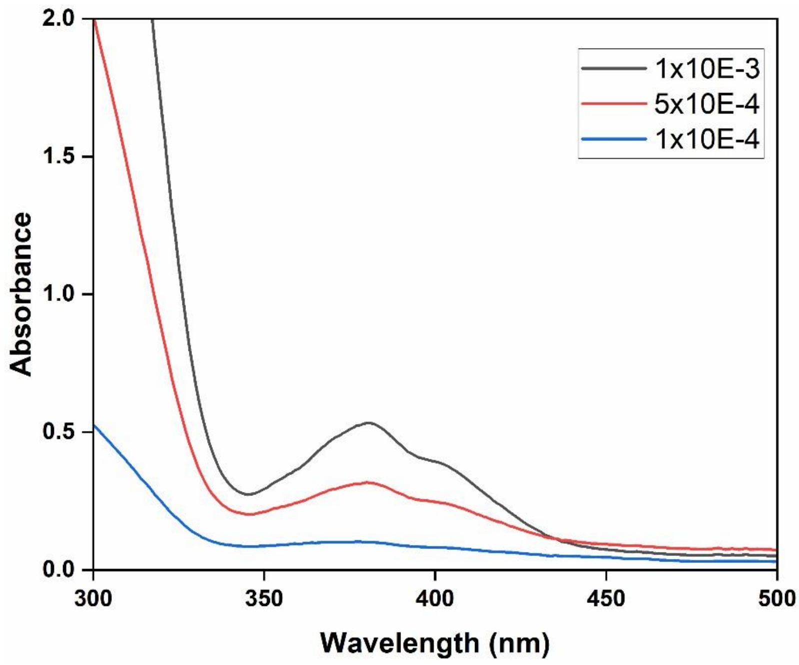

Figure S45. UV-Vis absorption spectrum of compound **9**.

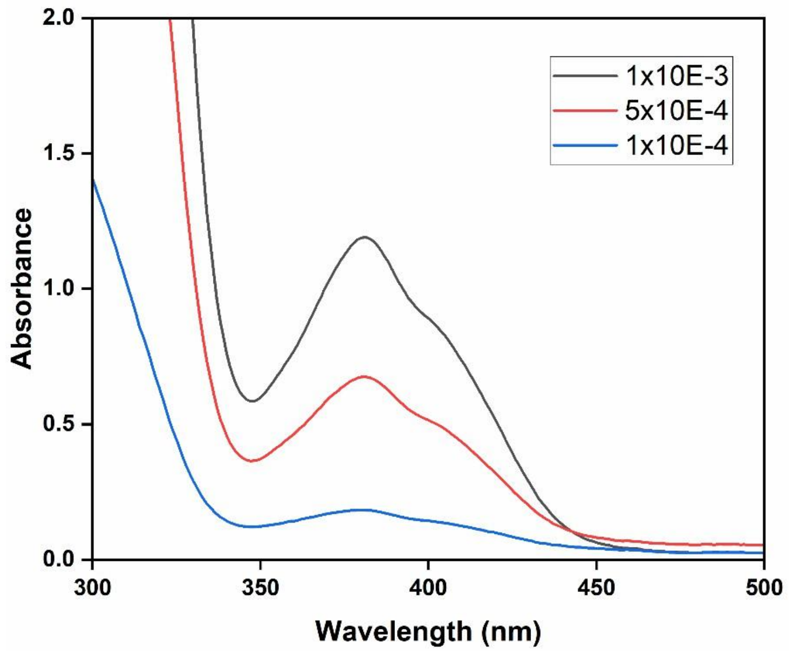

Figure S46. UV-Vis absorption spectrum of compound **10**

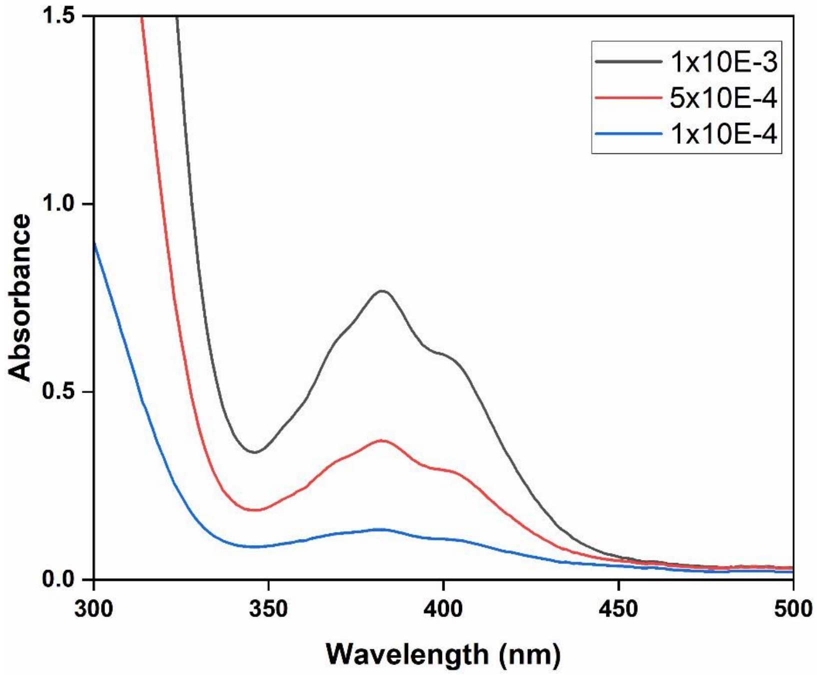

Figure S47. UV-Vis absorption spectrum of compound **11**

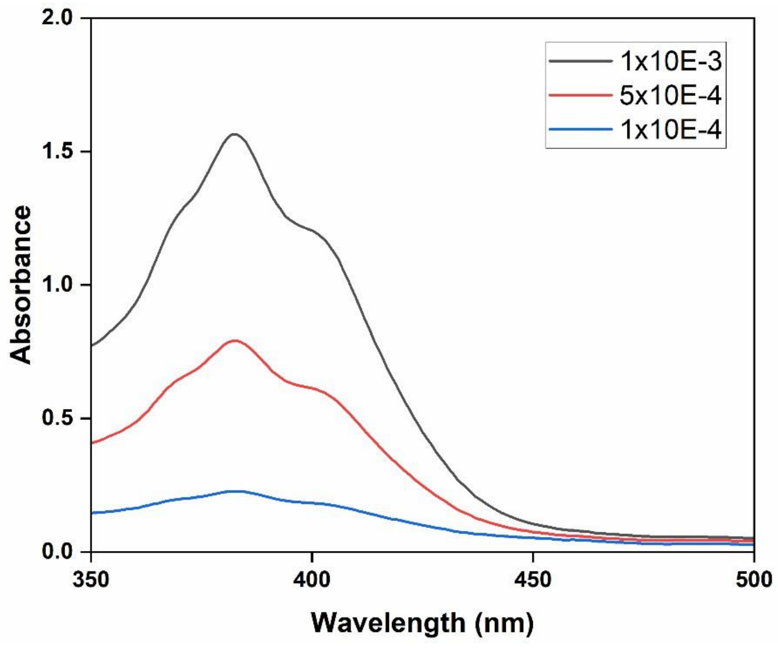

Figure S48. UV-Vis absorption spectrum of compound 1**2**

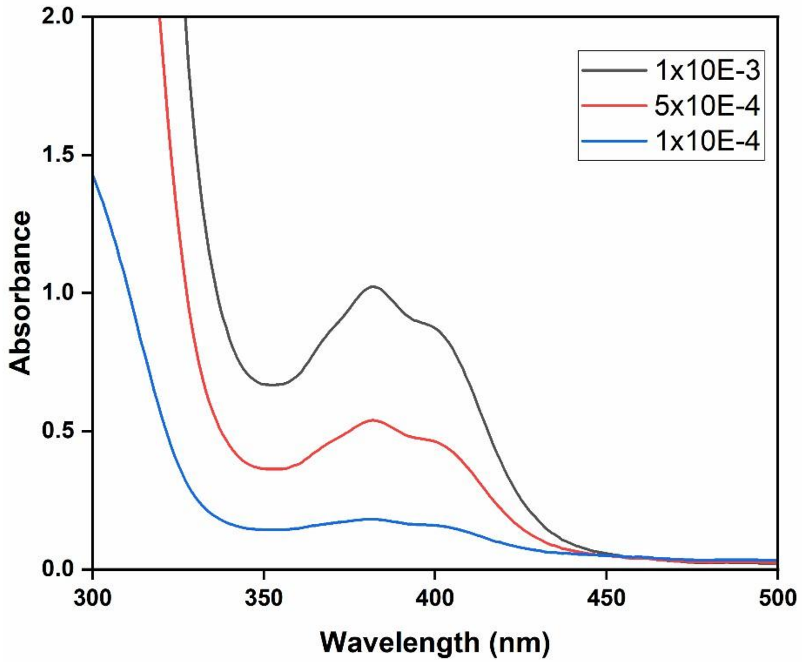

Figure S49. UV-Vis absorption spectrum of compound **15**.

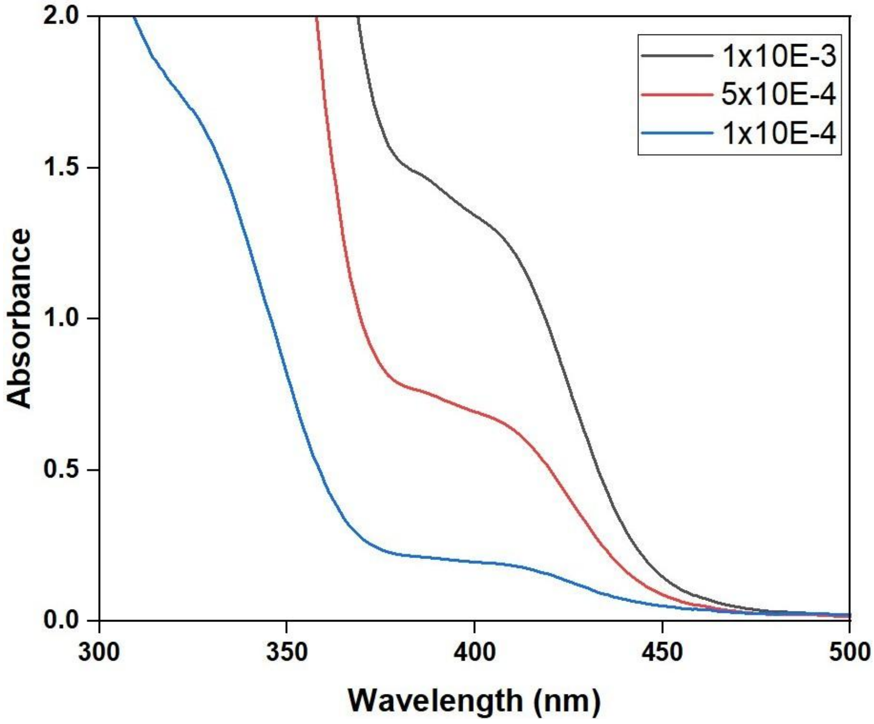

Figure S50. UV-Vis absorption spectrum of compound **17**.

[S5]. X-ray Crystallography

Table S1. Crystallographic data and details of measurements for compounds **2, 5, 9, 10, 11, 12, 15 and 16.**

**Compound (CCDC)**	**2 (2490166)**	**5 (2490167)**	**9 (2490168)**	**10 (2490169)**
**Formula**	$C_{30}H_{33}GeIO_3$	$C_{34}H_{45}ClGeO_3Si_2$	$C_{34}H_{43}ClGe_2O_3$	$C_{42}H_{43}ClGe_2O_3$
**Fw (g mol^{-1})**	641.05	665.92	680.31	776.39
***a* (Å)**	11.8816(2)	8.18020(10)	15.7960(6)	9.7803(3)
***b* (Å)**	17.0326(2)	18.5504(2)	11.0868(4)	32.9602(12)
***c* (Å)**	14.6161(2)	22.0340(2)	20.0632(9)	11.3317(4)
**α (°)**	90	90	90	90
**β (°)**	108.1290(10)	94.7950(10)	110.910(5)	90
**γ (°)**	90	90	90	90
***V* (Å^3)**	2811.09(7)	3331.87(6)	3282.2(2)	3652.9(2)
Z	4	4	4	4
**Crystal size (mm)**	0.14×0.09×0.08	0.21×0.18×0.04		0.41×0.33×0.24
**Crystal habit**	yellow block	yellow plate		clear yellow, block
**Crystal system**	monoclinic	monoclinic	monoclinic	orthorhombic
**Space group**	$P2_1/c$	$P2_1/c$	$P2_1/c$	$P2_12_12_1$
***dcalc* (Mg m^{-3})**	1.515	1.328	1.377	1.412
**μ (mm^{-1})**	10.319	2.925	1.944	1.757
***T* (K)**	100.0(5)	100.00(10)	100.00(10)	100.00(10)
**2θ range (°)**	3.915 to 79.208	3.119 to 80.163	2.745 to 33.124	2.472 to 29.999
***F*(000)**	1288.0	1400.0	1408.0	1600.0
***R*int**	0.0573	0.0420	0.0706	0.0700
**No. of measured and independent [*I* > 2s(*I*)] reflections**	58902, 5873	58376, 7192	79750, 11173	50500, 10618
**No. of parameters, restraints**	326, 0	383, 0	392, 0	443, 0
**Δ›max, Δ›min (e Å^{-3})**	1.670, -1.623	0.524, -0.492	2.217, -1.791	0.996, -0.418
**R1, wR2 (all data)**	0.0434, 0.1245	0.0364, 0.0911	0.1139, 0.1579	0.0534, 0.0824
**R1, wR2 (>2σ)**	0.0426, 0.1238	0.0331, 0.0888	0.0640, 0.1400	0.0401, 0.0789

Compound (CCDC)	11 (2490170)	12 (2490171)	15 (2490172)	16 (2490173)
**Formula**	$C_{42}H_{60}Ge_2O_3$	$C_{42}H_{60}GeO_3Sn$	$C_{48}H_{64}Ge_4O_4$	$C_{28}H_{40}GeO_2Sn$
**Fw (g mol^{-1})**	758.08	804.18	995.35	599.88
a **(Å)**	10.08570(10)	10.10989(13)	9.18200(10)	11.9631(5)
b **(Å)**	13.30020(10)	13.5252(2)	12.8411(2)	11.9878(6)
c **(Å)**	15.65770(10)	15.6456(2)	20.0765(2)	12.5295(7)
α **(°)**	83.3740(10)	83.8903(11)	90	64.111(5)
β **(°)**	89.5710(10)	88.8154(10)	90.8170(10)	62.045(5)
γ **(°)**	72.2140(10)	72.6310(12)	90	89.415(4)
V **(Å^3)**	1985.75(3)	2030.06(5)	2366.92(5)	1384.50(14)
Z	2	2	2	2
**Crystal size (mm)**	0.14×0.09×0.06	0.14 × 0.1 × 0.07	0.07×0.06×0.03	0.06×0.05×0.02
**Crystal habit**	yellow block	yellow plate	Yellow, block	clear yellow, block
**Crystal system**	triclinic	triclinic	monoclinic	triclinic
**Space group**	*P*-1	*P-1*	$P2_1/n$	*P*-1
dcalc **(Mg m^{-3})**	1.268	1.316	1.397	1.439
μ **(mm^{-1})**	2.126	6.061	3.239	8.659
T **(K)**	100.0(3)	99.98(18)	100.00(3)	100.00(3)
**2*θ* range (°)**	2.842 to 80.483	5.682 to 160.172	4.087 to 80.035	4.228 to 74.992
***F*(000)**	800	836.0	1024.0	612.0
***R*int**	0.0434	0.0617	0.0413	0.1480
**No. of measured and independent [*I* > 2s(*I*)] reflections**	69702, 8577	58376, 8687	104533 5105	31991, 5654
**No. of parameters, restraints**	436, 0	436, 0	261 0	368 205
**Δ›max, Δ›min (e Å^{-3})**	0.516, -0.421	0.56, -0.59	0.940 -0.427	1.172 -0.900
**R1, wR2 (all data)**	0.0279, 0.0683	0.0261, 0.0658	0.0414, 0.0931	0.0898, 0.1611
**R1, wR2 (>2*σ*)**	0.0259, 0.0668	0.0257, 0.0654	0.0351, 0.0893	0.0578, 0.1424

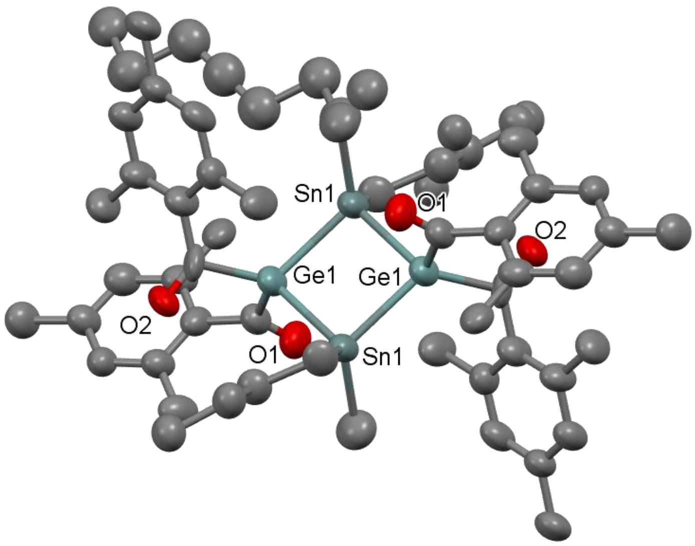

Figure S51. ORTEP representation for compound **16**. Thermal ellipsoids are depicted at the 50% probability level. Hydrogen atoms are omitted, and mesityl groups are displayed as wireframes for clarity. Selected bond lengths (Å) of **16** with estimated standard deviations: Sn1-Ge1 2.639(2), Sn-C22 2.41(4), Sn1-C26 2.28(6) Ge1-C1 2.12(2), Ge1- C11 2.03(2).
